# The endosomal sorting adaptor HD-PTP is required for ephrin-B:EphB signalling in cellular collapse and spinal motor axon guidance

**DOI:** 10.1038/s41598-019-48421-9

**Published:** 2019-08-16

**Authors:** Sylvie Lahaie, Daniel Morales, Halil Bagci, Noumeira Hamoud, Charles-Etienne Castonguay, Jalal M. Kazan, Guillaume Desrochers, Avihu Klar, Anne-Claude Gingras, Arnim Pause, Jean-François Côté, Artur Kania

**Affiliations:** 1 Institut de recherches cliniques de Montréal (IRCM), Montréal, QC H2W 1R7 Canada; 2 Integrated Program in Neuroscience, McGill University, Montréal, QC H3A 2B4 Canada; 3 Department of Anatomy and Cell Biology, McGill University, Montréal, QC H3A 0C7 Canada; 4 Goodman Cancer Research Centre, McGill University, Montréal, QC H3A 1A3 Canada; 5 Department of Biochemistry, McGill University, Montréal, QC H3G 1Y6 Canada; 6 Department of Medical Neurobiology, IMRIC, Hebrew University-Hadassah Medical School, Jerusalem, 91120 Israel; 7 Lunenfeld-Tanenbaum Research Institute, Mount Sinai Hospital, Toronto, ON M5G 1X5 Canada; 8 Department of Molecular Genetics, University of Toronto, Toronto, ON M5S 1A8 Canada; 9 Programmes de Biologie Moléculaire, Département de Médecine, Université de Montréal, Montréal, QC H3T 1J4 Canada; 10 Département de Biochimie, Université de Montréal, Montréal, QC H3C 3J7 Canada; 11 Division of Experimental Medicine, McGill University, Montréal, QC H3A 2B2 Canada; 12 Present Address: Neuroengineering Laboratory, Brain Mind Institute, École polytechnique fédérale de Lausanne (EPFL), Station 19, CH-1015 Lausanne, Switzerland

**Keywords:** Extracellular signalling molecules, Axon and dendritic guidance

## Abstract

The signalling output of many transmembrane receptors that mediate cell-cell communication is restricted by the endosomal sorting complex required for transport (ESCRT), but the impact of this machinery on Eph tyrosine kinase receptor function is unknown. We identified the ESCRT-associated adaptor protein HD-PTP as part of an EphB2 proximity-dependent biotin identification (BioID) interactome, and confirmed this association using co-immunoprecipitation. HD-PTP loss attenuates the ephrin-B2:EphB2 signalling-induced collapse of cultured cells and axonal growth cones, and results in aberrant guidance of chick spinal motor neuron axons *in vivo*. HD-PTP depletion abrogates ephrin-B2-induced EphB2 clustering, and EphB2 and Src family kinase activation. HD-PTP loss also accelerates ligand-induced EphB2 degradation, contrasting the effects of HD-PTP loss on the relay of signals from other cell surface receptors. Our results link Eph function to the ESCRT machinery and demonstrate a role for HD-PTP in the earliest steps of ephrin-B:EphB signalling, as well as in obstructing premature receptor depletion.

## Introduction

Cell-cell contact-dependent signalling underlies many diverse biological processes such as tissue boundary formation, synaptic plasticity, axon guidance, and tumourigenesis. The relatively small family of Eph receptor tyrosine kinases plays a major role in all of these, but the molecular pathways that endow Eph signalling with impressive spatiotemporal precision are still being unravelled^1^. The highly-conserved endosomal sorting complex required for transport (ESCRT) modulates the signalling of many classes of cell surface receptors through their internalisation, lysosomal degradation or recycling^2^. Intriguingly, despite ESCRT’s nearly universal involvement in transmembrane receptor function, its role in Eph signalling remains unexplored.

Eph receptor A and B subfamilies are defined by their ephrin ligands’ linkage to the cell membrane via a GPI anchor or a transmembrane domain, respectively. Forward signalling evoked by ephrin binding to the Eph ligand binding domain (ephrin:Eph) typically results in a rapid and restricted actin cytoskeleton collapse in the Eph expressing cell, underlying cell-cell repulsion at tissue boundaries, cancer cell invasion, and dendritic spine plasticity^3,4^. Arguably, the best understood *in vivo* ephrin:Eph signalling events are those directing axonal growth cones; for example, ephrin-B ligands expressed by the vertebrate dorsal limb mesenchyme, repel EphB-expressing spinal motor neuron axons and direct them to their muscle targets in the ventral limb^5^. At the molecular level, one early critical event in ephrin:Eph signalling is the formation of large Eph multimer arrays upon ephrin binding^6,7^. The induction of Eph clusters is sufficient to induce cytoskeletal collapse^8^, and their size and composition determine the strength of this response^9^. Besides ephrin-Eph contacts, clustering is driven by Eph-Eph interactions via Eph extracellular cysteine-rich domains^6^, intracellular SAM domains^10^ and, possibly, PDZ domain-containing intracellular adaptor proteins^11^. Eph clustering enables the phosphorylation of juxtamembrane tyrosines, which is required for the activation of the Eph kinase domain^8,12^ and the recruitment of intracellular effectors including Src family kinases (SFKs) that link receptor activation to the actin cytoskeleton^13,14^. Despite the critical importance of receptor clustering in the initiation of the Eph signalling cascade, the factors that control it remain virtually unknown.

The endosomal internalisation of ephrin:Eph complexes is required for normal receptor signalling^15–17^, and eventually leads to dephosphorylation of juxtamembrane tyrosines^18^, ubiquitylation of the Eph cytoplasmic tail^19^, and Eph recycling or degradation^20^. It is unknown whether the fate of internalised Eph receptors depends on the ESCRT machinery, which detects ubiquitylated receptors and transfers them between specialised vesicles, where they are sorted back to the membrane or to the lysosome^2,21^. Among the regulators of this progression is the Bro1 domain-containing cytosolic protein, His-domain-containing protein tyrosine phosphatase (HD-PTP, also known as PTPN23 and Myopic), which brings ESCRT proteins directly in contact with the UBPY deubiquitylase^22,23^. HD-PTP loss leads to impaired sorting of internalised receptors and their aberrant accumulation in endosomes^24,25^. Mice heterozygous for *Ptpn23*, the gene encoding HD-PTP, are predisposed to various tumours^26,27^, a phenotype commonly associated with excessive activation of morphogen and growth factor receptors. HD-PTP has not been studied in the context of Eph signalling and, more generally, the only evidence linking Eph signalling to the ESCRT machinery is the observation that EphB2 can associate with ESCRT proteins in the context of exosome biogenesis^28^.

To study the proteomic environment of activated Eph receptors and its relation to Eph clustering and endocytic sorting, we performed a proximity-dependent biotin identification experiment in cells expressing EphB2, exposed to ephrin-B2. Among the identified EphB2-proximal proteins we found HD-PTP, which can interact with EphB2 in a ligand-dependent manner, and is required for EphB2 signalling in the context of cell and axonal growth cone collapse, and in the guidance of spinal motor neuron axons *in vivo*. Our experiments argue that HD-PTP functions in the earliest steps of the Eph signalling cascade: the formation of EphB2 clusters and Src family kinase phosphorylation in response to ephrin-B2 stimulation. HD-PTP also acts at a later step in the Eph signalling pathway where, in contrast to its described function for other receptors processed by ESCRTs^24^, it acts as a negative regulator of EphB2 degradation by the lysosome. Altogether, these results are the first to establish a functional link between Eph signalling and ESCRT accessory proteins, revealing their novel role in promoting cell surface receptor signalling.

## Results

### A BioID interactome of activated EphB2 reveals HD-PTP as a putative effector

**Figure 1:**
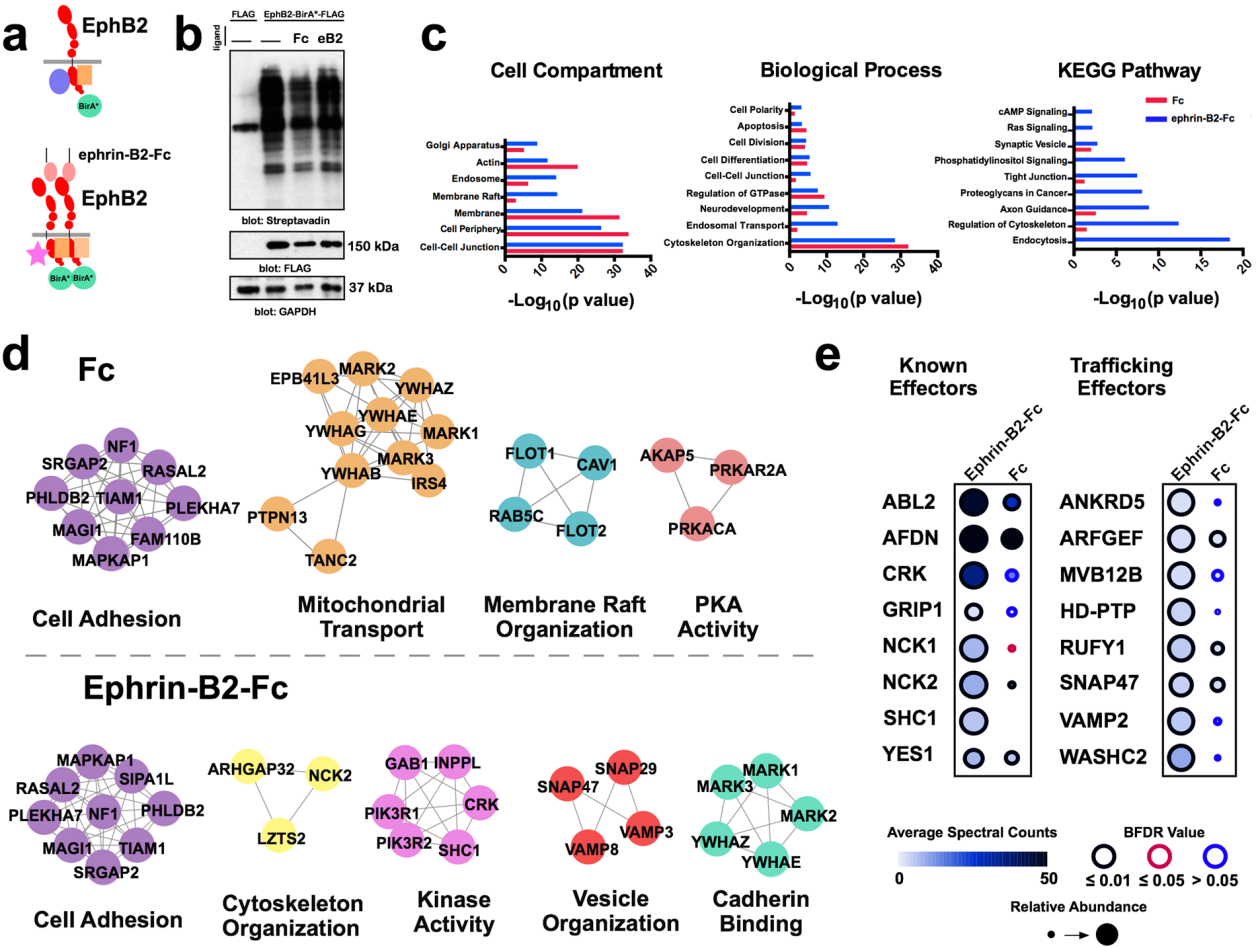
A BioID screen for ligand-stimulated EphB2-proximal proteins. (**a**) Schematic of the BioID experiment: a FLAG-tagged biotin ligase, BirA*, was fused to the C-terminus of EphB2 and stably expressed in HEK293 cells. Depending on the presence of ephrin-B2 ligands, different proteins are recruited to the vicinity of EphB2 and labelled with biotin. (**b**) Western blot of biotinylation in protein lysates from our mass spec samples of HEK293 cells expressing EphB2-BirA*-FLAG (lane 2, no ligand; lane 3, 1.5 µg/mL Fc; and lane 4, 1.5 µg/mL pre-clustered ephrin-B2-Fc) or FLAG alone (lane 1) (*n* = 2). (**c**) Gene ontology and KEGG^85–87^ terms associated with the proteins identified in the eB2 WD-score and Fc WD-score analysis. Blue bars represent proteins enriched in the eB2-treated samples and red bars represent proteins enriched in the Fc-treated samples. (**d**) Interactome webs of proteins identified in the eB2 or Fc WD-score analysis generated by Cytoscape and clustered with MCluster, divided by gene ontology term. (**e**) Dot plots for known EphB2 effector or trafficking-related preys in eB2 and Fc conditions. Spectral count is illustrated by fill shade, relative abundance of the protein compared to the EGFP-BirA*-FLAG condition shown by circle size, and outer circle colour represents BFDR value when compared to EGFP-BirA*-FLAG MS SAINT analysis. Full sized Western blots are in Supplementary Materials. kDa: kilodalton; eB2: ephrin-B2-Fc; BFDR: Bayesian false discovery rate.

To identify new proteins potentially involved in EphB2 receptor activation and its processing, we used proximity-dependent biotin identification (BioID)^29,30^. We generated a Flp-In T-REx HEK293 cell line with inducible expression of EphB2-BirA*-FLAG (EphB2-OE HEK), where the BirA* biotin ligase was fused to the C-terminus of EphB2, allowing us to identify proteins in close proximity to EphB2 during eB2-induced forward signalling. We stimulated these cells with either clustered ephrin-B2-Fc (eB2), Fc, or medium (“no ligand”) for 6 h, followed by lysis, streptavidin pull-down and mass spectrometry (MS; *n* = 4 per condition; Fig. 1a,b). MS data were filtered using Significant Analysis of INTeractome (SAINT)^31^, with BirA*-FLAG-EGFP and empty vector HEK293 MS datasets as controls, yielding prey peptides with a Bayesian false discovery rate (BFDR) score ≤ 0.01 (Full dataset: MSV000083410 at https://massive.ucsd.edu). As a result of removing background noise, we were able to generate two lists of enriched/excluded preys in EphB2 basal signalling (Fc) and EphB2 forward signalling (eB2). We then analysed the eB2 and Fc SAINT datasets by calculating each prey’s WD-Score, a measure of hit specificity^32^. Differences between average spectral counts and WD-scores in eB2 or Fc conditions were found for many preys.

Next, we used g:Profiler to perform functional annotations of the WD-score analysis of eB2 and Fc conditions, which showed an enrichment of proteins associated with endosomal trafficking and neurodevelopmental biological processes in the eB2-stimulated profile^33^ (Fig. 1c). Using the Cytoscape database^34^ and the Markov Cluster (MCL) tool^35^, we generated two interactome maps using Fc and eB2 WD-Score analysis (Fig. 1d). As expected, eB2-stimulated protein clusters include known EphB2 forward signalling functions such as cytoskeleton organisation, kinase activity, and vesicle organisation.

Based on these broad visualisations of our MS data, we examined individual preys more specifically, comparing their average spectral counts, relative abundance, and BFDR score between the eB2 and Fc treatments (Fig. 1e). Several known EphB2-binding proteins were enriched upon eB2 stimulation, such as Abelson kinase (ABL2)^36^ and Nck adaptor proteins (NCK1 & NCK2)^37^, suggesting that our BioID analysis sampled the protein environment of active EphB2 forward signalling. Among the trafficking proteins that were enriched by eB2 stimulation, we found HD-PTP, a known ESCRT adaptor protein with trafficking functions but without previous evidence of involvement in Eph signalling^2^.

### HD-PTP and EphB2 can form a molecular complex

**Figure 2:**
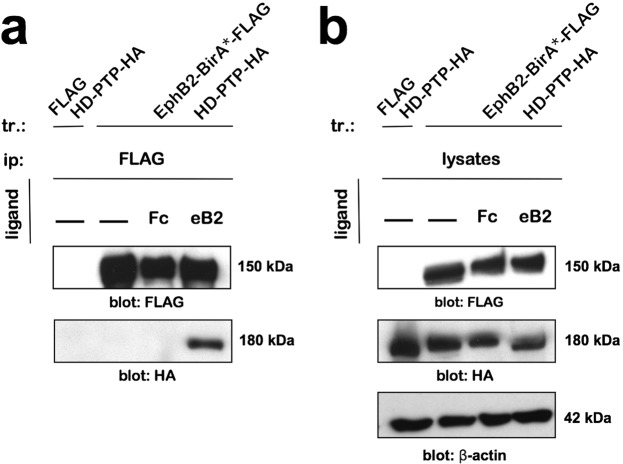
EphB2 and HD-PTP can form a ligand-dependent complex. (**a**) Representative blot of a co-immunoprecipitation experiment performed in HEK293 cells transfected with HD-PTP-HA and expressing either EphB2-BirA*-FLAG or FLAG alone. Blotting for HA shows pull-down of HD-PTP with EphB2 only in cells that have been stimulated for 15 min with 1.5 µg/mL eB2. (**b**) Representative blot of total cell lysates from the co-immunoprecipitation experiment performed in HEK293 cells transfected with HD-PTP-HA and expressing either EphB2-BirA*-FLAG or FLAG alone. Full sized Western blots are in Supplementary Materials. tr: transfection; ip: immunoprecipitation; kDa: kilodalton; eB2: ephrin-B2-Fc.

Although HD-PTP can interact with receptor tyrosine kinases^22^, its association with Eph receptors has not been assayed. Thus, we performed co-immunoprecipitation assays in the EphB2-overexpressing HEK293 cell line transfected with an HD-PTP-HA expression plasmid. Following EphB2-BirA*-FLAG expression induction, the cells were treated with ephrin-B2-Fc, Fc, or medium, and lysed. The EphB2-FLAG-directed pull-down produced an HD-PTP-HA band following eB2 stimulation, but not in Fc or medium conditions (Fig. 2a,b; *n* = 4; Supplementary Fig. S1), showing that HD-PTP can form a ligand-induced complex with EphB2.

### HD-PTP is required for ephrin-B2-induced HeLa cell collapse

**Figure 3:**
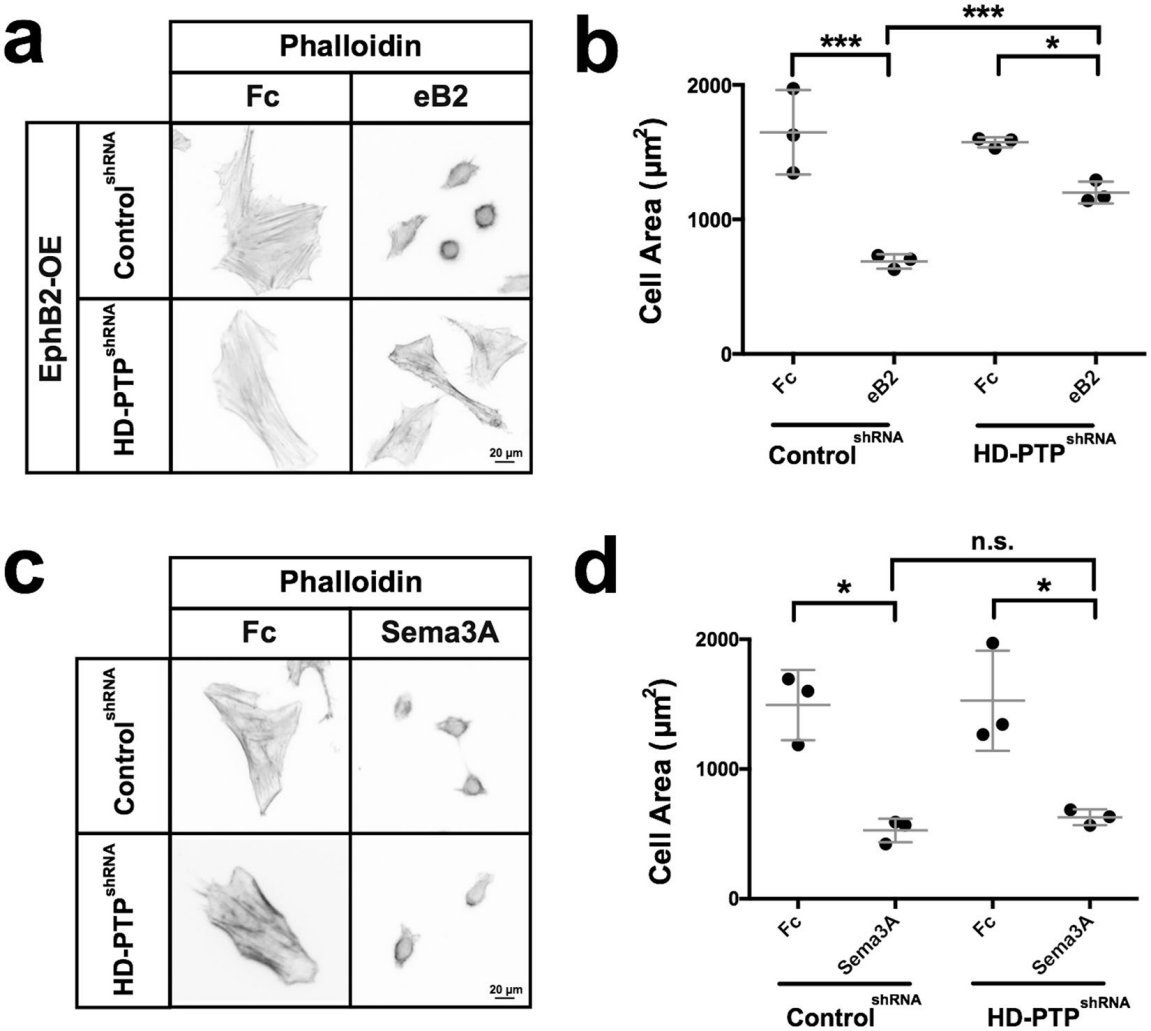
HD-PTP is required for ephrin-B2-induced cell collapse. (**a**) Representative images of Alexa Fluor 568-conjugated phalloidin-stained Control^shRNA^ and HD-PTP^shRNA^ HeLa cells transfected with EphB2-GFP, and stimulated 15 min with 1 µg/mL eB2 or Fc. (**b**) Quantification of HeLa cell area shows that Control^shRNA^ EphB2-OE cells collapse to about half their size in response to 1 µg/mL eB2, while HD-PTP^shRNA^ EphB2-OE cells collapse only by ~20% (*n* = 3, 60–80 cells/*n*; Control^shRNA^, *p* = 0.0003; HD-PTP^shRNA^, *p* = 0.0008; Student’s *t-*test). (**c**) Representative images of Alexa Fluor 568-conjugated phalloidin stains of Control^shRNA^ and HD-PTP^shRNA^ HeLa cells treated with 0.3 µg/mL Sema3A-Fc or Fc for 15 min. (**d**) Quantification of HeLa cell area shows Control^shRNA^ HeLa cells collapse to less than half their size in response to 0.3 µg/mL Sema3A-Fc, and HD-PTP^shRNA^ HeLa cells collapse to the same extent (*n* = 3, 60–80 cells/*n*; Control^shRNA^ *vs*. HD-PTP^shRNA^, *p* = 0.3880; Student’s *t-*test). Values are plotted as mean ± SD. All values can be found in Supplementary Table S4. eB2: ephrin-B2-Fc; ***p < 0.001; *p < 0.05; n.s.: not significant. Inverted grayscale fluorescent images.

We next assessed whether HD-PTP functions in ephrin-B2:EphB2 signalling. We first turned to the HeLa cell collapse assay, a model that has been used to quantify the repulsive effects of ephrin signalling^38^. We took advantage of an HD-PTP^shRNA^ cell line^25^, which has a 50% reduction in HD-PTP protein compared to control (Supplementary Fig. S1). We transfected Control^shRNA^ and HD-PTP^shRNA^ HeLa cells with an EphB2-GFP fusion expression plasmid (Supplementary Fig. S1; EphB2-OE) and stimulated them with eB2 or Fc. The size of Control^shRNA^ EphB2-OE cells treated with eB2 decreased by ~50% compared to Fc treatment (Fig. 3a,b; *n* = 3; *p* = 0.0003), but eB2 treatment reduced the size of HD-PTP^shRNA^ EphB2-OE cells by only 25% compared to control treatment (Fig. 3a,b; *n* = 3; Control^shRNA^ eB2 *vs*. HD-PTP^shRNA^ eB2, *p* = 0.0008). To determine whether this blunted response was specific to eB2 stimulation, Control^shRNA^ and HD-PTP^shRNA^ cells were exposed to Sema3A, another collapse-inducing chemotropic factor acting through neuropilin and plexin expressed by HeLa cells^39,40^. Both cell lines collapsed to an equal extent when stimulated with Sema3A (Fig. 3c,d; *n* = 3; Control^shRNA^ *vs*. HD-PTP^shRNA^ *n.s*., *p* = 0.3880), indicating that the loss of HD-PTP attenuates the eB2-induced collapse of HeLa cells, but does not affect the response to an unrelated cell collapse-inducing signal.

### HD-PTP expression in spinal motor neurons

**Figure 4:**
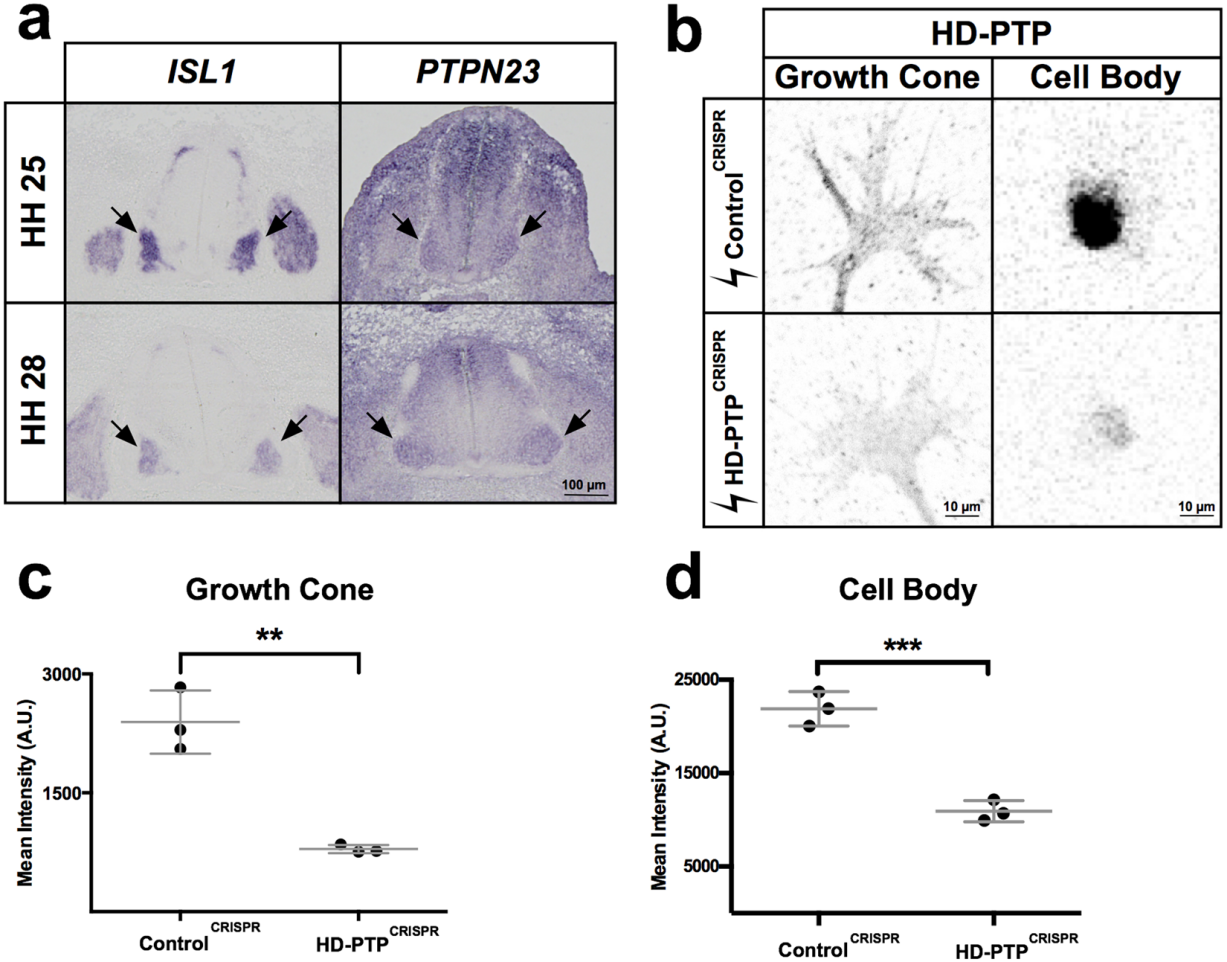
HD-PTP expression in embryonic motor neurons and CRISPR-mediated depletion. (**a**) Representative images of chick embryonic spinal cord sections at HH st. 25 and HH st. 28 where *ISL1* and *PTPN23* (chicken HD-PTP-encoding gene) mRNA was detected using *in situ* hybridisation. Note expression of *PTPN23* in *ISL1*-expressing motor column (arrows). (**b**) Representative images of anti-HD-PTP antibody staining in growth cones and cell bodies of dissociated motor neurons harvested from embryonic spinal cords electroporated with Control^CRISPR^ or HD-PTP^CRISPR^ plasmids. (**c**) Quantification of HD-PTP signals in growth cones of dissociated motor neurons harvested from embryonic spinal cords shows a decreased signal in HD-PTP^CRISPR^ compared to Control^CRISPR^ (*n* = 3, 10–12 growth cones/*n*; *p* = 0.0023; Student’s *t-*test). (**d**) Quantification of HD-PTP signal in cell bodies of dissociated motor neurons harvested from embryonic spinal cords show decrease signal in HD-PTP^CRISPR^ compared to Control^CRISPR^ (*n* = 3, 30–50 cell bodies/*n*; *p* = 0.0009; Student’s *t-*test). Values are plotted as mean ± SD. All values can be found in Supplementary Table S4. ***p < 0.001; **p < 0.01. Visible light images (**a**) and inverted grayscale fluorescent images (**b**).

Ephrin-B:EphB signalling is required for the guidance of embryonic spinal motor axons to their limb targets^5,41^, raising the possibility that HD-PTP may also be required for this process. First, we visualised *PTPN23* (HD-PTP) mRNA in embryonic chick spinal cord at Hamburger and Hamilton stages (HH st.) 25 and 28, when spinal lateral motor column (LMC) axons are guided by ephrin-B:EphB signalling^5,42^. At these stages, *PTPN23* mRNA was expressed broadly in the dorsal spinal cord, as well as in motor neurons defined by *ISL1* mRNA expression^43^ (Fig. 4a); however, mRNAs encoding the closely related phosphatases PTPN13 and PTPN14 were not detected in the spinal cord at similar ages (Supplementary Fig. S2).

### HD-PTP is required for ephrin-B2-induced LMC growth cone collapse

To test whether HD-PTP is required for normal ephrin-B:EphB signalling in LMC neurons, we induced HD-PTP loss-of-function in LMC motor neurons using CRISPR-Cas9^44,45^. We designed two guide RNAs targeting exon 2 and one targeting exon 5 of the *PTPN23* gene, to increase the likelihood of coding sequence double-stranded breaks and frameshifts due to error-prone Cas9 non-homologous end joining^46,47^ (Supplementary Fig. S2). We co-electroporated three plasmids, each encoding one guide RNA, a Cas9-FLAG fusion protein, and GFP expressed using the T2A self-cleaving peptide system, into HH st. 18/19 chick neural tubes^48^ and harvested HD-PTP^CRISPR^ spinal cords at HH st. 25. As a control, we used a plasmid encoding Cas9-FLAG, GFP, and a guide RNA targeting an untranslated region of the *EPHA4* gene (Control^CRISPR^). A deletion in the *PTPN23* locus, consistent with a removal of the sequence between guides 1 and 3, was revealed by PCR amplification of genomic DNA extracted from HD-PTP^CRISPR^, but not from Control^CRISPR^ spinal cords (Supplementary Fig. S2). When HH st. 25 HD-PTP^CRISPR^ and Control^CRISPR^ ventral spinal cord neurons were explanted and cultured *in vitro* for at least 18 hours, HD-PTP signal in HD-PTP^CRISPR^ growth cones and cell bodies was significantly decreased compared to Control^CRISPR^ controls (Fig. 4b–d; *n* = 3; growth cone, *p* = 0.0023; cell body, *p* = 0.0009).

**Figure 5:**
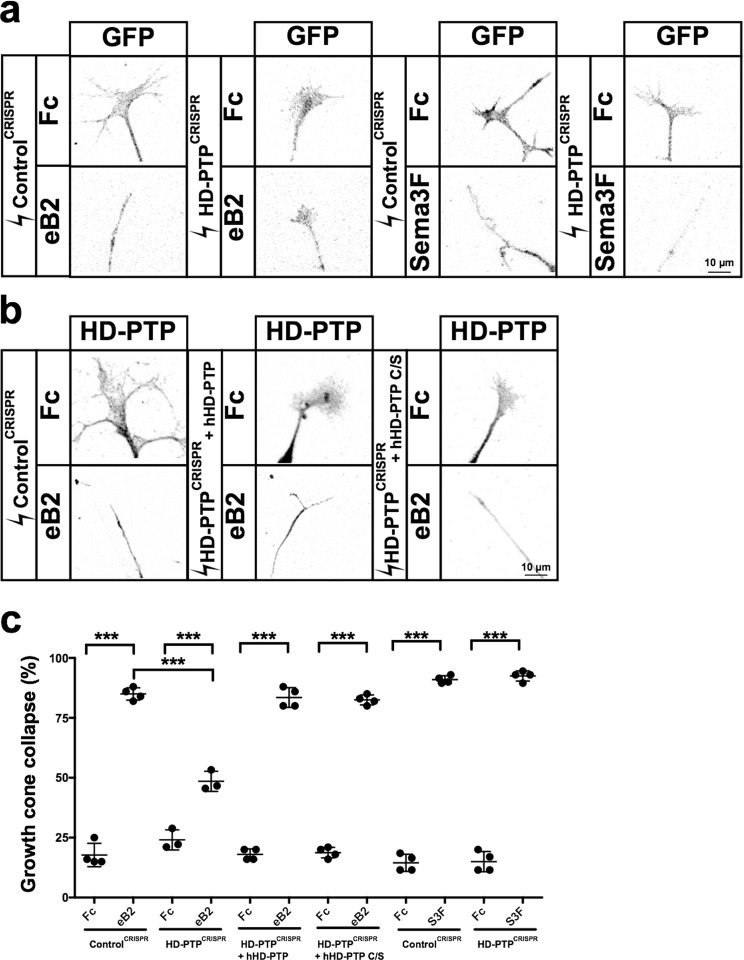
Spinal motor axon growth cones require HD-PTP for ephrin-B2-induced collapse. (**a**) Representative images of GFP^+^ growth cones from dissociated Control^CRISPR^- or HD-PTP^CRISPR^-electroporated motor neurons, incubated with eB2, Sema3F, or Fc, and stained with anti-GFP antibodies. (**b**) Representative images of growth cones from rescue experiments using dissociated motor neurons electroporated with Control^CRISPR^ plasmid or HD-PTP^CRISPR^ co-electroporated with hHD-PTP or hHD-PTP C/S plasmid, incubated 30 min with 10 µg/mL eB2 or Fc and stained with anti-HD-PTP antibodies. (**c**) Quantification of collapsed growth cones in dissociated motor neurons electroporated with CRISPR constructs and stimulated with ligands. HD-PTP^CRISPR^ or Control^CRISPR^ growth cones were incubated for 30 min with 10 µg/mL eB2 or Fc. The collapse response of HD-PTP^CRISPR^ growth cones to eB2 was significantly attenuated compared to Control^CRISPR^ (*n* = 3, 90 growth cones/*n*; *p* < 0.0001; Fisher’s exact test). Rescue experiments with growth cones from dissociated motor neurons electroporated with Control^CRISPR^, or HD-PTP^CRISPR^ co-electroporated with hHD-PTP or hHD-PTP C/S expression plasmid, incubated 30 min with 10 µg/mL eB2 or Fc and stained with anti-HD-PTP and anti-Isl1 antibodies. Both populations responded to eB2 treatment indistinguishably from control (*n* = 4, 50 growth cones/*n*; Fisher’s exact test). HD-PTP^CRISPR^ or Control^CRISPR^ growth cones were incubated for 30 min with 0.3 µg/mL Sema3F-Fc or Fc. The two CRISPR growth cone populations behaved identically, demonstrating that HD-PTP loss does not affect the response to Sema3F (*n* = 4, 30 growth cones/*n*; Fisher’s exact test). Values are plotted as mean ± SD. All values can be found in Supplementary Table S4. h: human; S3F: Sema3F; eB2: ephrin-B2-Fc; ***p < 0.001; n.s.: not significant. Inverted grayscale fluorescent images.

Such cultured HD-PTP^CRISPR^ and Control^CRISPR^ neurons did not differ in their capacities to form growth cones (Fig. 4b), extend axons (Supplementary Fig. S3), or express EphB2 (Supplementary Fig. S3). To determine whether HD-PTP is required for LMC growth cone eB2:EphB2 signalling, we focused on the medial subpopulation of LMC neurons, which express high levels of EphB2 and are repelled by eB2 *in vivo* and *in vitro*^5,49^. HD-PTP^CRISPR^ or Control^CRISPR^ LMC neurons were dissociated and medial LMC neurons were identified via their expression of the transcription factor Isl1^43^. Control^CRISPR^ medial LMC growth cones collapsed significantly when treated with eB2, but HD-PTP^CRISPR^ medial LMC growth cones showed a markedly attenuated collapse response (Fig. 5a,c; *p* < 0.0001). This effect was specific to eB2 treatment, since HD-PTP^CRISPR^ and Control^CRISPR^ growth cones collapsed to the same extent when exposed to Sema3F, a protein known to repel medial LMC axons^50^ (Fig. 5a,c).

To further characterise the specificity of the HD-PTP knockdown, we carried out rescue experiments by co-electroporating a human (h) HD-PTP expression plasmid together with the chick-specific HD-PTP^CRISPR^ plasmids as above. In medial LMC neurons co-electroporated with HD-PTP^CRISPR^ and hHD-PTP expression plasmids, HD-PTP protein levels returned close to control levels (Supplementary Fig. S3), and consequently their collapse response to eB2 returned to control levels (Fig. 5b,c). We also asked whether the HD-PTP phosphatase domain is required for its function in growth cone collapse by co-electroporating HD-PTP^CRISPR^ plasmids together with a plasmid encoding a human HD-PTP with a point mutation disrupting the phosphatase active site^51^ (hHD-PTP C/S). This mutant HD-PTP was capable of rescuing the HD-PTP^CRISPR^–induced growth cone collapse defect (Fig. 5b,c), suggesting that HD-PTP’s function downstream of EphB2 does not require phosphatase activity.

### HD-PTP is required for ephrin-B2-induced SFK activation, EphB2 phosphorylation, and EphB2 surface patching

**Figure 6:**
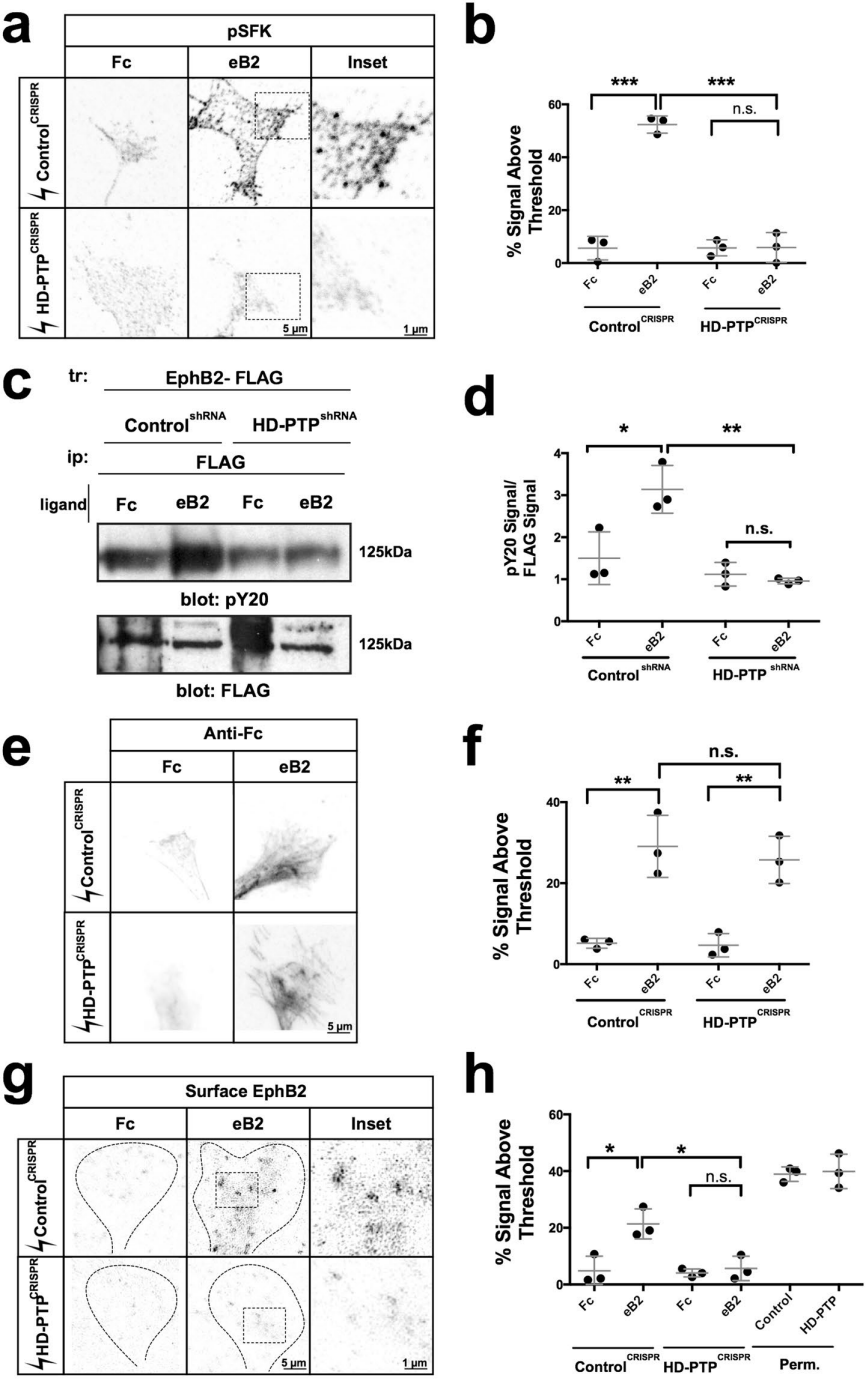
HD-PTP is required for ephrin-B2-induced SFK activation, EphB2 phosphorylation, and EphB2 surface patching. (**a**) Representative images of Control^CRISPR^ and HD-PTP^CRISPR^ spinal motor neuron growth cones, incubated for 15 min with 10 µg/mL eB2 or Fc and stained for with anti-phospho-Y418-SFK, revealing SFK activation following eB2 exposure. (**b**) Quantification of anti-phospho-Y418-SFK signal in Control^CRISPR^ and HD-PTP^CRISPR^ motor neuron growth cones, incubated for 15 min with 10 µg/mL eB2 or Fc. Control^CRISPR^ growth cones showed a ligand-induced increase in SFK activation (*p* < 0.0001), but HD-PTP^CRISPR^ growth cones did not (*p* = 0.9810) (*n* = 3, 10–12 growth cones/*n*; one-way ANOVA followed by corrected Student’s *t-*tests). (**c**) Representative Western blot using anti-phosphotyrosine and anti-FLAG antibodies after pull-downs of EphB2 (with anti-FLAG antibodies) in Control^shRNA^ and HD-PTP^shRNA^ HeLa cells, stimulated with 1 µg/mL eB2 or Fc for 5 min. The band size corresponds to EphB2. (**d**) Quantification of phosphotyrosine signal over FLAG signal shows ligand-induced phosphorylation of EphB2 in Control^shRNA^ HeLa cells (*p* = 0.0284), but not in HD-PTP^shRNA^ cells (*p* = 0.3908) (*n* = 3; one-way ANOVA followed by Student’s *t-*tests). (**e**) Representative images of Control^CRISPR^ and HD-PTP^CRISPR^ spinal motor neuron growth cones, incubated for 2 min with unclustered 10 µg/mL eB2 or Fc and stained with anti-Fc antibodies showing increased anti-Fc staining following eB2 exposure. (**f**) Quantification of anti-Fc signal in Control^CRISPR^ and HD-PTP^CRISPR^ motor neuron growth cones, incubated for 2 min with 10 µg/mL of unclustered eB2 or Fc. Control^CRISPR^ growth cones showed a ligand-induced increase in anti-Fc signal (*p* = 0.0011), and HD-PTP^CRISPR^ growth cones did show an increase as well upon eB2 stimulation (*p* = 0.0028) (*n* = 3, 10–12 growth cones/*n*; one-way ANOVA followed by corrected Student’s *t-*tests). (**g**) Representative images of Control^CRISPR^ and HD-PTP^CRISPR^ motor neuron growth cones, incubated for 15 min with 10 µg/mL eB2 or Fc and immunostained for EphB2 using non-permeabilising fixation conditions. EphB2 patching is visualised through increased signal intensity of surface anti-EphB2 staining. (**h**) Quantification of EphB2 patching in Control^CRISPR^ and HD-PTP^CRISPR^ motor neuron growth cones, incubated for 15 min with 10 µg/mL eB2 or Fc, as measured by percentage of the growth cone area containing surface EphB2 signal. In contrast to Control^CRISPR^ growth cones (*p* = 0.017), HD-PTP^CRISPR^ growth cones failed to elicit EphB2 surface patching upon ligand binding (*p* = 0.5707) (*n* = 3; one-way ANOVA followed by corrected Student’s *t-*tests). Values are plotted as mean ± SD. All values can be found in Supplementary Table S4. Full sized Western blots are in Supplementary Materials. tr: transfection; ip: immunoprecipitation; kDa: kilodalton; eB2: ephrin-B2-Fc; perm: permeabilised; ***p < 0.001; **p < 0.01 *p < 0.05; n.s.: not significant. Inverted grayscale fluorescent images.

One of the earliest events of Eph forward signalling is the phosphorylation of SFKs on their activating tyrosine at position 418 (phospho-Y418-SFK)^52^, an event that can be detected by a specific antiserum^53^ in LMC neurons^41^. To examine whether this SFK phosphorylation event requires HD-PTP, we used this antiserum on fixed HD-PTP^CRISPR^ and Control^CRISPR^ medial LMC growth cones that were exposed to eB2 or Fc. Control^CRISPR^ LMC growth cones treated with eB2 displayed increased levels of phospho-Y418-SFK signal compared to Fc-treated growth cones (Fig. 6a,b; *n* = 3; *p* < 0.0001). In contrast, HD-PTP^CRISPR^ LMC growth cones did not show this effect (Fig. 6a,b; *p* = 0.9810). We replicated this result in our Control and HD-PTP^shRNA^ HeLa cells, and further characterised their SFK phosphorylation dynamics by Western blot. While total Src levels remained constant in the two cell lines after ligand stimulation, ephrin-B2-induced phosphorylation of SFKs was evident in Control^shRNA^ cells, but was abolished in HD-PTP^shRNA^ cells (Supplementary Fig. S4). Thus, the loss of HD-PTP abolishes the activation of a critical effector of ephrin:Eph signalling.

Eph signalling also involves the autophosphorylation of a juxtamembrane tyrosine residue critical for Eph kinase activity^14^. To determine whether HD-PTP is important for this, we transfected Control^shRNA^ and HD-PTP ^shRNA^ HeLa cells with an EphB2-FLAG expression plasmid and stimulated with eB2 or Fc. We then lysed the cells and performed anti-FLAG pull-down and immunoblotted with an anti-phosphotyrosine antibody. Compared to Fc treatment, a significant increase in EphB2 phosphorylation was observed following eB2 stimulation in Control^shRNA^ cells (Fig. 6c,d; *n* = 3*; p* = 0.0284); however, this effect was absent in HD-PTP^shRNA^ cells (Fig. 6c,d; *n* = 3; *p* = 0.3908).

EphB2 autophosphorylation requires the formation of receptor-ligand multimer arrays on the cell surface^9,11,38^. To examine the possibility that this process is HD-PTP dependent, we first confirmed that cell surface expression of EphB2 is not changed upon loss of HD-PTP. Overlay of non-permeabilised HeLa cells and LMC growth cones with unclustered ephrin-B2-Fc revealed similar levels of ligand binding under HD-PTP loss and control conditions, suggesting normal availability of surface EphB receptors (Fig. 6e,f; Supplementary Fig. S4). Detection of cell surface EphB2 in control LMC growth cones and HeLa cells revealed that clustered eB2 treatment resulted in significant EphB2 cell surface patch formation, a correlate of Eph receptor multimers^38^ (Fig. 6g,h; *p* = 0.017 *vs*. Fc; Supplementary Fig. S4). In contrast, loss of HD-PTP resulted in a conspicuous absence of eB2-induced EphB2 cell surface patches in LMC growth cones (Fig. 6g,h; *p* = 0.5707) and HeLa cells (Supplementary Fig. S4). These experiments suggest a critical role for HD-PTP in eB2-induced surface clustering of EphB2.

### HD-PTP protects EphB2 from ligand-induced lysosomal degradation

**Figure 7:**
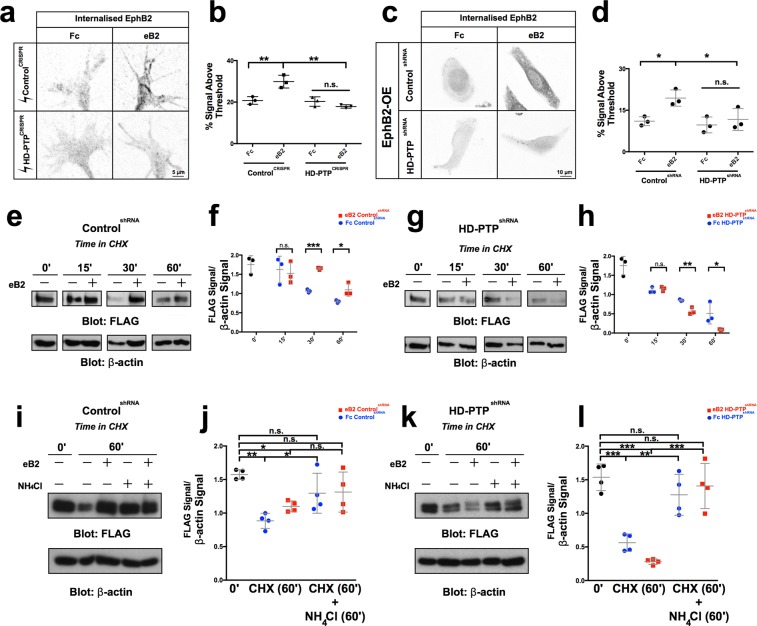
HD-PTP loss increases the rate of EphB2 lysosomal degradation. (**a**) Representative images of Control^CRISPR^ and HD-PTP^CRISPR^ spinal motor neuron growth cones, incubated for 20 min with 10 µg/mL eB2 or Fc and stained with anti-EphB2 antibodies followed by an acid stripping to show internalised EphB2. (**b**) Quantification of internalised EphB2 in Control^CRISPR^ and HD-PTP^CRISPR^ motor neuron growth cones, incubated for 20 min with 10 µg/mL eB2 or Fc. Control^CRISPR^ growth cones showed a ligand-induced increase in internalised EphB2 (*p* = 0.0023), but HD-PTP^CRISPR^ growth cones did not (*p* = 0.9831) (*n* = 3, 10–12 growth cones/*n*; one-way ANOVA followed by corrected Student’s *t-*tests). (**c**) Representative images of Control^shRNA^ and HD-PTP^shRNA^ HeLa cells, incubated for 10 min with 1 µg/mL eB2 or Fc and stained with anti-EphB2 antibodies followed by an acid stripping to show internalised EphB2. (**d**) Quantification of internalised EphB2 staining in Control^shRNA^ and HD-PTP^shRNA^ HeLa cells incubated for 10 min with 1 µg/mL eB2 or Fc. Control^shRNA^ showed an increase in internalised EphB2 signal upon eB2 stimulation (*p* = 0.0216), yet HD-PTP^shRNA^ HeLa cells display no detectable increase in internalised EphB2 (*p* = 0.9001) (*n* = 3, 10–12 cells/*n*; one-way ANOVA followed by corrected Student’s *t-*tests). (**e**) Representative Western blot for EphB2 expression detected with anti-FLAG antibodies in transfected Control^shRNA^ HeLa cell lysates at different time points after incubation with 10 µg/mL protein synthesis blocker cycloheximide, exposed to either 1 µg/mL eB2 or Fc. β-actin detection is used as an internal control. (**f**) Quantification of Western blots for EphB2 detected with anti-FLAG antibodies in transfected Control^shRNA^ HeLa cell lysates after incubation with 10 µg/mL protein synthesis blocker cycloheximide together with either 1 µg/mL eB2 or Fc. FLAG signal intensity was normalised to β-actin and plotted for the different time points. By 30 min after cycloheximide treatment, eB2 stimulation appears to protect EphB2 from degradation compared to Fc (*n* = 3; Student’s *t-*test). (**g**) Representative Western blot for EphB2 detected with anti-FLAG antibodies in transfected HD-PTP^shRNA^ HeLa cell lysates at different time points after incubation with 10 µg/mL protein synthesis blocker cycloheximide and either 1 µg/mL eB2 or Fc. β-actin is used as an internal control. (**h**) Quantification of Western blots for EphB2 detected with anti-FLAG antibodies in transfected HD-PTP^shRNA^ HeLa cell lysates after incubation with 10 µg/mL protein synthesis blocker cycloheximide together with either 1 µg/mL eB2 or Fc. FLAG signal intensity was normalised to β-actin and plotted for the different time points. In contrast to Control^shRNA^ HeLa cells, in HD-PTP^shRNA^ HeLa cells, eB2 stimulation appears to increase rate of EphB2 degradation compared to Fc by 30 min after cycloheximide treatment (*n* = 3; Student’s *t-*test). (**i**) Representative Western blot for EphB2 expression detected with anti-FLAG antibodies in transfected Control^shRNA^ HeLa cell lysates at different time points after incubation with 10 µg/mL protein synthesis blocker cycloheximide along with lysosomal blocker 10 mM NH_4_Cl, exposed to either 1 µg/mL eB2 or Fc. β-actin detection is used as an internal control. (**j**) Quantification of Western blots for EphB2 detected with anti-FLAG antibodies in transfected Control^shRNA^ HeLa cell lysates after incubation with 10 µg/mL protein synthesis blocker cycloheximide along with lysosomal blocker 10 mM NH_4_Cl, exposed to either 1 µg/mL eB2 or Fc. FLAG signal intensity was normalised to β-actin and plotted for the different time points. By 60 min after cycloheximide treatment and lysosomal blocking, EphB2 from degradation levels appear to be rescued (*n* = 4; Student’s *t-*test). (**k**) Representative Western blot for EphB2 expression detected with anti-FLAG antibodies in transfected HD-PTP^shRNA^ HeLa cell lysates at different time points after incubation with 10 µg/mL protein synthesis blocker cycloheximide along with lysosomal blocker 10 mM NH_4_Cl, exposed to either 1 µg/mL eB2 or Fc. β-actin detection is used as an internal control. (**l**) Quantification of Western blots for EphB2 detected with anti-FLAG antibodies in transfected HD-PTP^shRNA^ HeLa cell lysates after incubation with 10 µg/mL protein synthesis blocker cycloheximide along with lysosomal blocker 10 mM NH_4_Cl, exposed to either 1 µg/mL eB2 or Fc. FLAG signal intensity was normalised to β-actin and plotted for the different time points. By 60 min after cycloheximide treatment and lysosomal blocking, EphB2 from degradation levels appear to be rescued (*n* = 4; Student’s *t-*test). Values are plotted as mean ± SD. All values can be found in Supplementary Table S4. Full sized Western blots are in Supplementary Materials. CHX: cycloheximide; eB2: ephrin-B2-Fc; ***p < 0.001; **p < 0.01; *p < 0.05; n.s.: not significant. Inverted grayscale fluorescent images.

As a component of the ESCRT complex, HD-PTP modulates the endocytic sorting of ligand-bound cell surface receptors to degradation pathways and recycling endosomes^24,54^. To examine whether HD-PTP is involved in such processing of Eph receptors, we first used an assay to quantify internalised receptor in LMC growth cones with either normal or reduced levels of HD-PTP, under basal or eB2 stimulated conditions. EphB2 receptor at the cell surface was labelled, and after 10 minutes of ligand stimulation all membrane proteins were acid-stripped, followed by fixing and staining. HD-PTP^CRISPR^ growth cones showed a striking absence of internalised EphB2 after eB2 treatment (Fig. 7a,b; *n* = 3; *p* = 0.9831) compared to Control^CRISPR^ growth cones (Fig. 7a,b; *n* = 3; *p* = 0.0023). This effect was also seen in HeLa cells with a loss of HD-PTP function: Control^shRNA^ cells stimulated with eB2 contained significant amounts of internalised EphB2 compared to Fc (Fig. 7c,d; *n* = 3; *p* = 0.0216), while HD-PTP^shRNA^ cells had negligible levels of internalised EphB2 even when stimulated (Fig. 7c,d; *n* = 3; *p* = 0.9001), suggesting that HD-PTP depletion either decreases EphB2 internalisation or accelerates internalised EphB2 degradation.

To resolve between these possibilities, we compared EphB2 protein levels following protein synthesis inhibition in the presence or absence of eB2, in cells with diminished HD-PTP levels. To do this, we transfected EphB2-FLAG expression plasmids into Control^shRNA^ and HD-PTP^shRNA^ HeLa cells, treated them with eB2 or Fc in the presence of the protein synthesis inhibitor cycloheximide (CHX) and measured dynamic changes in EphB2 protein levels via FLAG immunoblotting^25^. Fc-treated Control^shRNA^ HeLa cells maintained a steady level of EphB2 until about 30 minutes after CHX addition, when EphB2 levels began to decrease (Fig. 7e,f). When incubated with eB2 and CHX, however, EphB2 levels remained steady for up to 60 minutes (Fig. 7e,f; *p* = 0.0018), suggesting that eB2 exposure may inhibit EphB2 degradation. In contrast, 30 minutes after Fc and CHX exposure, HD-PTP^shRNA^ HeLa cells had lower EphB2 levels compared to Fc-treated Control^shRNA^ HeLa cells (Fig. 7e–h; *n* = 3; *p* = 0.002). Furthermore, eB2 and CHX treatment of HD-PTP^shRNA^ HeLa cells resulted in an even more rapid decrease of EphB2 levels, with their almost complete depletion after 60 minutes of treatment (Fig. 7g,h; *n* = 3; 60-min Control^shRNA^ eB2 vs. 60-min HD-PTP^shRNA^ eB2, *p* = 0.0005). These results suggest that HD-PTP is required to prevent the degradation of activated EphB2.

Next, we repeated these analyses in the presence of the lysosomal blocker NH_4_Cl, to determine whether EphB2 in this system is being degraded by the lysosome. In Fc-treated Control^shRNA^ cells, NH_4_Cl treatment significantly increased the amount of EphB2 observed after 60 minutes of CHX treatment (Fig. 7i,j; *n* = 4; *p* = 0.0416). Indeed, EphB2 levels following Fc or eB2 treatment after 60 minutes of CHX and NH_4_Cl treatment were not significantly different from levels at time zero (Fig. 7i,j; *n* = 4; *p* = 0.2173), suggesting that basal degradation of EphB2 is lysosome-dependent. In HD-PTP^shRNA^ HeLa cells, the accelerated EphB2 depletion seen under basal, but especially under ephrin-B2-stimulated conditions, was also abolished by NH_4_Cl treatment (Fig. 7k,l; *n* = 4; 60 min Fc vs 60 min NH_4_Cl Fc, *p* = 0.0051; 60 min eB2 vs 60 min NH_4_Cl eB2, *p* = 0.0005). Together, these experiments demonstrate that the loss of HD-PTP has differential effects on the levels of surface receptors: in contrast to its reported effects on other receptor tyrosine kinases^24^, HD-PTP depletion accelerates EphB2 lysosomal degradation, particularly following its activation by ligand stimulation, suggesting that HD-PTP’s function is to protect EphB2 signalling from termination.

### HD-PTP is required for ephrin-B2:EphB2-mediated medial LMC guidance *in vivo*

**Figure 8:**
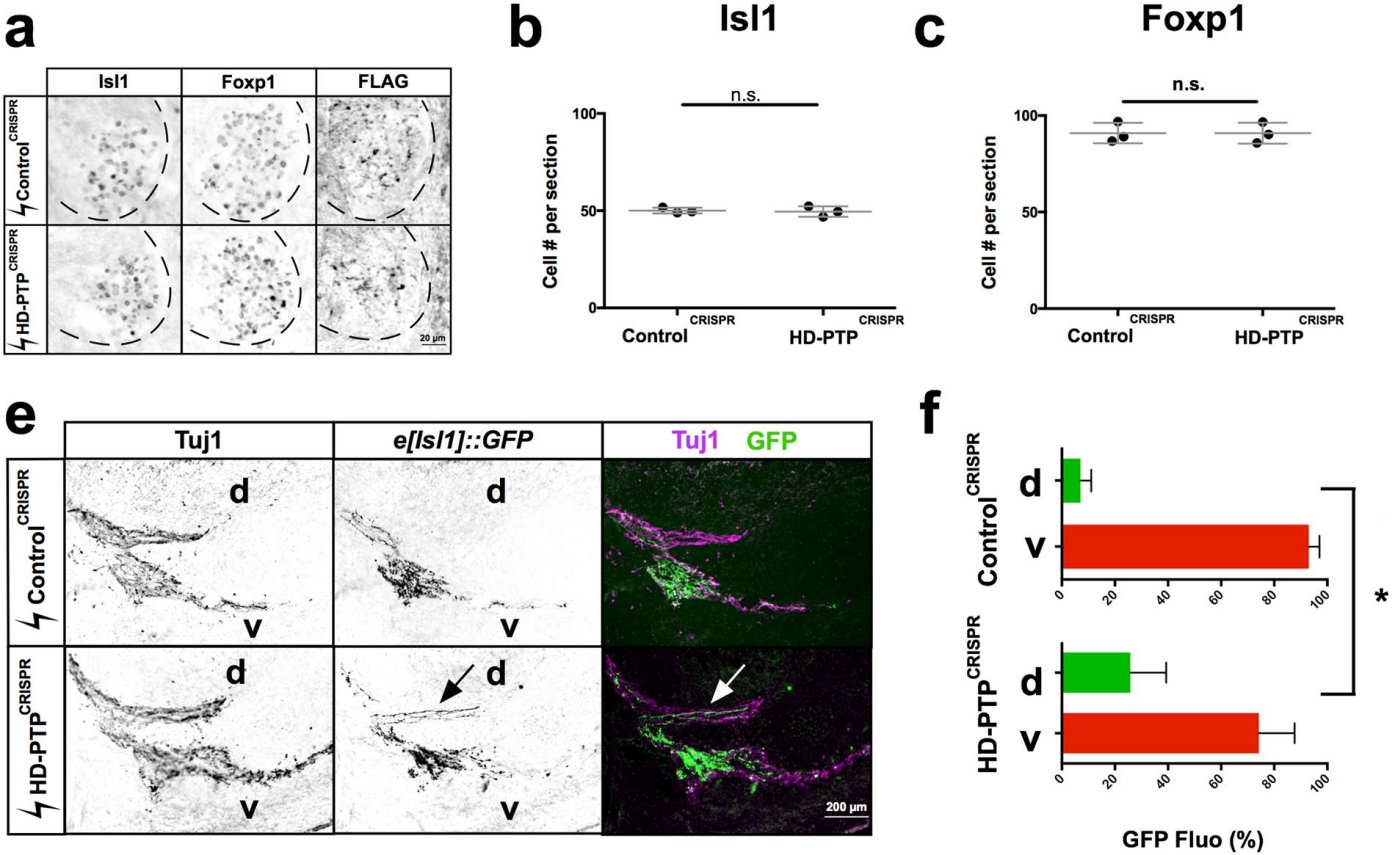
HD-PTP is required for ephrin-B:EphB-mediated spinal motor axon guidance *in vivo*. (**a**) Representative sections of Control^CRISPR^ and HD-PTP^CRISPR^ HH St. 25 spinal cords showing expression of Isl1, Foxp1 and FLAG, the Cas9 expression marker, demonstrating efficient electroporation of motor neurons. (**b**) Quantification of Isl1^+^ medial LMC neurons in Control^CRISPR^ and HD-PTP^CRISPR^ embryos. Their numbers are not significantly different between the two populations of embryos (n = 3, 10 sections/n; Student’s t-test). (**c**) Quantification of Foxp1^+^ LMC motor neurons in Control^CRISPR^ and HD-PTP^CRISPR^ embryos. Their numbers do not differ between the two conditions (n = 3, 10 sections/n; Student’s t-test). (**d**) Representative images of the limb nerve in Control^CRISPR^ and HD-PTP^CRISPR^ HH St. 25 embryos, stained with anti-Tuj1 antibodies to reveal limb nerves and *e[Isl1]::GFP* in medial LMC axons. Medial axons aberrantly innervate the dorsal mesenchyme in HD-PTP^CRISPR^ embryos. (**e**) Quantification of *e[Isl1]::GFP* expression in dorsal vs. ventral nerves. Control^CRISPR^ embryos contain ~93% of GFP in the ventral nerve and ~7% in the dorsal nerve. HD-PTP^CRISPR^ embryos contain ~74% of GFP in the ventral nerve and ~26% in the dorsal nerve, demonstrating that disruption of HD-PTP *in vivo* impairs the fidelity of medial LMC axons (*n* = 5 embryos, 10–20 sections/*n*; *p* = 0.0149; Student’s *t-*test between GFP signal % in dorsal Control^CRISPR^ vs. dorsal HD-PTP^CRISPR^). Values are plotted as mean ± SD. All values can be found in Supplementary Table S4. d: dorsal; v- ventral; *p < 0.05; n.s.: not significant. Inverted grayscale fluorescent images and dual colour images.

Having established a function of HD-PTP in ephrin-B2:EphB2 signalling and its perdurance, and its requirement for normal cellular responses to eB2, we assessed the role of HD-PTP *in vivo*. We hypothesised that HD-PTP loss in developing LMC neurons would attenuate growth cone repulsion from dorsal limb-expressed ephrin-B2, resulting in medial LMC axons entering dorsal limb nerves, as in mice with a loss of ephrin-B:EphB signalling^5^. Thus, we co-electroporated HH st. 18 chick spinal cords with untagged HD-PTP^CRISPR^ or Control^CRISPR^ expression plasmids, and the medial LMC-specific axonal marker plasmid *e[Isl1]::GFP* (Fig. 8d)^55^. We achieved only modest levels of co-expression (Supplementary Fig. S5), likely due to the low concentration of the plasmids in the DNA mix, a necessary limitation when electroporating four plasmids. Nevertheless, sufficient numbers of axons were labelled to allow for analysis. Loss of HD-PTP function did not result in abnormal LMC neuron specification or survival at HH st. 25, when LMC axons enter the dorsal and ventral hindlimb nerves^56^ (Fig. 8a–c). At this stage, in Control^CRISPR^ + *e[Isl1]::GFP* embryos, 7% of axonal GFP signal was found in dorsal limb nerves and 93% in ventral limb nerves, similar to the incidence of medial LMC labelling by retrograde axonal tracer injected into dorsal and ventral limb muscles^5^. In contrast, in HD-PTP^CRISPR^ + *e[Isl1]::GFP* embryos, ~25% of axonal GFP signals were found in dorsal limb nerves and ~75% of them were found in ventral limb nerves, a significant difference from controls (Fig. 8d,e; *n* = 5; *p* = 0.0149), demonstrating that HD-PTP is required for the normal guidance of medial LMC motor axons *in vivo*.

## Discussion

Our proteomics experiments identify a number of potential novel effectors of Eph signalling, and demonstrate that one such protein is the ESCRT adaptor HD-PTP. Its association with EphB2 is induced by ephrin-B2 binding, and its function is required for repulsive responses to ephrin-B2 in cultured cells and motor neuron growth cones, as well as the normal guidance of ephrin-B-responsive spinal motor axons *in vivo*. HD-PTP is required for EphB2 clustering, and protects activated receptor from ligand-induced lysosomal degradation. Here, we discuss these findings in the context of general principles of ephrin:Eph signalling, and consider the role of ESCRT proteins in axon guidance.

We used BioID and mass spectrometry to probe the EphB2-associated protein landscape during forward signalling. In general, biological process and pathway analysis of our results align with previously defined functions of ephrin:Eph signalling in neurodevelopment and cytoskeletal organisation, through its action at cell-cell junctions, cell periphery and membrane, and GTPase regulation^1^. Our list of EphB2-proximal proteins includes some known EphB2 effectors such as NCK1, NCK2, CRK, and YES, further arguing that our ligand stimulation strategy identifies biologically-relevant protein-protein interactions^14,57,58^. Our data also confirmed the association of EphB2 with the Unc5 class of netrin receptors, which results in synergistic EphB signalling^41^. Indeed, given that ephrin-evoked Eph signalling occurs on the scale of minutes and that our biotinylation of EphB2-proximal proteins proceeded on the scale of hours, our results suggest that the BioID methodology is able to capture even relatively short-lived protein-protein interactions.

Together with a recent BioID data set of EphA2-proximal proteins^59^, our results point to several biological processes in ephrin:Eph signalling that lack a detailed mechanistic description, including endosomal transport and cell division and differentiation. Endocytosis plays a prominent role in Eph signalling^60^ and, while primary ESCRT components did not appear in our list, we did find ESCRT-associated adaptors such as HD-PTP and the RUN and FYVE domain-containing protein 1 (RUFY1), which was recently reported to function, together with HD-PTP, in EGFR trafficking^61^. Few direct ephrin:Eph effectors of cell differentiation and proliferation have been identified^62^, but some studies have implicated the PI3-Kinase and Abl-cyclinD1 pathways and, more recently, histone methylation via Akt^63,64^. Our proteomic identification of EphB2 proximal proteins such as Abl2 and Pik3r1 confirmed these links, but also suggested a novel intermediary in Notch2, which would allow Eph receptors to intersect with a transcriptional response pathway controlling a multitude of developmental and homeostatic processes^65^.

Our experiments show that HD-PTP can form an ephrin-B2-driven complex with EphB2 and plays a critical and early role in Eph signalling: cells and growth cones with even a partial loss of HD-PTP exhibit a marked disruption of collapse responses to ephrin-B, apparently because of decreased Eph receptor clustering, phosphorylation, and activation of Src family kinases. Among these, Eph receptor clustering is the most upstream event following ephrin-B2 binding, and a defect at either of these steps could explain reduced downstream phosphorylation. Since HD-PTP gain- or loss-of-function does not affect EphB2 abundance, surface localisation, or ligand binding, the simplest explanation of these effects would place HD-PTP at the ephrin-B2:EphB2 clustering step of signalling. Clustering depends on the extracellularly-located ligand-binding and the cysteine-rich domains^6,7,66^, but is also modulated by the intracellular PDZ and SAM domains, whose deletion enhances ephrin-induced Eph clustering and signalling in cultured cells^9^. Although without extensive biochemical analysis we are unable to establish the molecular mechanisms of HD-PTP’s role in receptor clustering, this study is the first report identifying an intracellular protein whose loss has a profound impact on this early step of Eph receptor activation. In line with HD-PTP’s role in ESCRT pathway progression, it is possible that HD-PTP’s loss affects indirectly the expression or subcellular localisation of a protein required for the early steps of Eph signalling. Such an indirect effect would have to be specific to the Eph signalling pathway, given that semaphorin-mediated responses are normal under HD-PTP loss-of-function conditions. Because of its ability to complex with EphB2, we favour the hypothesis that HD-PTP is participating in receptor clustering directly. Either way, our data argue that HD-PTP is an important molecular handle on the mechanisms regulating one of the earliest steps of Eph signalling.

**Figure 9:**
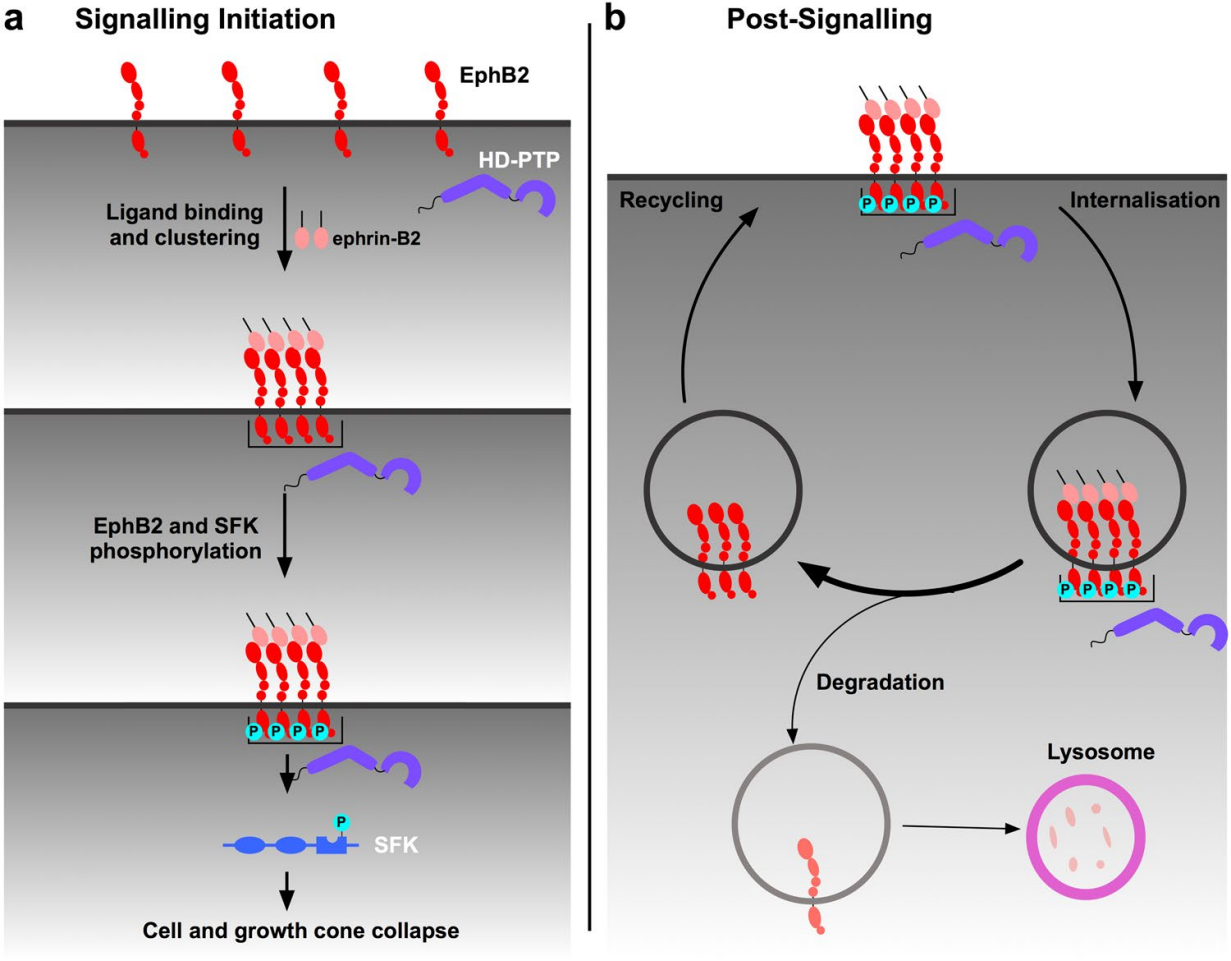
A model of the dual role of HD-PTP in EphB signalling. (**a**) Under basal conditions, EphB2 receptors exist at the membrane as unclustered molecules and HD-PTP does not associate with them. During signalling initiation, HD-PTP forms a molecular complex with ligand-activated EphB2, and promotes receptor multimerisation. HD-PTP loss results in dampened ligand-induced receptor phosphorylation, Src family kinase activation, and repulsive cellular responses *in vitro* and *in vivo*. (**b**) Ligand-bound EphB2 complexes are internalised in early endosomes, from where the receptor can be recycled back to the membrane or sorted to the endocytic pathway for lysosomal degradation. Our data shows that internalised EphB2 complexes are protected from lysosomal degradation in an HD-PTP-dependent manner. HD-PTP’s deubiquitylase-recruiting function may play a role in this process.

Our results suggest that HD-PTP may not only promote EphB2 clustering, but also inhibit its endocytic progression once internalised. Cells with a loss of HD-PTP display increased degradation of EphB2 receptor, most strikingly upon ligand stimulation. Our observation of diminished internalised EphB2 signals in ligand-stimulated cells with reduced HD-PTP compared to ligand-stimulated control cells is thus likely due to accelerated degradation, and not decreased receptor internalisation. This is surprising given HD-PTP’s known function in promoting the progression of activated cell surface receptors through the endocytic pathway, as shown for integrin α5β1, E-cadherin, and EGFR, where loss of HD-PTP results in endocytic accumulation and exacerbated signalling^24,25,67^. How are HD-PTP-depleted cells, which are unable to fully collapse in response to ephrin-B2, nonetheless able to accelerate EphB2 degradation upon ligand binding? One possibility is that ephrin-bound, but unclustered, receptor multimers may be degraded more efficiently compared to ligand-unbound monomers or dimers and, especially, compared to clustered and ligand-activated multimers. This protection of large, activated EphB2 clusters may be the result of their sequestration in specialised endosomes. Ligand-activated EGFR can be sequestered in this way, halting its endocytic progression and protecting it both from recycling and from degradation, but, intriguingly, this phenomenon is observed only in cells experiencing concurrent ephrin-A:EphA signalling^68^. This cross-talk between the active Eph signalling cascade and EGFR suggests the possibility that signal prolongation via HD-PTP is a general property of Eph receptors and that EGFR, by being simultaneously activated, piggybacks on Eph endosomal protection. Further insights come from the observation of reduced Wnt signalling due to the impaired function of its receptor in *Drosophila* wing imaginal disks lacking HD-PTP^69^. This study argues that HD-PTP recruits deubiquitylases to counterbalance the ubiquitylation of both Wnt receptor and the endosome-associated protein Hrs/HGS, which normally promotes the recycling of Wnt receptors destined for lysosomal degradation. In this context, HD-PTP loss results in increased ubiquitylation and lysosomal degradation of Hrs/HGS, leading to increased endocytic accumulation of ubiquitylated Wnt receptors and their decreased recycling. Thus, one explanation for the accelerated depletion of EphB2 observed in HD-PTP deficient cells following ephrin-B stimulation could be through a similar impact on deubiquitylation of endosomal proteins or Eph receptors themselves. Indeed, EphB receptors are ubiquitylated in response to ligand binding^19,70^. Therefore, an alternative model of HD-PTP function may involve its recruitment of deubiquitylases that counterbalance ephrin-B-induced EphB ubiquitylation, as well as promoting its recycling, since deubiquitylated Eph receptors are more likely to be recycled^20^. Together, our data suggest that HD-PTP has dual roles in the ephrin-B:EphB signalling cascade: early on, it is required for the initiation of signalling and, further downstream, it acts as a negative regulator of receptor degradation (Fig. 9).

Our *in vivo* experiments uncover an important role for HD-PTP in nervous system development, through its function in the formation of connections between spinal motor neurons and their limb muscle targets. HD-PTP-deficient spinal motor axons, normally destined for the ventral limb nerve, aberrantly enter the ephrin-B2-enriched dorsal limb mesenchyme. Based on the requirement of HD-PTP for ephrin-B2-induced motor neuron growth cone collapse *in vitro*, the *in vivo* phenotype is likely exerted through impaired ephrin-B:EphB signalling, although HD-PTP loss may also affect the response of spinal motor axon growth cones to other limb mesenchyme-derived signals important for motor axon guidance such as Netrin^41^ or Semaphorins^51^. There is no evidence of HD-PTP function in Netrin signalling, but the ESCRT-II complex has been implicated in controlling the expression of DCC, a Netrin receptor^71^. In the case of Semaphorins, cells and growth cones deficient in HD-PTP respond normally to Sema3F and Sema3A, arguing against HD-PTP’s involvement in Semaphorin-mediated motor axon guidance. However, a different adaptor protein, GIPC1, has recently been shown to be indispensable for Plexin-D1 responses to Sema3E by sorting activated receptor into particular endosomes containing signalling effectors^72^. Our experiments and the studies discussed here herald the emerging prominence of post-endocytic control of axon guidance receptor function: core ESCRT proteins could form a pervasive regulatory module enabling endocytic processing of receptors, while ESCRT accessory proteins like GIPC1 or HD-PTP may link such a module to specific axon guidance proteins to direct their spatiotemporal activities.

Our experiments linking ESCRT, a pervasive system controlling the fate of many transmembrane receptors, and Eph signalling, a rapid-action pathway underlying a wide variety of biological processes, bring many potential new insights into their understanding. For example, increased tumourigenesis caused by the loss of HD-PTP has been attributed to excessive surface receptor signalling^73^, but in light of our data, could also be a consequence of impaired anti-cancer activity of Eph signalling^3^. Our work could also bridge two disparate findings: mutations in an ESCRT component have been found in populations with familial Amyotrophic lateral sclerosis^74^ (ALS), and EphA activity is negatively correlated with prognosis in ALS patients^75^. The control of Eph signalling by ESCRT proteins could thus be an important new therapeutic avenue in the context of tumourigenesis and other disorders involving Eph signalling, such as neurodegeneration^76^.

## Methods

### Animals

All animal experiments were carried out in accordance with the Canadian Council on Animal Care guidelines and approved by the IRCM Animal Care Committee and the McGill University Animal Care Committee. Fertilised chicken eggs (FERME GMS, Saint-Liboire, QC, Canada) were incubated at 38 °C and staged according to Hamburger and Hamilton^42^.

### BioID and MS data analysis

BioID experiments were performed as described elsewhere, with modifications^77^. Briefly, Control and EphB2-OE Flp-In T-REx HEK293 cells (Invitrogen) (all cell lines can be found in Supplementary Table S3) were cultured in 15 cm plates (Corning) and treated with 1 µg/mL of tetracycline (Sigma Aldrich) for 18 h. The following day, the medium was removed and cells were incubated in serum-free medium for 6 h in the presence of 50 µM biotin (Sigma Aldrich) and pre-clustered ligand (Fc or eB2-Fc, 1.5 μg/mL, R&D Systems) or media. After 6 h of biotin and ligand treatment, the medium was removed, cells were scraped from the plates, washed 3 times with cold phosphate-buffered saline (PBS) in 15 mL tubes and cell pellets were stored at −80 °C. Cells were lysed in 1.5 mL radioimmunoprecipitation assay (RIPA) buffer and 1 µL of benzonase (MilliporeSigma) was added to each sample to degrade nucleic acids. Lysates were sonicated for 30 seconds (s) at 30% amplitude, in 10 s bursts with 2 s rest in between. Lysates were then centrifuged for 30 min at maximum speed at 4 °C. 70 µL of pre-washed streptavidin beads (GE Healthcare) were incubated with the remaining lysate for 3 h at 4 °C. Samples were spun down for 1 min at 2000 rpm at 4 °C and the supernatant was removed. Beads were re-suspended in 1.5 mL RIPA buffer and washed 3 times with RIPA buffer. Beads were then re-suspended in 1 mL of 50 mM Ammonium Bicarbonate (ABC, Bio Basic), washed 3 times with ABC and re-suspended in 100 µL of ABC. 1 µg of trypsin (Sigma Aldrich) was added and samples were shaken at 37 °C for 16 h. The following day, samples were trypsin-digested for 2 h and spun down for 1 min at 2000 rpm at room temperature. Beads were washed 2 times in 100 µL of water and combined with the collected supernatant. Formic acid (Sigma Aldrich) was added to the supernatant for a final concentration of 5%. Samples were spun down for 10 min at maximum speed at room temperature, dried for 3 h at 30 °C (SpeedVac). Tryptic peptides were resuspended in 15 µL of 5% formic acid and stored at −80 °C.

Peptides were analysed by high-pressure liquid chromatography (HPLC) coupled to Orbitrap Velos Mass Spectrometer (Thermo Fisher Scientific) at the IRCM proteomics core facility. Peptide search, identification of proteins and mass spectrometry (MS) data analysis were carried out as described elsewhere^77^. The BioID-MS data was analysed using ProHits^78^. Briefly, RAW files were converted to.mzXML using Proteowizard^79^. Human RefSeq Version 57 and the iProphet tool integrated in ProHits^80^ were used for peptide search and identification. Significance Analysis of INTeractome (SAINT) file inputs generated in ProHits were analysed through ProHits-viz^32^ to generate dot plots and to calculate WD-scores.

### Protein network analysis, clustering and functional annotation

Protein network and clustering analyses were generated via Cytoscape^34^ as described elsewhere^81^, with modifications. The BioID-MS data analysed in SAINT were imported to Cytoscape. Reviewed UniProtKB entries of the preys identified in SAINT were submitted into the ‘Enter Search Conditions’ text box and the existing protein-protein interaction data was imported from IntAct database^82^. The BioID and public networks were merged by performing a union merge. Self-loops and duplicated edges were removed. MCL Cluster^35^ was used to visualise protein complexes and clusters. Functional annotation was performed using Gene Ontology (GO) terms. The known biological process or molecular function of prey proteins was analysed by using g:Profiler^33^. Reviewed UniProtKB entries of the preys analysed in SAINT were submitted in the Query field on g:Profiler and the −log_10_ of corrected *p* values were used for GO enrichment and KEGG analysis.

### Biochemistry

For the co-immunoprecipitation assays, Control and EphB2-OE HEK293 cells were transfected with an HD-PTP-HA expression plasmid in 10 cm dishes using Lipofectamine 3000 (Invitrogen), and one day later were incubated with DMEM (Gibco) supplemented with 0.05% foetal bovine serum (Gibco), 1% penicillin/streptomycin (100X, Gibco) and 1 µg/mL of tetracycline (Sigma Aldrich) for 18 h at 37 °C with 5% CO_2_. Control^shRNA^ and HD-PTP^shRNA^ HeLa cells (all cell lines can be found in Supplementary Table S3) were transfected with an EphB2-FLAG expression plasmid (gift from Dr. Matthew Dalva) using 2 mM calcium phosphate and stimulated 48 h after transfection. Stimulation with pre-clustered ligands (clustered with anti-Fc antibody for 30 min at room temperature) was for 15 min (Fc and eB2, 1.5 μg/mL) or 5 min (Fc and eB2, 1.0 μg/mL) at 37 °C. After a wash with PBS, cells were lysed with 1 M MgCl_2_, 2 M Tris-HCl pH 7.5, 3 M NaCl, 1% CHAPS (Bio Basic), 0.5 M sodium fluoride, 100 mM sodium orthovanadate and cOmplete proteinase inhibitor (25X, Roche). Lysates were spun down at 14,000 rpm for 15 min at 4 °C then supernatant was transferred and rotated with anti-FLAG beads (Sigma Aldrich) for 3 h at 4 °C, washed 3 times with lysis buffer (same as above) and denatured with 6X Laemmli buffer (1:5).

For other biochemical assays, Control and EphB2 HeLa cells were incubated with DMEM supplemented with 0.05% foetal bovine serum, 1% penicillin/streptomycin and 1 µg/mL of tetracycline for 18 h at 37 °C with 5% CO_2_. Cells were lysed with 1 M Tris-HCl pH 8.0, 5 M NaCl, 1% NP-40 (Abcam), phosSTOP (Sigma Aldrich) and cOmplete proteinase inhibitor after a PBS wash. Lysates were spun down at 12,000 rpm for 5 min at 4 °C and denatured with 6X Laemmli buffer (1:5). Samples were run on 6–10% Bio-Tris polyacrylamide gels. Membranes (PVDF; Bio-Rad Laboratories) were activated with methanol (Sigma Aldrich) for 2 min and put in either 1% BSA (Bio Basic), 0.05% Tween (Sigma Aldrich) PBS or 5% milk blocking solution on a shaker for 45 min at room temperature and the following antibodies were applied: anti-GAPDH (1% BSA, 45 min at room temperature), anti-FLAG-HRP (1% BSA, 45 min at room temperature), anti-Streptavidin-HRP (1% BSA, 30 min at room temperature), anti-HA (5% milk, 1 h at room temperature), anti-ß-actin (5% milk, 1 h at room temperature), anti-pSrc-Y416 (1% BSA, overnight 4 °C), anti-Src (1% BSA, overnight 4 °C), and anti-HD-PTP (0.05% Tween PBS, overnight 4 °C). Information for all antibodies can be found in Supplementary Table S1. Membranes were activated with ECL (GE Healthcare) and revealed with film (GE Healthcare). Signal intensity and area of the immunoblot band was measured using ImageJ (NIH).

### Cell culture

Control and EphB2-OE HEK293 and HeLa cells were generated by transfecting Flp-In T-REx HEK293 and Flp-In T-REx HeLa cells with either FLAG or EphB2-BirA*-FLAG expression plasmids using Lipofectamine 3000. Transfected cells were selected with hygromycin (200 μg/mL, Invitrogen) for 15–16 days. Control^shRNA^ and HD-PTP^shRNA^ HeLa cells were generated by viral infection of either empty pLKO1 or HD-PTP shRNA pLKO1 (Sigma Aldrich). After infection, cells were selected by puromycin (1 μg/mL, Gibco) for 5–7 days and a western blot was performed to assess knock-down efficiency.

### HeLa cell collapse assay

Control and EphB2 HeLa cells were seeded at 20,000 cells per coverslip (VWR). After 24 hours, cells were incubated in DMEM supplemented with 0.05% foetal bovine serum, 1% penicillin/streptomycin and 1 µg/mL of tetracycline for 18 h at 37 °C with 5% CO_2_ and stimulated with pre-clustered eB2 or Fc the next day. Control^shRNA^ and HD-PTP^shRNA^ HeLa cells were cultured in DMEM supplemented with 10% foetal bovine serum, 1% penicillin/streptomycin and puromycin (1 μg/mL) at 37 °C with 5% CO_2_. These cells were transfected in 6-well plates (Sarstedt) using Lipofectamine 3000, seeded at 20,000 cells per coverslip and stimulated 48 h after transfection and 24 h after being seeded.

### Chick *in ovo* electroporation and CRISPR guides

Chicken spinal cord electroporation of expression plasmids was performed at HH st. 18/19 as described^48^. Three guide RNAs were designed against the HD-PTP *Gallus gallus* genomic sequence using CHOPCHOP^83^ and were verified for specificity using the NCBI BLAST tool^84^. The pX330 plasmid (#42230 obtained from Addgene) was modified by subcloning T2A-EGFP cassette downstream and in frame to Cas9, producing pX3361. Guide RNA oligos (Synthego) were synthetically made and each was cloned individually in the pX3361 plasmid. Guide RNA sequences are available upon request. For growth cone collapse experiments, embryos were electroporated with a DNA mix containing 1.5 μg/μL of each of the three guide-containing pX3361 plasmids. For the *in vivo* LMC axon guidance experiment, embryos were electroporated with a DNA mix containing 1 μg/μL of each of the three guide-containing pX3361 plasmids and 2 μg/μL of *e[Isl1]::GFP* plasmid.

### *In situ* mRNA localisation and immunohistochemistry

*In situ* mRNA detection and immunofluorescence were performed as described^49^ or using standard methods. Probe sequences are available upon request.

For non-permeabilised assays on LMC growth cones, tissue was exposed to ligands for 15 min and placed on ice, and a 5 min blocking step was performed by replacing half the media with PBS containing 2% BSA (final, 1% on tissue) and incubating at 4 °C. Half of the media was then replaced with motor neuron media (Neurobasal media (Gibco) supplemented with B-27 (1:50, Gibco), 0.5 mM L-Glutamate (Sigma Aldrich), 25 mM L-Glutamine (Gibco), and 1% penicillin-streptomycin containing primary antibodies against EphB2 (1 in 1000) and EEA1 (1 in 500) as control, and incubated for 30 min at 4 °C (all antibody details can be found in Supplementary Table S1). Tissue was then fixed with a mixture of 1/5 30% sucrose (Bio Basic) and 4/5 4% PFA (Sigma Aldrich) for 15 min at 4 °C. Three washes with PBS were followed by adding secondary antibodies (final, 1 in 1000 in PBS) for 1 h at 4 °C. Finally, three quick washes with PBS were followed by mounting in Mowiol (Sigma Aldrich). For the permeabilised control, fixation occurred after ligand incubation and before primary antibody staining, primaries were added in media with added Triton X-100 (0.3%, Sigma Aldrich), and secondary antibodies in PBS with added Triton X-100 (0.3%). Otherwise, all concentrations, incubation times, and temperatures were identical.

To quantify available surface ephrin-B2 receptor amounts in growth cones electroporated with HD-PTP CRISPR or control constructs (Fig. 6e,f) we cultured chick LMC growth cones from electroporated embryos, and bath-applied recombinant ephrin-B2-Fc (without previous clustering with anti-Fc antibodies) for 2 minutes before fixing. We then performed an anti-Fc antibody stain to detect the bound ephrin-B2-Fc. This was the only experiment in which we did not pre-cluster the recombinant ephrin-B2.

For non-permeabilised assays on HeLa cells, cells were exposed to ligands for 5 min and placed on ice immediately. Blocking was done by replacing half the media with PBS containing 2% BSA (final, 1% on tissue) for 5 min at 4 °C, whereupon half the media was replaced with DMEM supplemented with 0.05% foetal bovine serum, 1% penicillin/streptomycin, and containing primary antibodies against EphB2 (1 in 1000) and EEA1 (1 in 500) as control, and incubated for 30 min at 4 °C. Secondary staining was performed as in growth cones.

### Internalisation assay

HeLa cells or dissociated LMC motor neurons were cultured on coverslips. Before ligand stimulation, media was changed to media containing primary antibodies (1:1000 anti-EphB2), and tissue was incubated for 1 h at 4 °C. Coverslips were washed with warm media twice and the coverslips were treated with either pre-clustered Fc or ephrin-B2-Fc at 37 °C (10 min for HeLa cells, 20 min for neurons). After ligand incubation, all media was removed and replaced with an acid wash solution (1 M NaCl, 0.5 M Acetic Acid pH 2.2–2.7 (Sigma Aldrich)) for 6 min at room temperature. Cells and neurons were fixed with 4% PFA and stained for HD-PTP as well as with secondary antibodies to visualise internalised EphB2.

### Motor neuron culture

HH st. 25 chick embryos were harvested and dissected to isolate the motor column of the spinal cord. Tissue was dissociated with 0.25% trypsin (Life Technologies) in Ca^2+^/Mg^2+^ Hanks’s Solution (Invitrogen) deactivated by 1 M MgSO_4_ (Invitrogen) and 12500 U/mL DNAse (Worthington Industries). Cells were spun down at 1000 rpm for 5 min at room temperature, and resuspended in Neurobasal media supplemented with 1% foetal bovine serum, 0.01% Glutamax (Invitrogen), and 0.01% penicillin/streptomycin then titrated. 20,000 cells were seeded onto laminin-coated (20 μg/mL; Invitrogen) coverslips and incubated at 37 °C with 5% CO_2_. Cells were stimulated with pre-clustered eB2 or Fc one day after being seeded.

### Microscopy and image quantification

High magnification images were taken using ZEN 2010 on a Zeiss LSM 700 confocal microscope. Lower magnification pictures were taken using LasX on a Leica DFC 488 light microscope. *In situ* hybridisation images were taking using OsteoMeasure on a Leica DM 4000 light microscope. Axon projection, mean intensity of signal, cell area, motor neuron numbers of limb sections were quantified using ImageJ (NIH) and methods previously described^55^.

### PCR

Chick HH st. 25 spinal cords were digested with 100 μg/mL proteinase K (Thermo Fisher) in SDS buffer (100 mM Tris pH 8.5, 5 mM EDTA, 200 mM NaCl, 0.2% SDS) at 55 °C for 3 h. The extracted DNA was precipitated by isopropanol, washed with 70% ethanol, and re-suspended in ddH_2_O. PCR amplification of HD-PTP genomic locus in control or HD-PTP^CRISPR^-electroporated tissue was performed using Qiagen Master Mix and the following primers: forward outside primer (tttggggcagacagacatct), reverse outside primer (tatctttcgcacccctgctc). Nested PCR was performed with 1 μL of the previous PCR reaction product, with Qiagen Master Mix and the following primers: forward inside (agaaaggcacctgctccca) primer, reverse inside primer (ttccagtcacacagcagctg). PCR products were then visualised on a 1% agarose gel after electrophoresis.

### Pulse chase

Control^shRNA^ and HD-PTP^shRNA^ HeLa cells were transfected in 10 cm dishes (as above) with an EphB2-FLAG expression plasmid (gift from Dr. Matthew Dalva) using Lipofectamine 3000. After 24 h, 80,000 cells were seeded into 24 well plates and stimulated with pre-clustered eB2 or Fc 24 h later. Cells were pulsed with 10 μg/mL cycloheximide and 1 μg/mL of pre-clustered ligand (Fc or eB2, 1.0 μg/mL). Samples were collected at various time points and total EphB2 protein quantity was analysed by immunoblotting. For lysosomal inhibition, 10 mM NH_4_Cl was added to the cycloheximide and ligand mixture.

### Statistical analysis

Data from the experimental replicates were evaluated using Prism (GraphPad Software). Means of individual experiments were compared and underwent various statistics. For 3 or more conditions, one-way ANOVA was used, followed, if necessary, by Student’s *t-*tests corrected for multiple comparisons. For comparing 2 conditions with less than four replicates, we assumed normal distributions and analysed them with Student’s *t-*tests. For the growth cone collapse assay, which entailed categorical analysis, Fisher’s exact test was used. The threshold for statistical significance was set at 0.05.

## Supplementary information

Supplementary Information

## Data Availability

The BioID mass spectrometry data generated and analysed during this study are available on MassIVE listed under MSV000083410.
