# Supplementary material for: The endosomal sorting adaptor HD-PTP is required for ephrin-B:EphB signalling in cellular collapse and spinal motor axon guidance
Source: Sci Rep. 2019 Aug 16;9:11945. doi: 10.1038/s41598-019-48421-9 (PMC6697728; doi:10.1038/s41598-019-48421-9)
Supplement: Supplementary file 1 — Supplementary Information [file 41598_2019_48421_MOESM1_ESM.pdf]

Supplementary Materials

The endosomal sorting adaptor HD-PTP is required for ephrin-B:EphB signalling in cellular collapse and spinal motor axon guidance.

Sylvie Lahaie[1,2]†, Daniel Morales[1,2]†^, Halil Bagci[1,3], Noumeira Hamoud[1], Charles-Etienne Castonguay[1], Jalal M. Kazan[4,5], Guillaume Desrochers[4,5], Avihu Klar[6], Anne-Claude Gingras[7,8], Arnim Pause[4,5], Jean-François Côté[1,3,9,10] and Artur Kania[1,2,3,11]*

[1]Institut de recherches cliniques de Montréal (IRCM), Montréal, QC, H2W 1R7, Canada

[2]Integrated Program in Neuroscience, McGill University, Montréal, QC, H3A 2B4, Canada

[3]Department of Anatomy and Cell Biology, McGill University, Montréal, QC, H3A 0C7, Canada

[4]Goodman Cancer Research Centre, McGill University, Montréal, QC, H3A 1A3, Canada

[5]Department of Biochemistry, McGill University, Montréal, QC, H3G 1Y6, Canada

[6]Department of Medical Neurobiology, IMRIC, Hebrew University-Hadassah Medical School, Jerusalem, 91120, Israel

[7]Lunenfeld-Tanenbaum Research Institute, Mount Sinai Hospital, Toronto, ON, M5G 1X5, Canada

[8]Department of Molecular Genetics, University of Toronto, Toronto, ON, M5S 1A8, Canada

[9]Programmes de Biologie Moléculaire, Département de Médecine, Université de Montréal, Montréal, QC, H3T 1J4, Canada

[10]Département de Biochimie, Université de Montréal, Montréal, QC, H3C 3J7, Canada

[11]Department of Biology and Division of Experimental Medicine, McGill University, Montréal, QC, H3A 2B2, Canada

[*] Correspondence: artur.kania@ircm.qc.ca

† These authors contributed equally to this work.

^ Current address: Neuroengineering Laboratory, Brain Mind Institute, École polytechnique fédérale de Lausanne (EPFL), Station 19, CH-1015, Lausanne, Switzerland

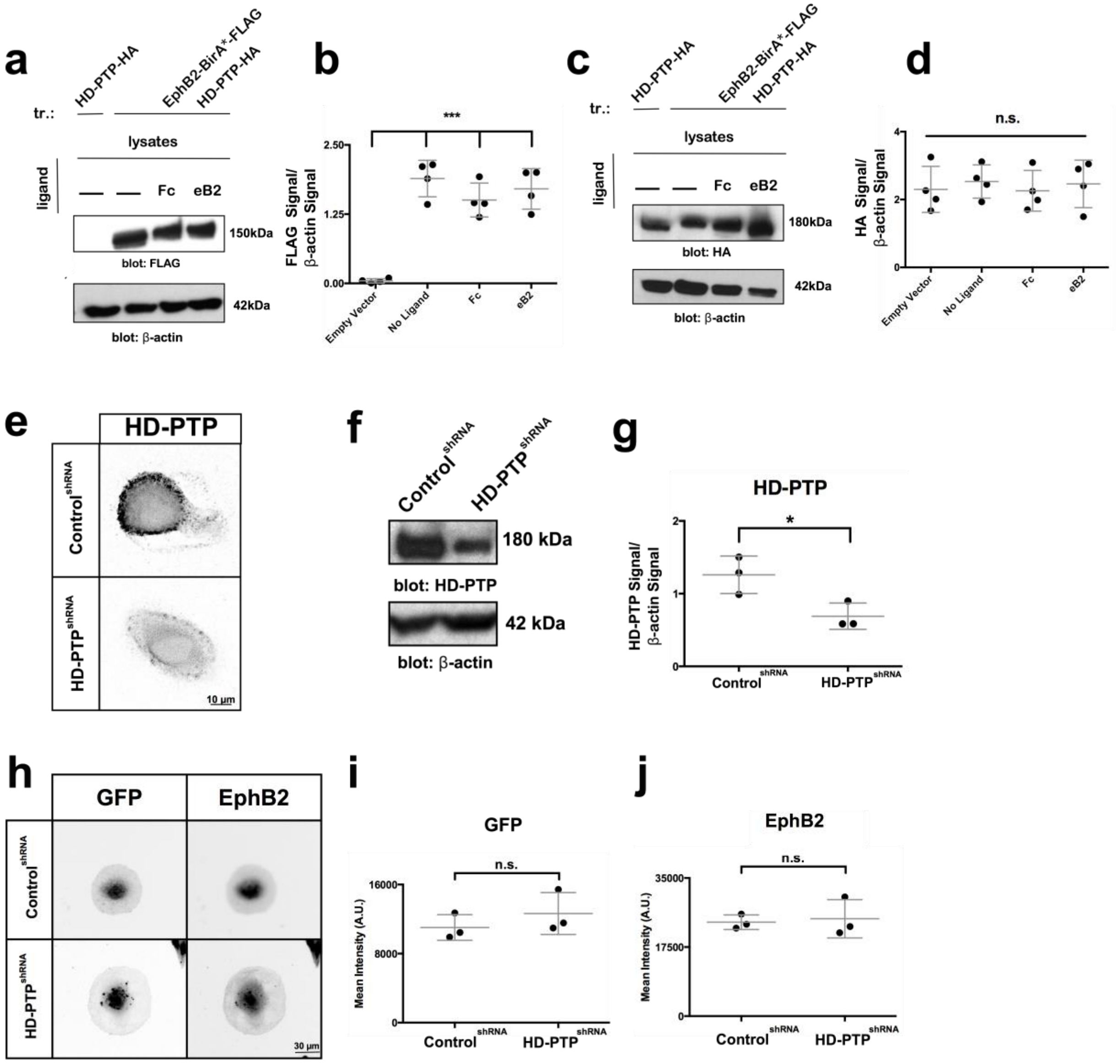

Supplementary Figure S1 related to Figure 2 and Figure 3. Co-IP total cell lysate quantification and HD-PTP shRNA HeLa cell characterisation. (**a**) Representative Western blot of FLAG and β-actin in lysates of HEK293 cells transfected with HD-PTP-HA and expressing either EphB2-BirA*-FLAG or FLAG alone. (**b**) Quantification of FLAG signal normalised to β-actin ($p < 0.001$) ($n = 4$, one-way ANOVA followed by Student's t-tests corrected for multiple comparisons). (**c**) Representative Western blot of HA and β-actin in lysates of HEK293 cells transfected with HD-PTP-HA and expressing either EphB2-BirA*-FLAG or FLAG alone. (**d**) Quantification of HA signal normalised to β-actin ($p= 0.912$) ($n = 4$, one-way ANOVA followed by Student's t-tests corrected for multiple comparisons). (**e**) Representative images of Control^{shRNA} and HD-PTP^{shRNA} HeLa cells stained with anti-HD-PTP (**f**) Representative Western blot of HD-PTP and β-actin in lysates of HeLa cells stably expressing Control^{shRNA} and HD-PTP^{shRNA}. (**g**) Quantification of HD-PTP signal normalised to β-actin (p=

0.0492) (*n* = 3, Student's t-tests). (**h**) Representative inverted grayscale fluorescent images of Control^{shRNA} and HD-PTP^{shRNA} HeLa cells transfected with EphB2-GFP plasmid, showing GFP and anti-EphB2 signals. (**i**) Quantification of GFP mean pixel intensity in Control^{shRNA} and HD-PTP^{shRNA} HeLa cells transfected with EphB2-GFP plasmid (*n* = 3, 60-80 cells/*n*; Student's *t*-test). (**j**) Quantification of EphB2 mean pixel intensity in Control^{shRNA} and HD-PTP^{shRNA} HeLa cells transfected with EphB2-GFP plasmid (*n* = 3, 60-80 cells/*n*; Student's *t*-test). Values are plotted as mean ± SD. All values can be found in Supplementary Table S4. Full Western blots can be found at the end of this document. kDa: kilodalton; A.U.: arbitrary units; eB2: ephrin-B2-Fc; * $p < 0.05$; n.s., not significant.

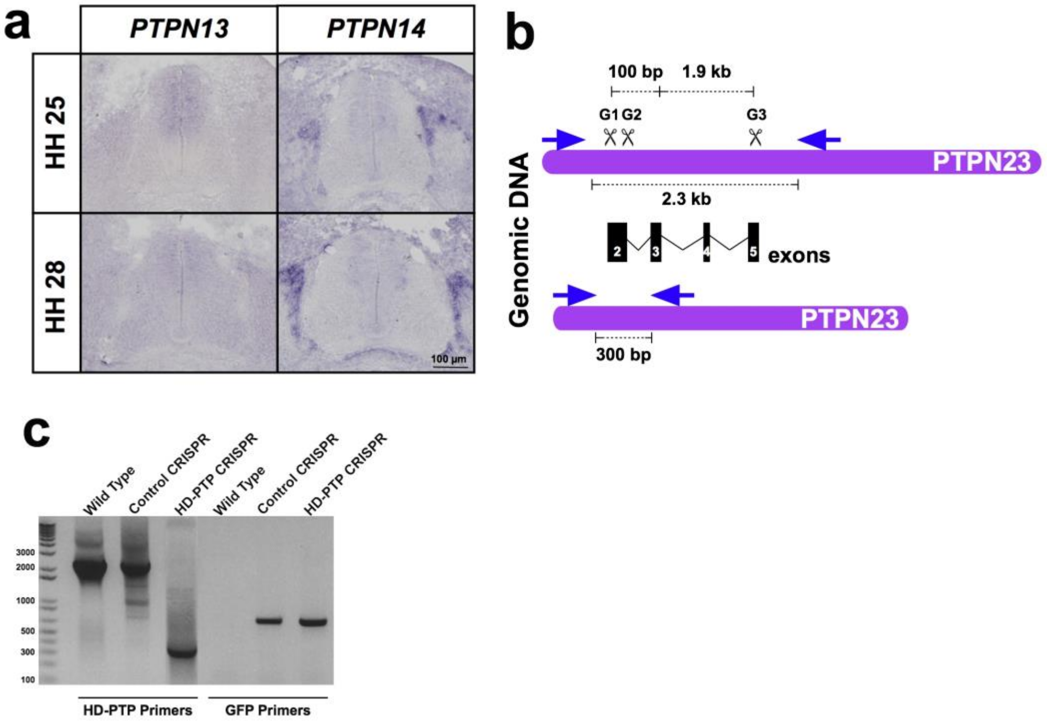

Supplementary Figure S2 related to Figure 4. Chick spinal cord CRISPR. (**a**) Representative images of chick embryonic spinal cord sections at HH st. 25 and HH st. 28 where *PTPN13* and *PTPN14* mRNA was detected using *in situ* hybridisation. Note absence from motor column, suggesting specificity in our *PTPN23* probe. (**b**) Schematic depicting the *PTPN23* genomic locus (chicken gene encoding HD-PTP), the location of CRISPR guides G1, G2 and G3 and PCR primers (arrows). The three guide RNAs produce deletions of exons 2-5. (**c**) Representative genomic PCR using the HD-PTP primers in (b) and GFP primers in DNA from a wild-type chick spinal cord, a Control^{CRISPR}-electroporated spinal cord, and an HD-PTP^{CRISPR}-electroporated spinal cord. HD-PTP primers show a full-length 2300 bp band in wild-type spinal cord and Control^{CRISPR} spinal cord, and a cleaved 300 bp band in the HD-PTP^{CRISPR} spinal cord. GFP primers show no band in wild-type spinal cords, and a 750 bp band in both Control^{CRISPR} and HD-PTP^{CRISPR} spinal cords (n = 3). Full Western blot can be found at the end of this document. HH: Hamburger-Hamilton stage; G: guide RNA; bp: base pairs; kb: kilobase.

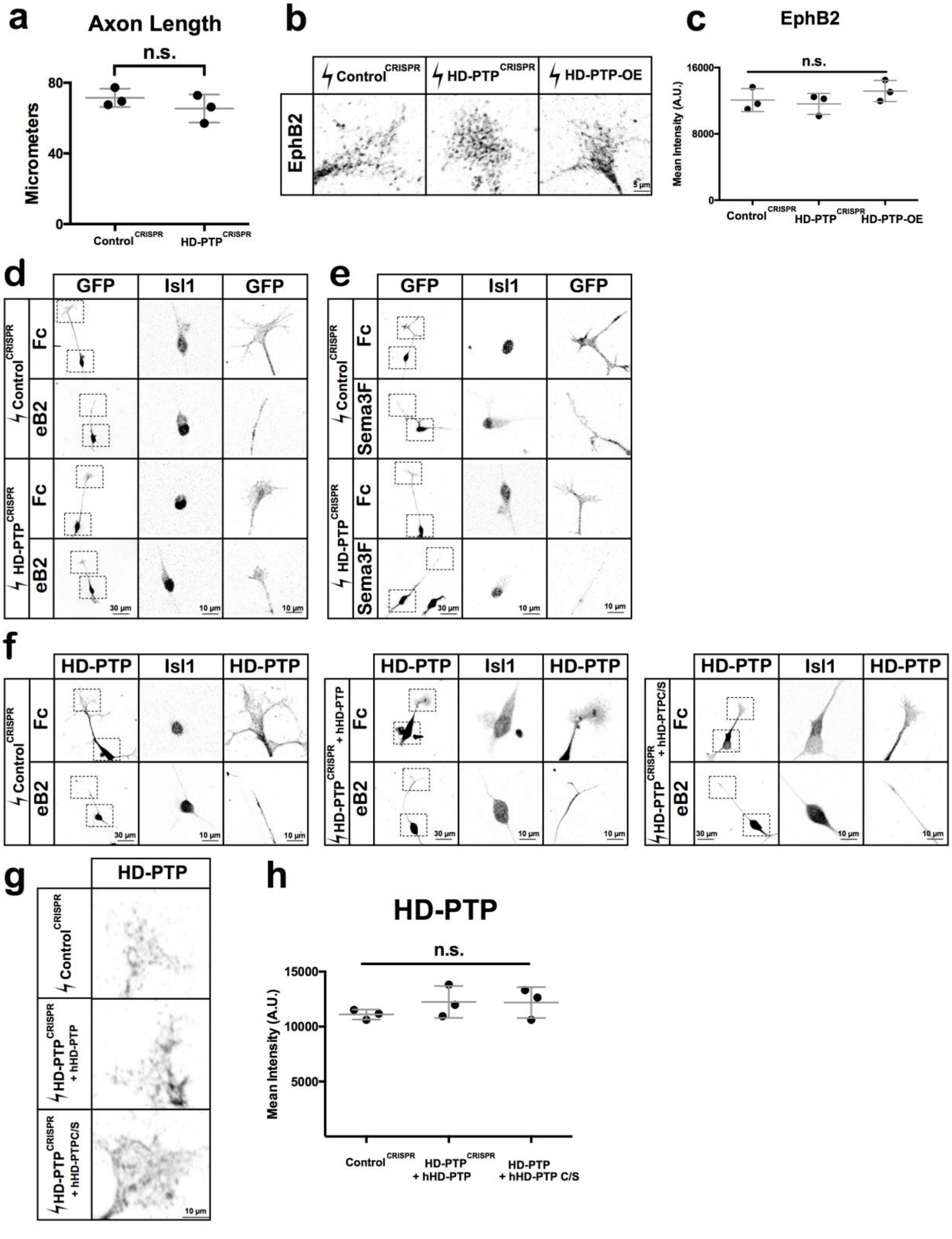

Supplementary Figure S3 related to Figure 5. Medial LMC growth cones require HD-PTP for ephrin-B2-induced collapse. (**a**) Quantification of Control^{CRISPR} and HD-PTP^{CRISPR}-

electroporated LMC motor neuron axon length *in vitro* (n = 3, 30-50 axons/n; Student's t-test). (**b**) Representative images of growth cones of LMC neurons electroporated either with Control^{CRISPR}, HD-PTP^{CRISPR} or hHD-PTP-FLAG plasmids, stained with anti-EphB2 antibody. (**c**) Quantification of EphB2 mean pixel intensity in LMC growth cones electroporated with Control^{CRISPR}, HD-PTP^{CRISPR} or hHD-PTP-FLAG (n = 3, 10-12 growth cones/n; Student's t-test). EphB2 levels are not affected by depleting HD-PTP. (**d**) Representative images of GFP^{+} neurons from dissociated Control^{CRISPR}- or HD-PTP^{CRISPR}-electroporated motor neurons, incubated with eB2 or Fc and stained with anti-GFP and anti-Isl1 antibodies. Insets show medial LMC Isl1-expressing cell bodies and growth cones. (**e**) Representative images of GFP^{+} neurons from dissociated Control^{CRISPR}- or HD-PTP^{CRISPR}-electroporated motor neurons, incubated with Sema3F or Fc and stained with anti-GFP and anti-Isl1 antibodies. Insets show medial LMC Isl1-expressing cell bodies and growth cones. (**f**) Representative images of rescue experiments with dissociated motor neurons electroporated with Control^{CRISPR} plasmid or HD-PTP^{CRISPR} co-electroporated with hHD-PTP or hHD-PTP C/S plasmid, incubated 30 min with 10 µg/mL eB2 or Fc and stained with anti-HD-PTP and anti-Isl1 antibodies. Insets show medial LMC Isl1-expressing cell bodies and growth cones. (**g**) Representative inverted grayscale fluorescent images of Control^{CRISPR}, HD-PTP^{CRISPR} + hHD-PTP, and HD-PTP^{CRISPR} + hHD-PTP C/S LMCm growth cones stained with the anti-HD-PTP antibody. (**h**) Quantification of HD-PTP mean pixel intensity of Control^{CRISPR}, HD-PTP^{CRISPR} + hHD-PTP, and HD-PTP^{CRISPR} + hHD-PTP C/S LMCm growth cones (n = 3, 10-12 growth cones/n; one-way ANOVA). Values are plotted as mean ± SD. All values can be found in Supplementary Table S4. A.U.: arbitrary units; h: human; eB2: ephrin-B2-Fc; *** $p < 0.001$; n.s.: not significant. Inverted grayscale fluorescent images.

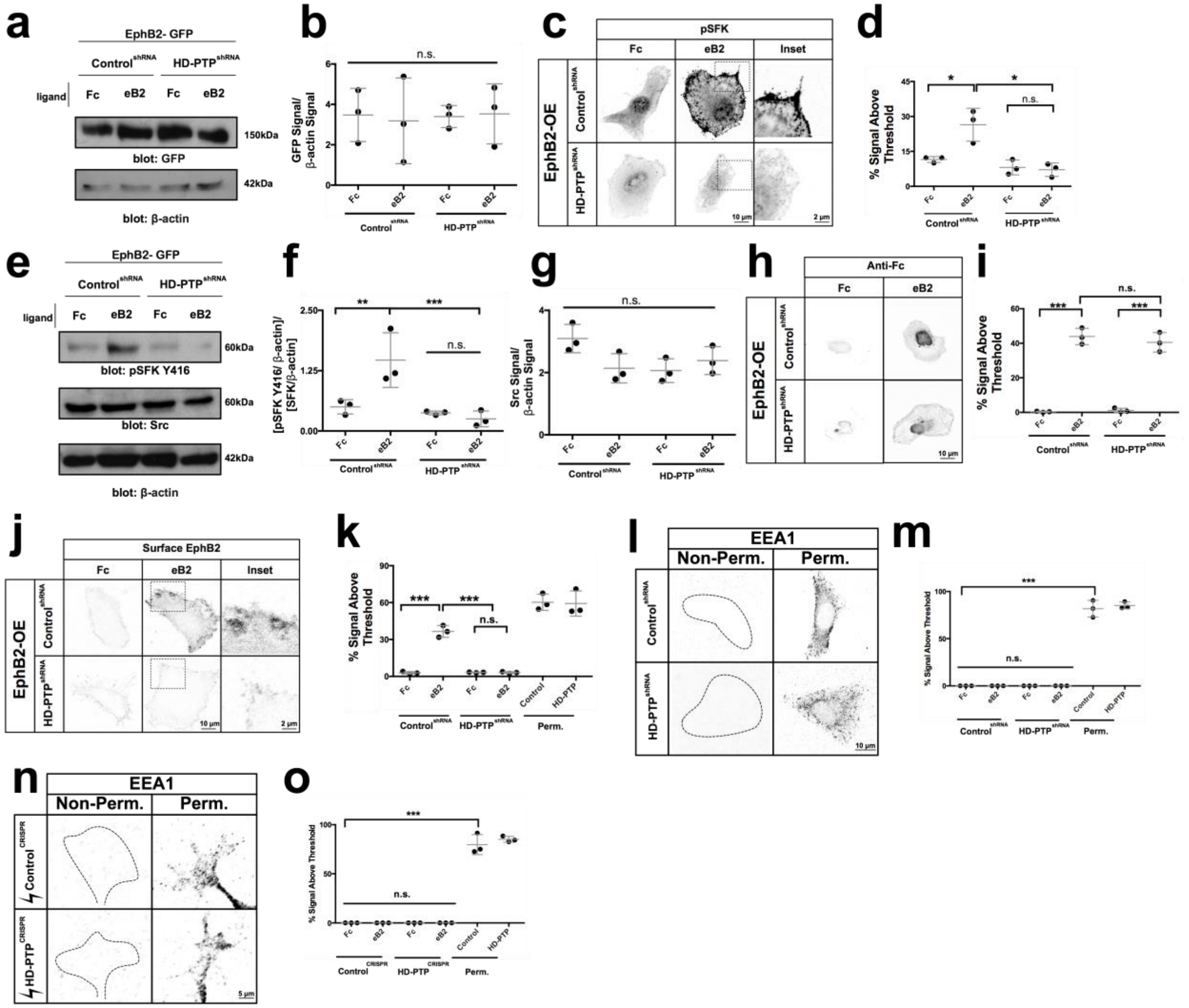

Supplementary Figure S4 related to Figure 6. HD-PTP is required for ephrin-B2-induced SFK activation, EphB2 phosphorylation, and EphB2 surface patching. (**a**) Representative Western blot using anti-GFP and anti-β-actin antibodies in Control^{shRNA} and HD-PTP^{shRNA} HeLa cell lysates, stimulated with 1 µg/mL eB2 or Fc for 5 min. The GFP band size corresponds to EphB2-GFP transfected into the HeLa cells. (**b**) Control^{shRNA} and HD-PTP^{shRNA} HeLa cell lysate EphB2-GFP expression normalised to β-actin is not significantly different (*p* = 0.8936) (*n* = 3; one-way ANOVA). (**c**) Representative images of Control^{shRNA} and HD-PTP^{shRNA} HeLa cells, incubated for 5 min with 1 µg/mL eB2 or Fc and stained with anti-phospho-Y418-SFK antibodies showing increased SFK activation following eB2 exposure. (**d**) Quantification of anti-phospho-Y418-SFK staining in Control^{shRNA} and HD-PTP^{shRNA} HeLa cells incubated for 5 min with 1 µg/mL eB2 or Fc. Control^{shRNA} showed an increase in phopho-Y418-SFK signal upon eB2 stimulation (*p* = 0.0227), yet HD-PTP^{shRNA} HeLa cells display no detectable increase in SFK phosphorylation (*p* = 0.7109) (*n* = 3, 10-12 cells/*n*; one-way ANOVA followed by corrected Student's *t*-tests). (**e**) Representative Western blot using anti-pSrc-Y416, anti-Src and anti-β-actin on Control^{shRNA} and HD-PTP^{shRNA} HeLa cells, stimulated with 1 µg/mL eB2 or Fc for 5 min. (**f**) Quantification of loading-normalised pSrc-Y416 signal over loading-normalised SFK signal shows ligand-induced activation of Src in Control^{shRNA} HeLa cells (*p* = 0.0044), but not in

HD-PTP^{shRNA} cells ($p = 0.8324$) ($n = 3$; one-way ANOVA followed by Student's *t*-tests). Normalisation to loading was performed due to signals from same lysate being developed on different membranes. (**g**) Quantification of Src signal over β-actin signal no difference in Src levels compared to Control^{shRNA} HeLa cells and HD-PTP^{shRNA} cells ($p = 0.7351$) ($n = 3$; one-way ANOVA followed by Student's *t*-tests). (**h**) Representative images of Control^{shRNA} and HD-PTP^{shRNA} HeLa cells, incubated for 2 min with unclustered 1 µg/mL eB2 or Fc and stained with anti-Fc antibodies showing increased anti-Fc staining following eB2 exposure. (**i**) Quantification of anti-Fc staining in Control^{shRNA} and HD-PTP^{shRNA} HeLa cells incubated for 2 min with 1 µg/mL of unclustered eB2 or Fc. Control^{shRNA} showed an increase in anti-Fc signal upon eB2 stimulation ($p < 0.0001$), and HD-PTP^{shRNA} HeLa cells display a detectable increase in anti-Fc signal after eB2 stimulation ($p < 0.0001$) ($n = 3$, 10-12 cells/n; one-way ANOVA followed by corrected Student's *t*-tests). (**j**) Representative images of Control^{shRNA} and HD-PTP^{shRNA} shRNA HeLa cells, incubated for 5 min with 1 µg/mL eB2 or Fc and immunostained for EphB2 using a non-permeabilising fixation conditions (see methods and Supplemental Fig. S5). EphB2 patching is visualised through increased signal intensity of surface EphB2 staining. (**k**) Quantification of surface EphB2 patching in Control^{shRNA} and HD-PTP^{shRNA} HeLa cells, incubated for 5 min with 1 µg/mL eB2 or Fc, measured by percentage of the cell area containing anti-EphB2 signal. In stark contrast to Control^{shRNA} cells ($p = 0.0003$), HD-PTP^{shRNA} HeLa cells failed to elicit EphB2 surface patching upon ligand binding ($p = 0.8609$) ($n = 3$, 10-12 cells/n; one-way ANOVA followed by corrected Student's *t*-tests). (**l**) Representative images of Control^{shRNA} and HD-PTP^{shRNA} HeLa cells, non-permeabilised vs. permeabilised, stained with the anti-EEA1 antibody. (**m**) EEA1 signal quantification in Control^{shRNA} and HD-PTP^{shRNA} HeLa cells transfected with EphB2-GFP plasmid and incubated for 5 min with 1 µg/mL eB2 or Fc. Controls for Fig. 8n ($n = 3$, 10-12 cells/n; one-way ANOVA). (**n**) Representative images of Control^{CRISPR} and HD-PTP^{CRISPR} LMC growth cones, non-permeabilised vs. permeabilised, stained with an anti-EEA1 antibody. (**o**) EEA1 signal quantification in Control^{CRISPR} and HD-PTP^{CRISPR} LMC growth cones that were incubated for 15 min with 10 µg/mL eB2 or Fc. Controls for Fig. 8p ($n = 3$, 10-12 growth cones/n; one-way ANOVA). Values are plotted as mean ± SD. All values can be found in Supplementary Table S4. Full Western blot can be found at the end of this document. kDa: kilodalton; eB2: ephrin-B2-Fc; Perm: permeabilised; *** $p < 0.001$; ** $p < 0.01$ * $p < 0.05$; n.s.: not significant. Inverted grayscale fluorescent images.

a

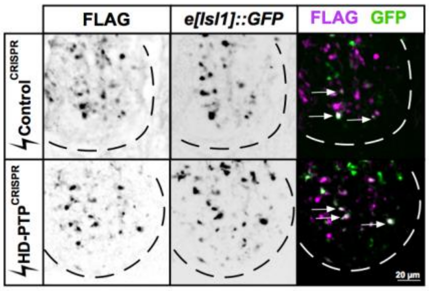

Supplementary Figure S5 related to Figure 8. CRISPR construct and *e[Isl1]::GFP* are co-expressed in LMCm region. (**a**) Representative images of the FLAG Cas9 expression marker and the medial LMC marker in *e[Isl1]::GFP* in Control^{CRISPR} and HD-PTP^{CRISPR} sections of HH St. 25 ventral spinal cords.

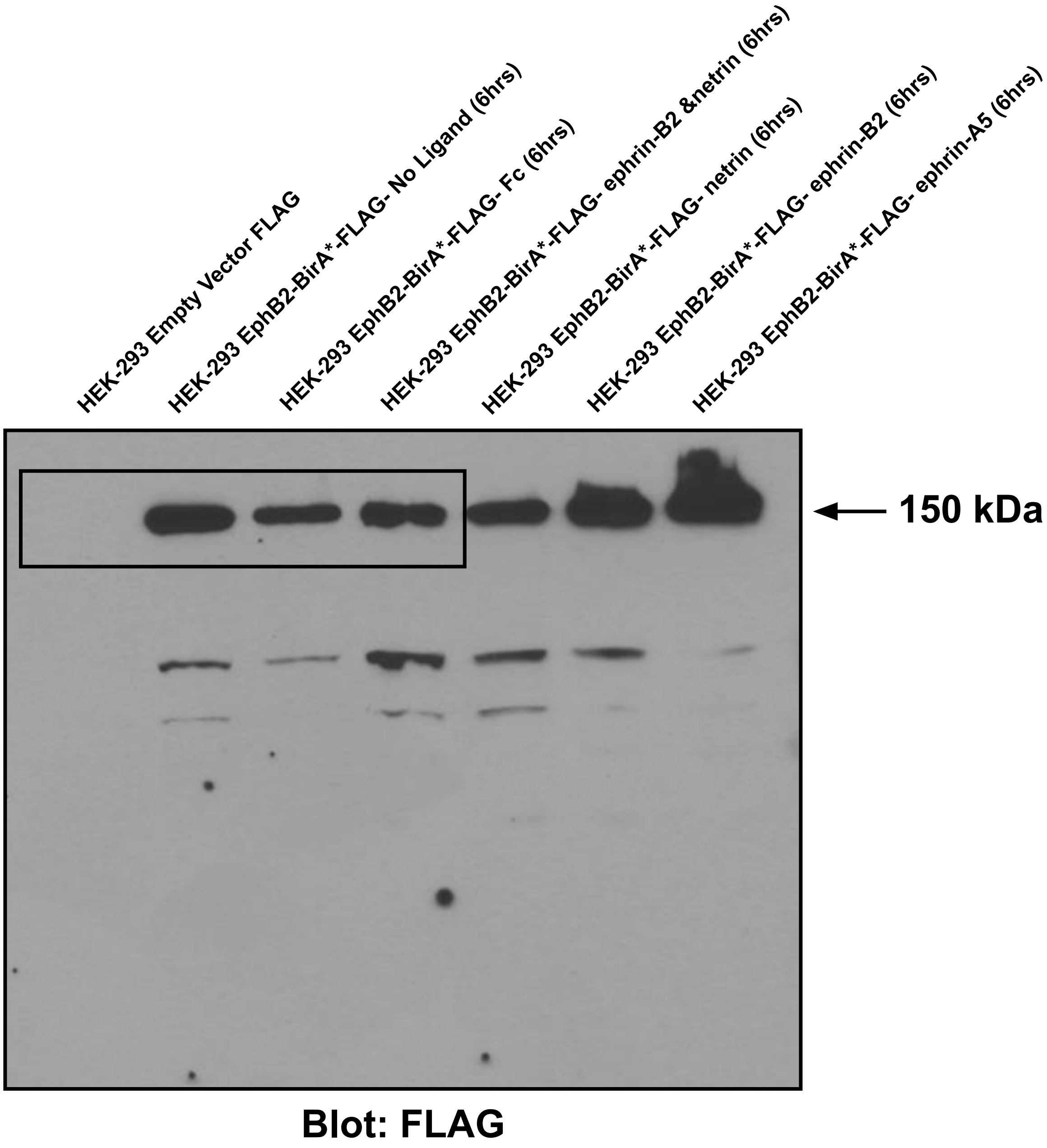

Supplementary Figure S6. Full Western blot in Figure 1b FLAG.

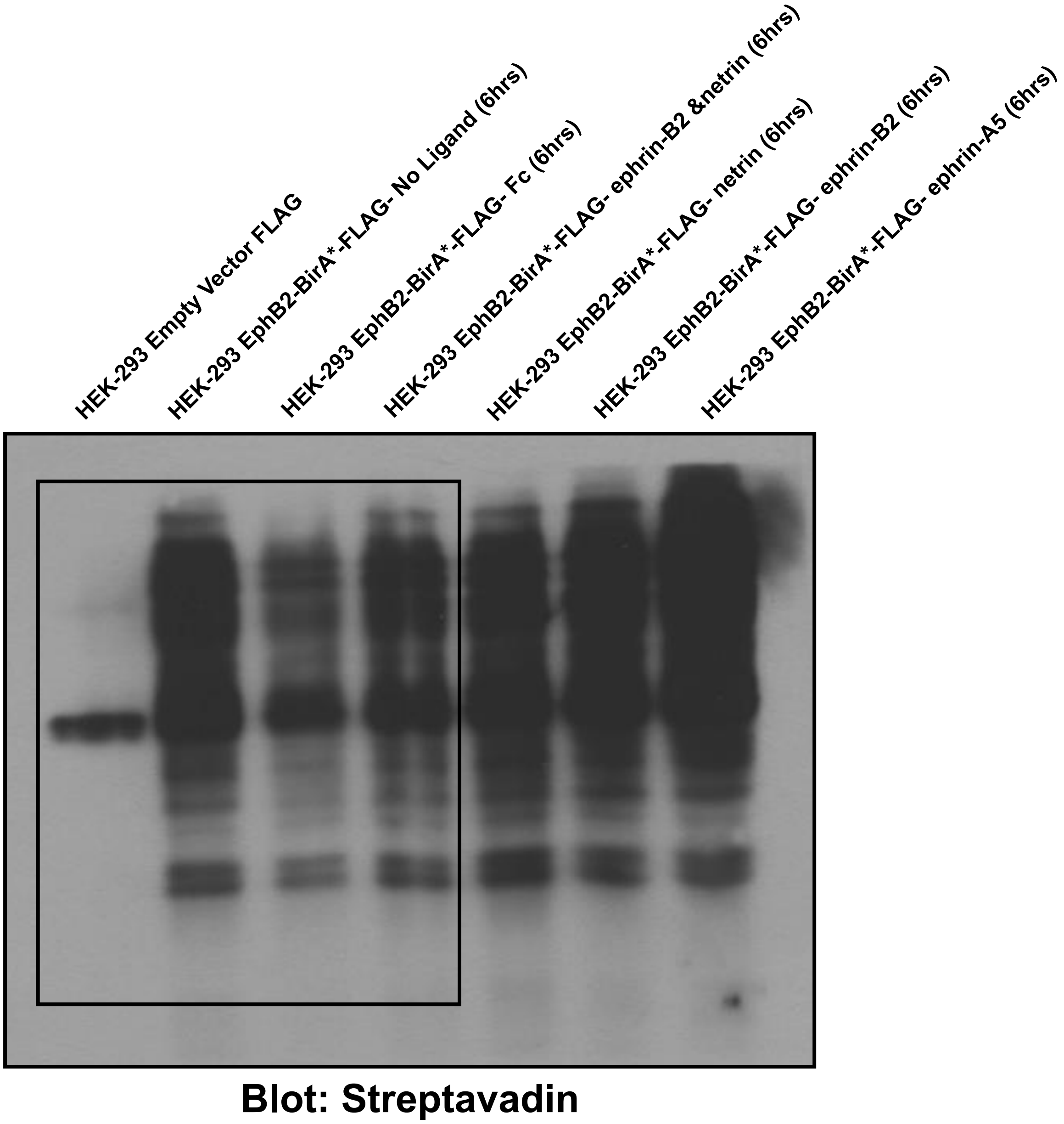

Supplementary Figure S7. Full Western blot in Figure 1b Streptavidin.

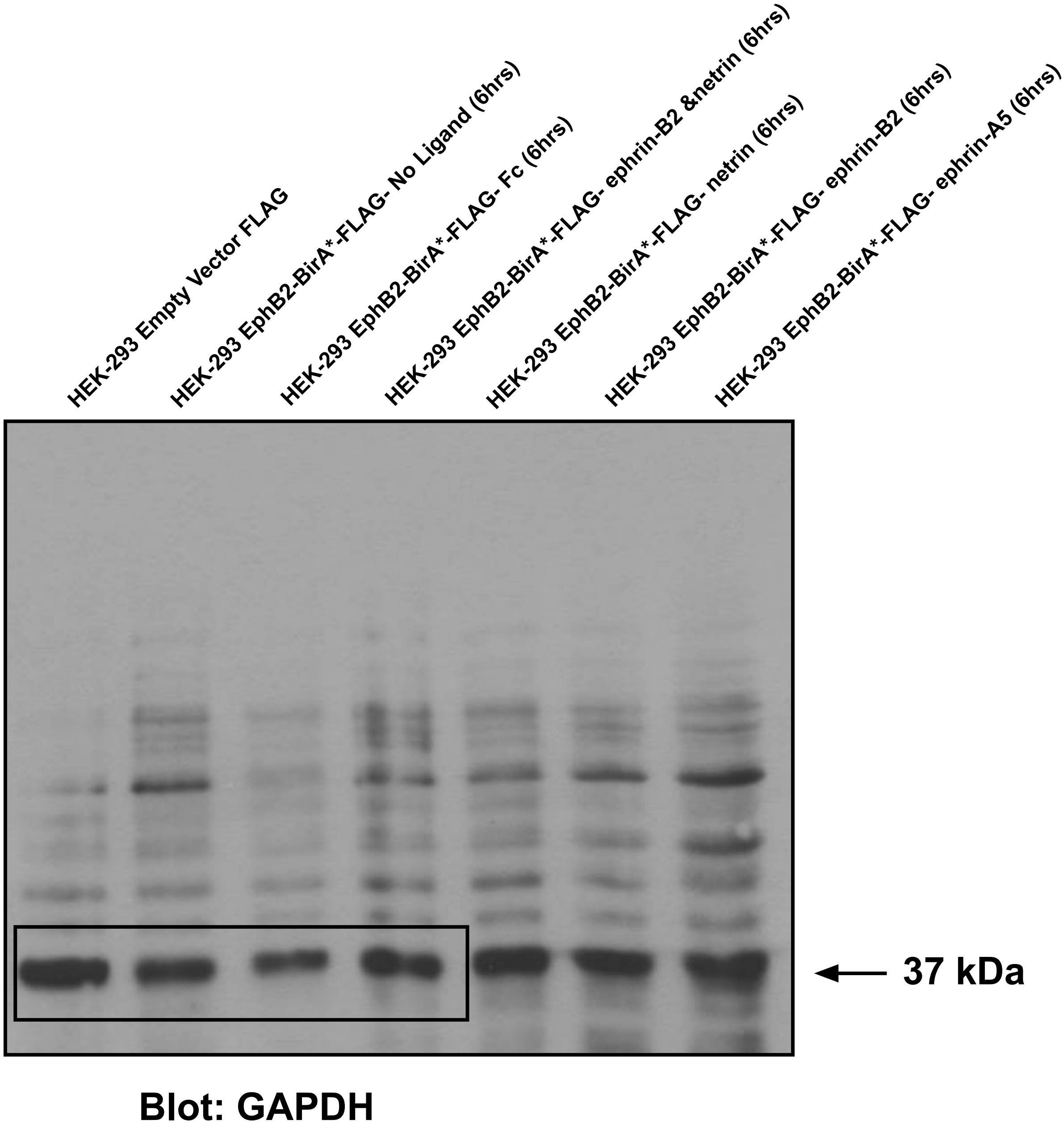

Supplementary Figure S8. Full Western blot in Figure 1b GAPDH.

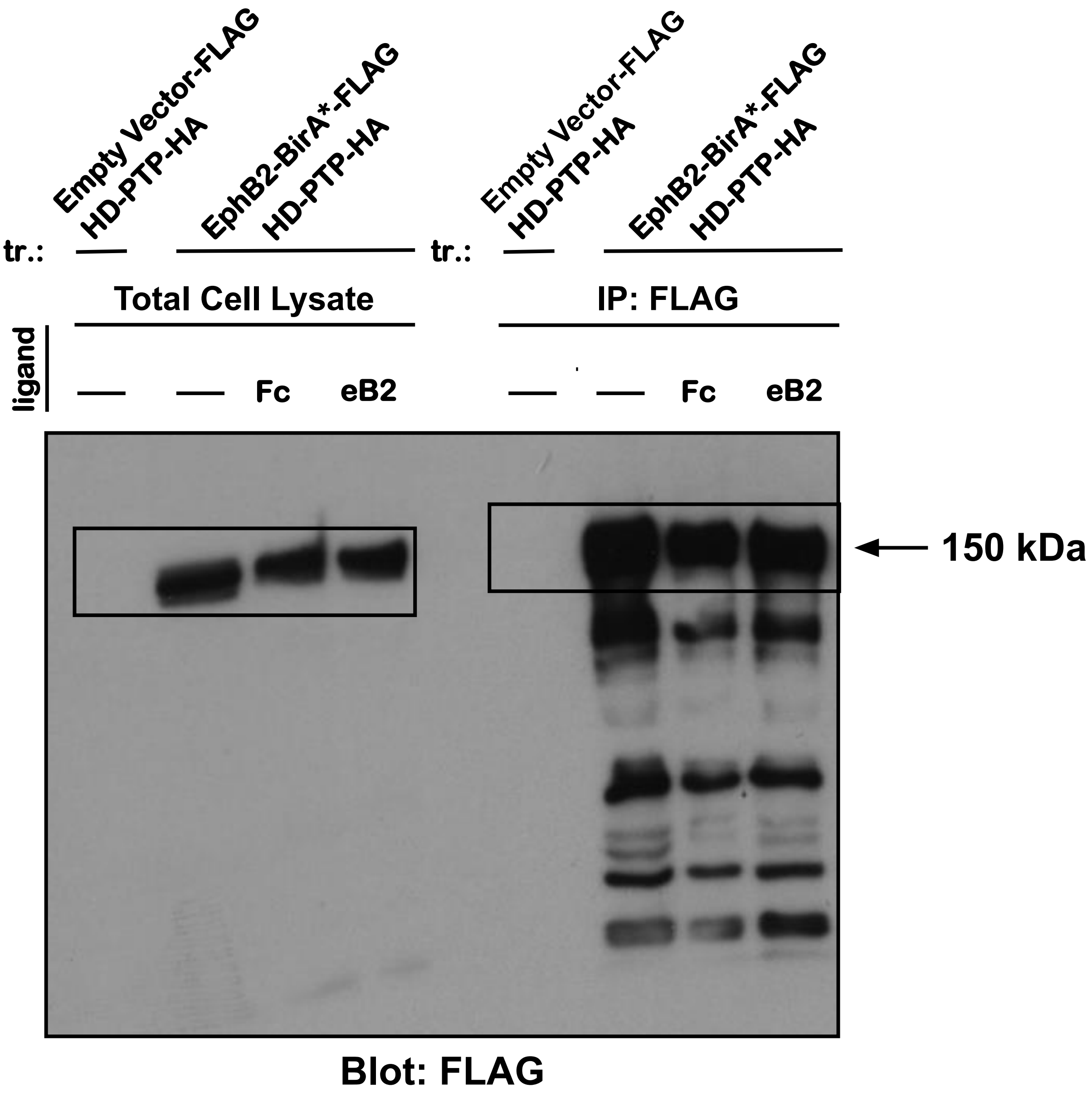

Supplementary Figure S9. Full Western blot in Figure 2a and Supplementary Figure S1a FLAG.

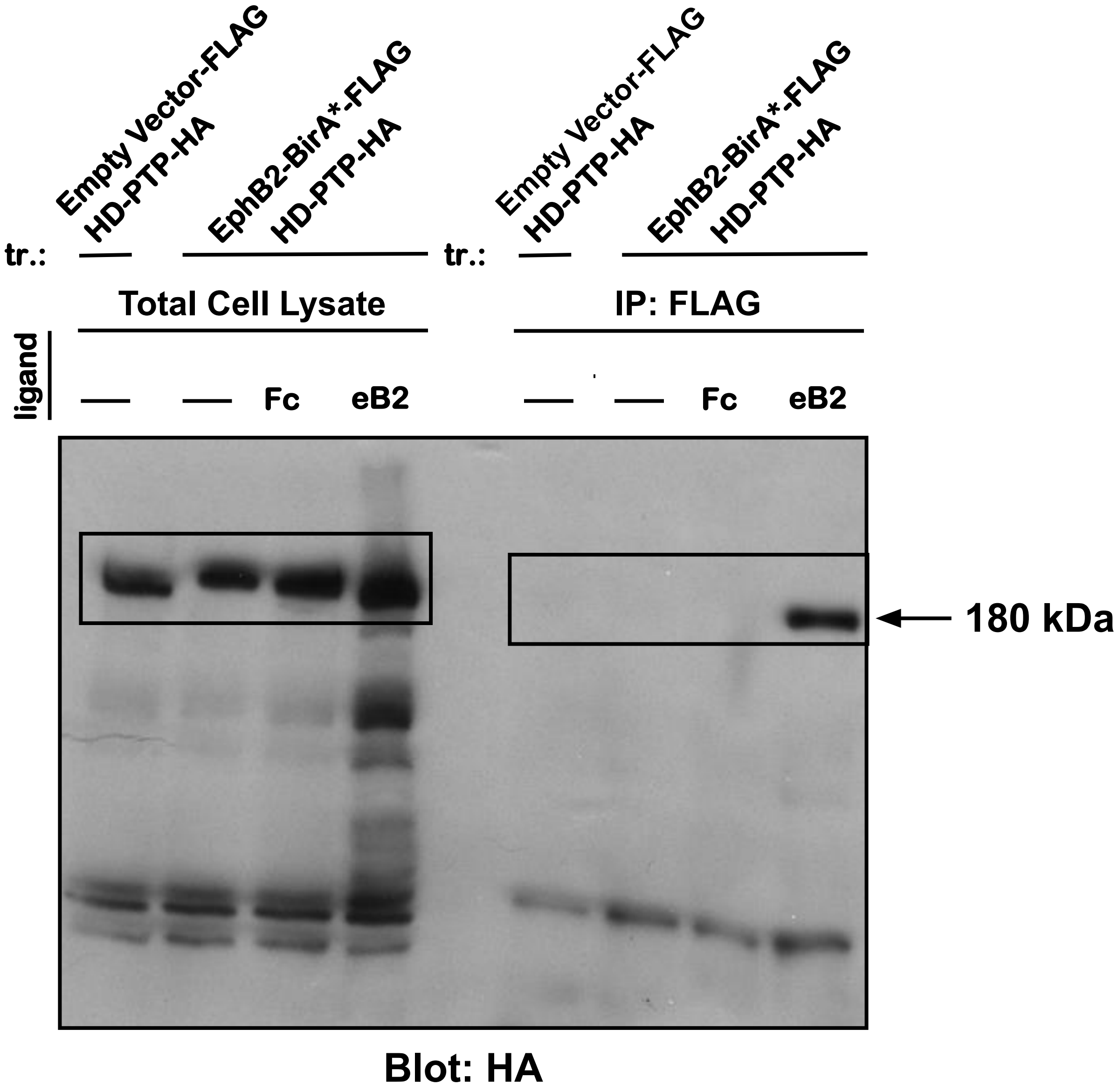

Supplementary Figure S10. Full Western blot in Figure 2a and Supplementary Figure S1c.

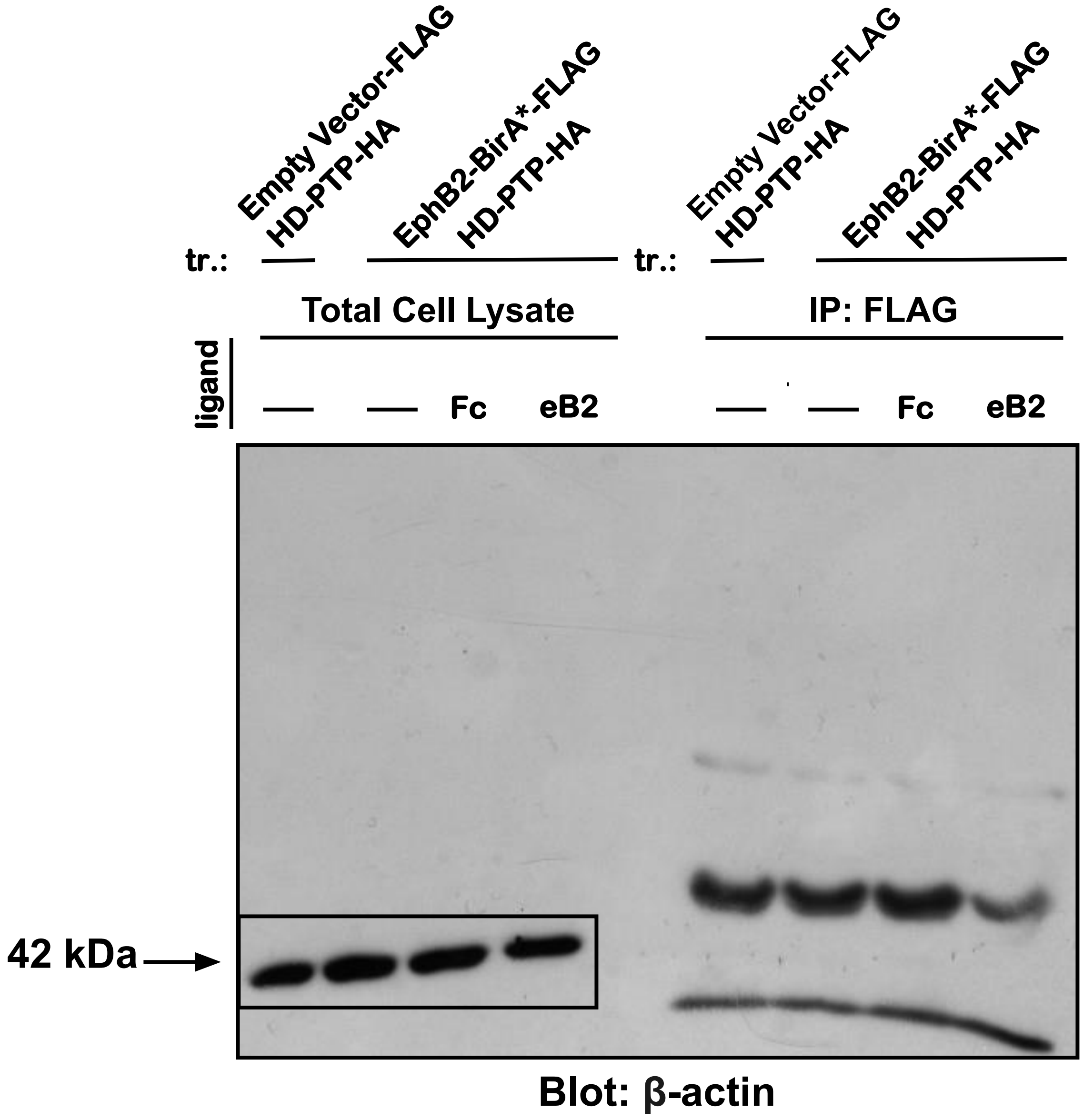

Supplementary Figure S11. Full Western blot in Supplementary Figure S1a beta-actin corresponding to the FLAG blot.

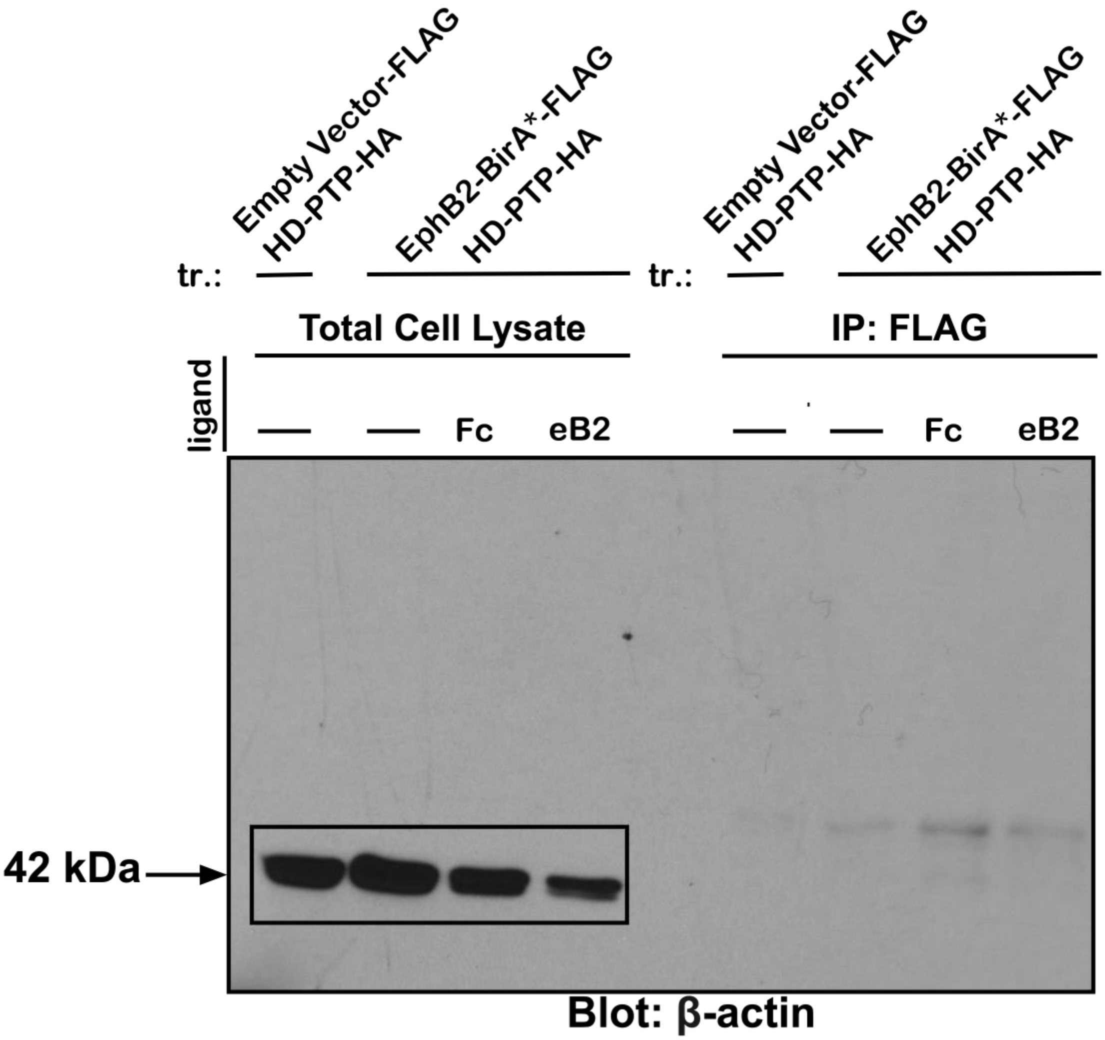

Supplementary Figure S12. Full Western blot in Supplementary Figure S1c beta-actin corresponding to the HA blot.

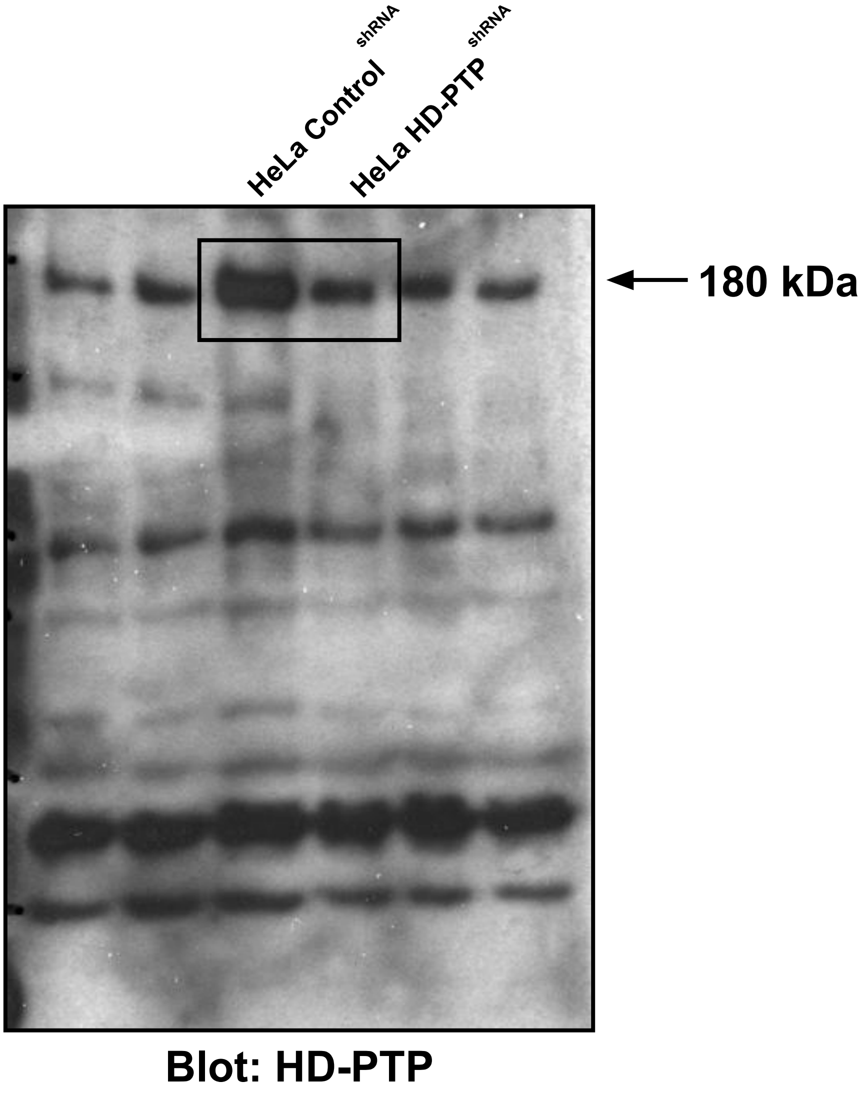

Supplementary Figure S13. Full Western blot in Supplementary Figure S1f HD-PTP.

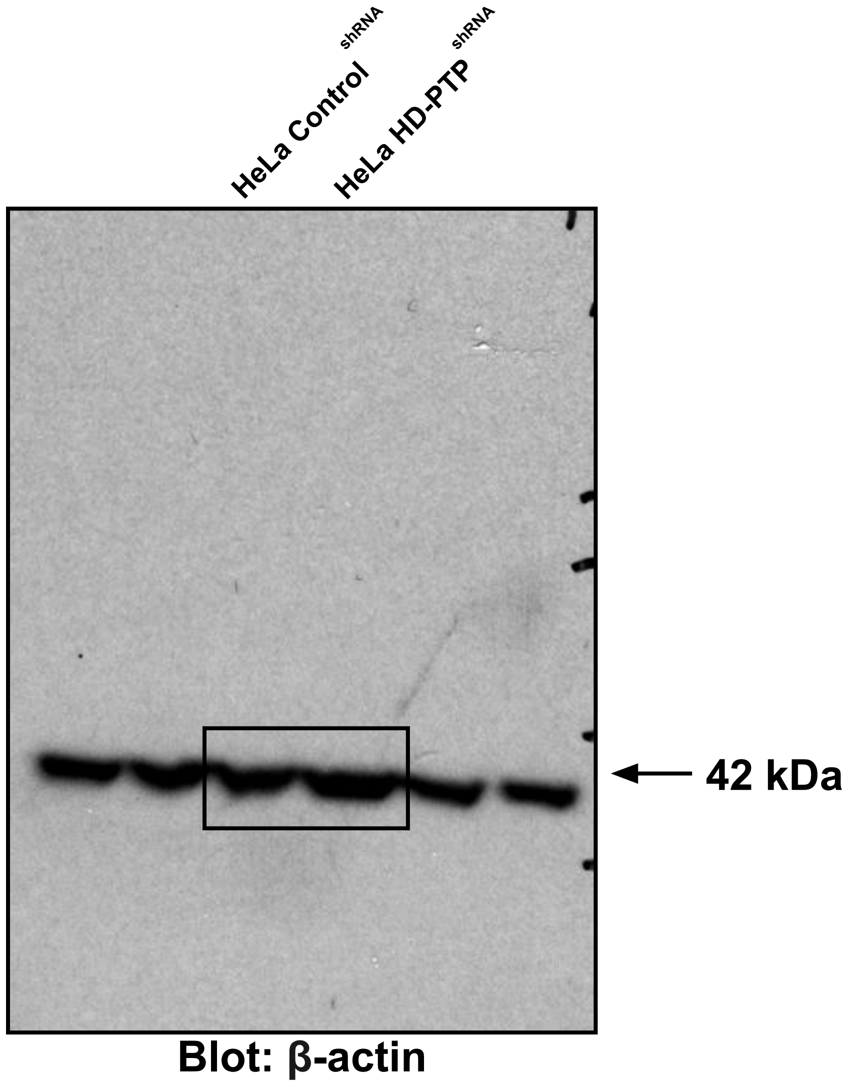

Supplementary Figure S14. Full Western blot in Supplementary Figure S1f Beta-actin.

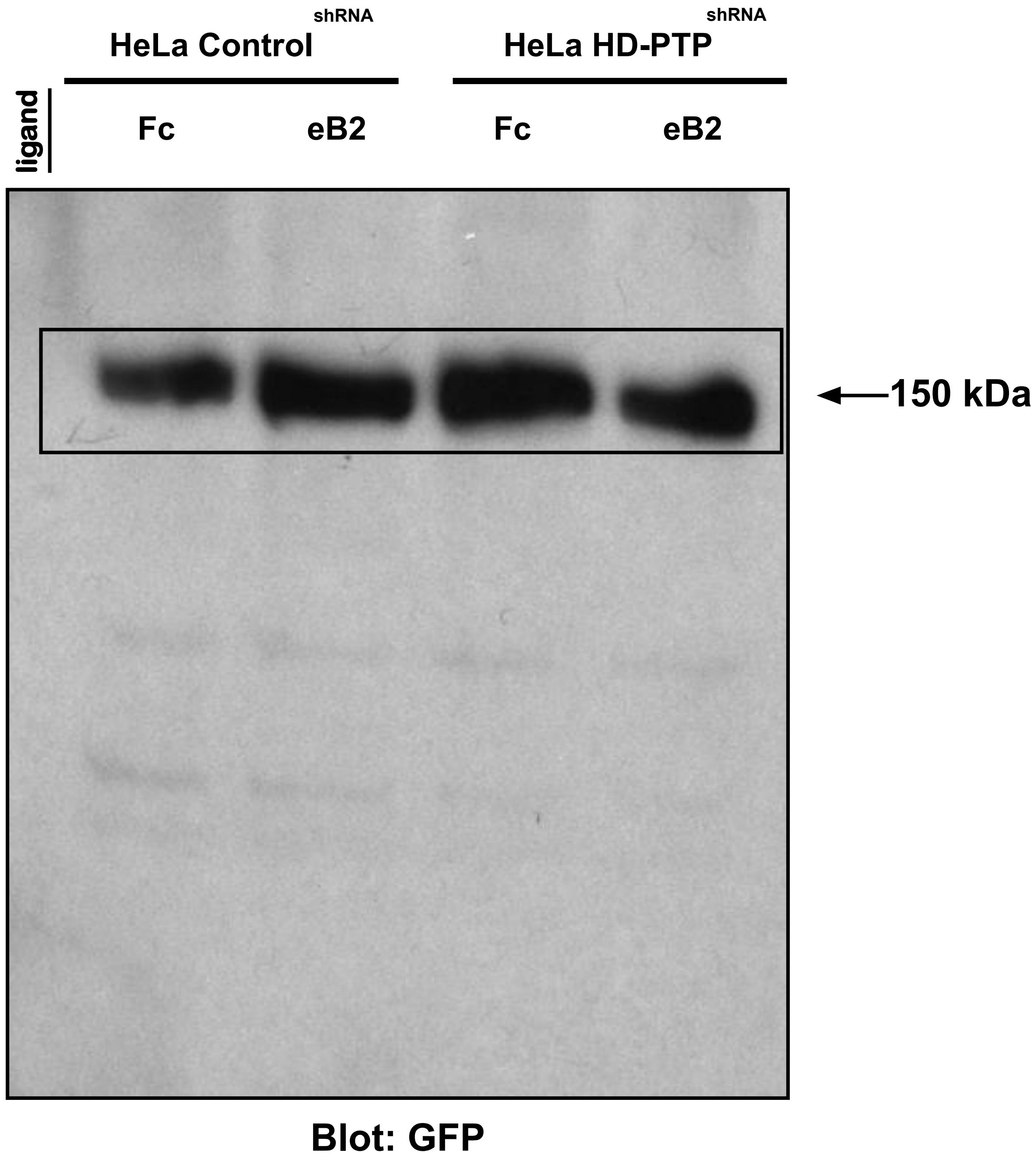

Supplementary Figure S15. Full Western blot in Supplementary Figure S4a GFP.

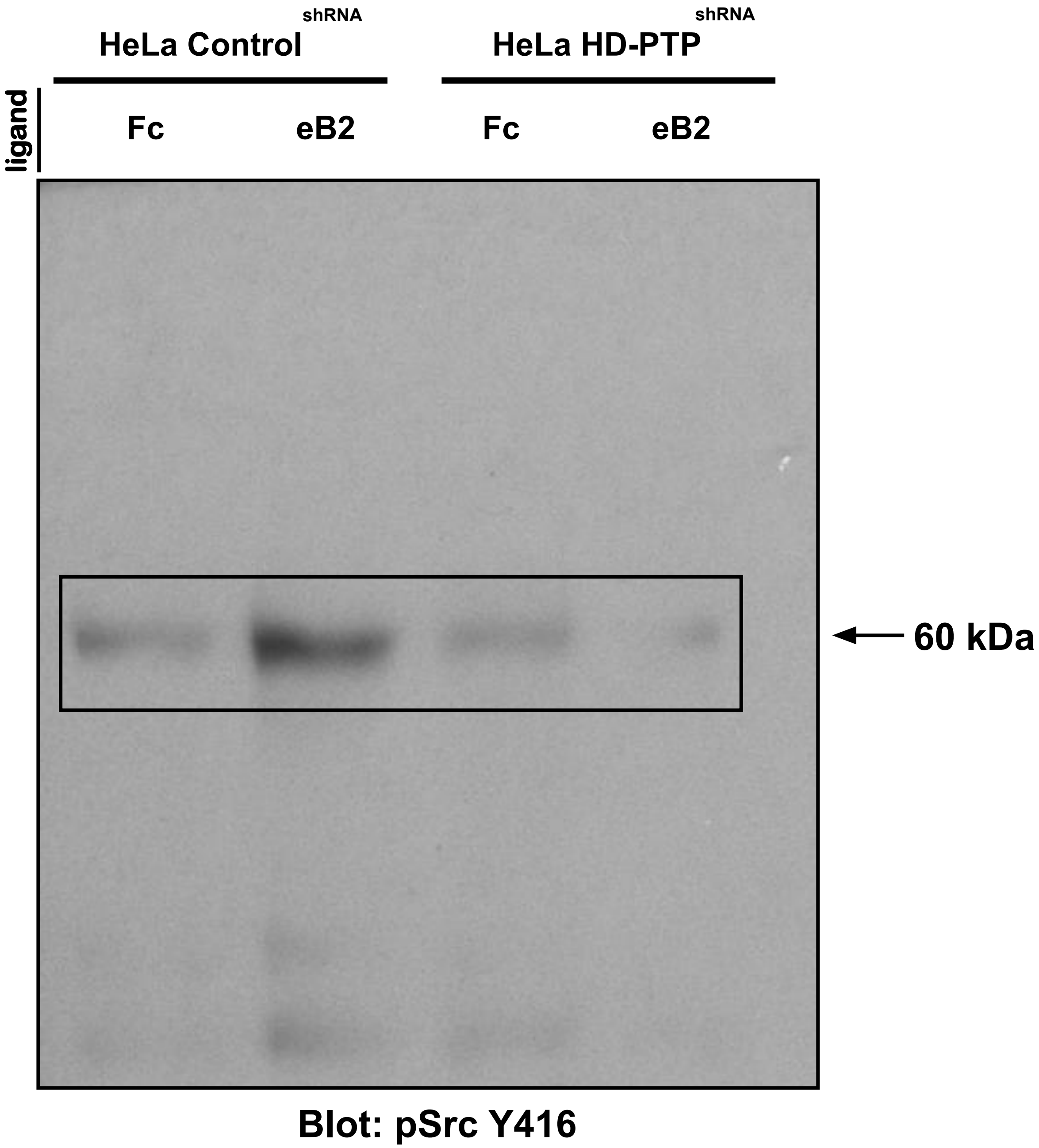

Supplementary Figure S16. Full Western blot in Supplementary Figure S4e Phospho-Src-Y416.

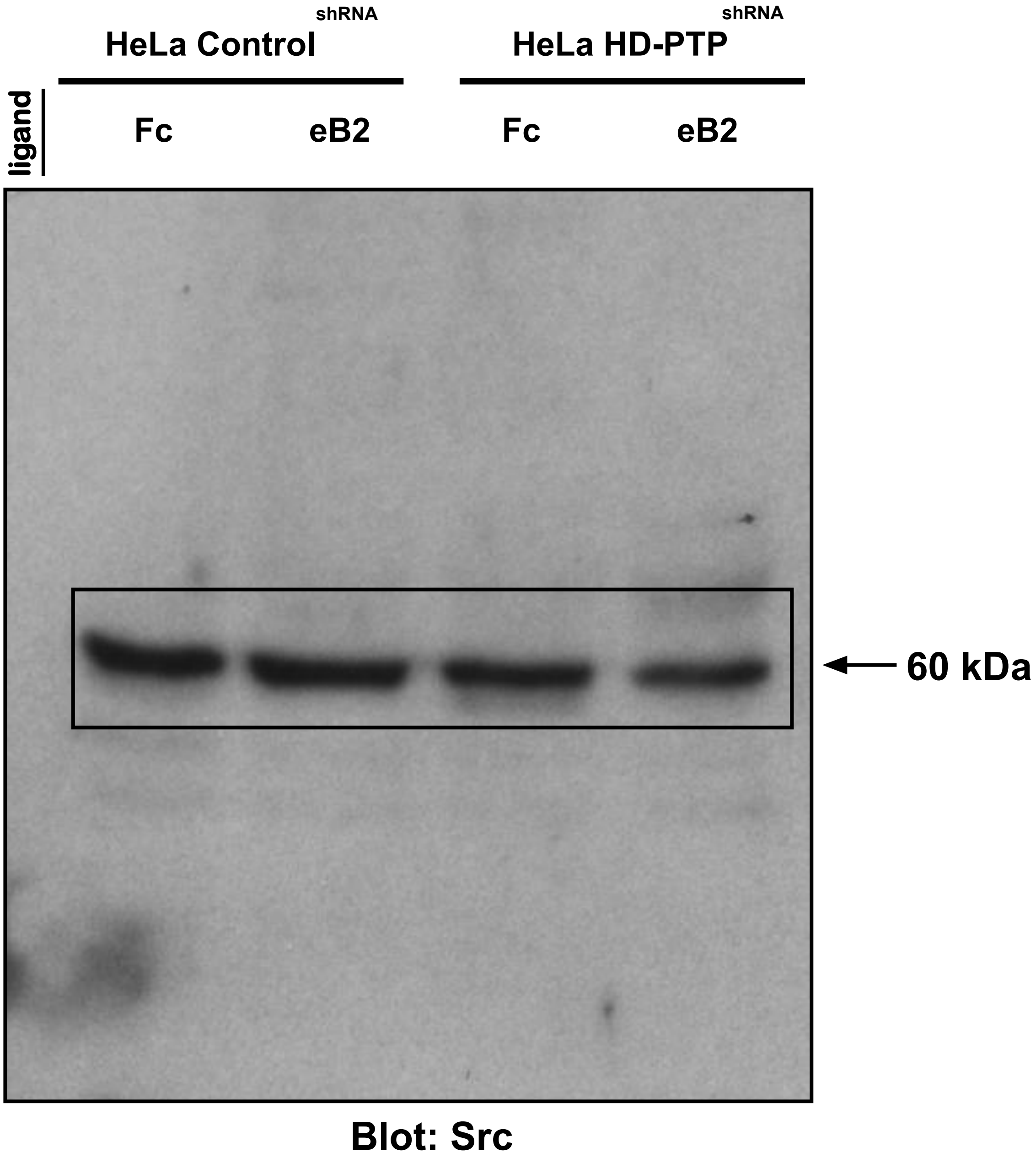

Supplementary Figure S17. Full Western blot in Supplementary Figure S4e Src.

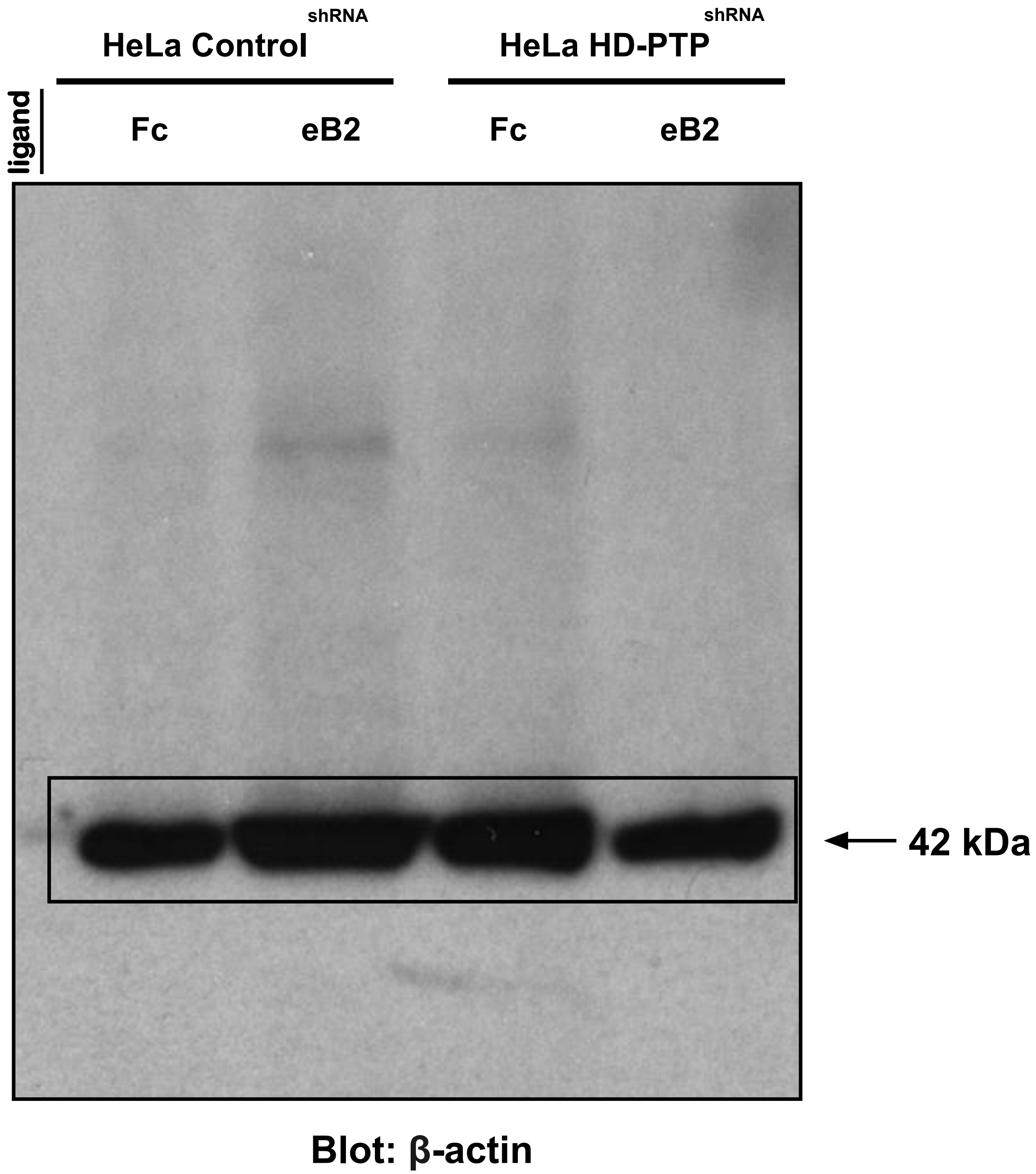

Supplementary Figure S18. Full Western blot in Supplementary Figure S4e Beta-actin corresponding to phospho-SRC Y416 blot.

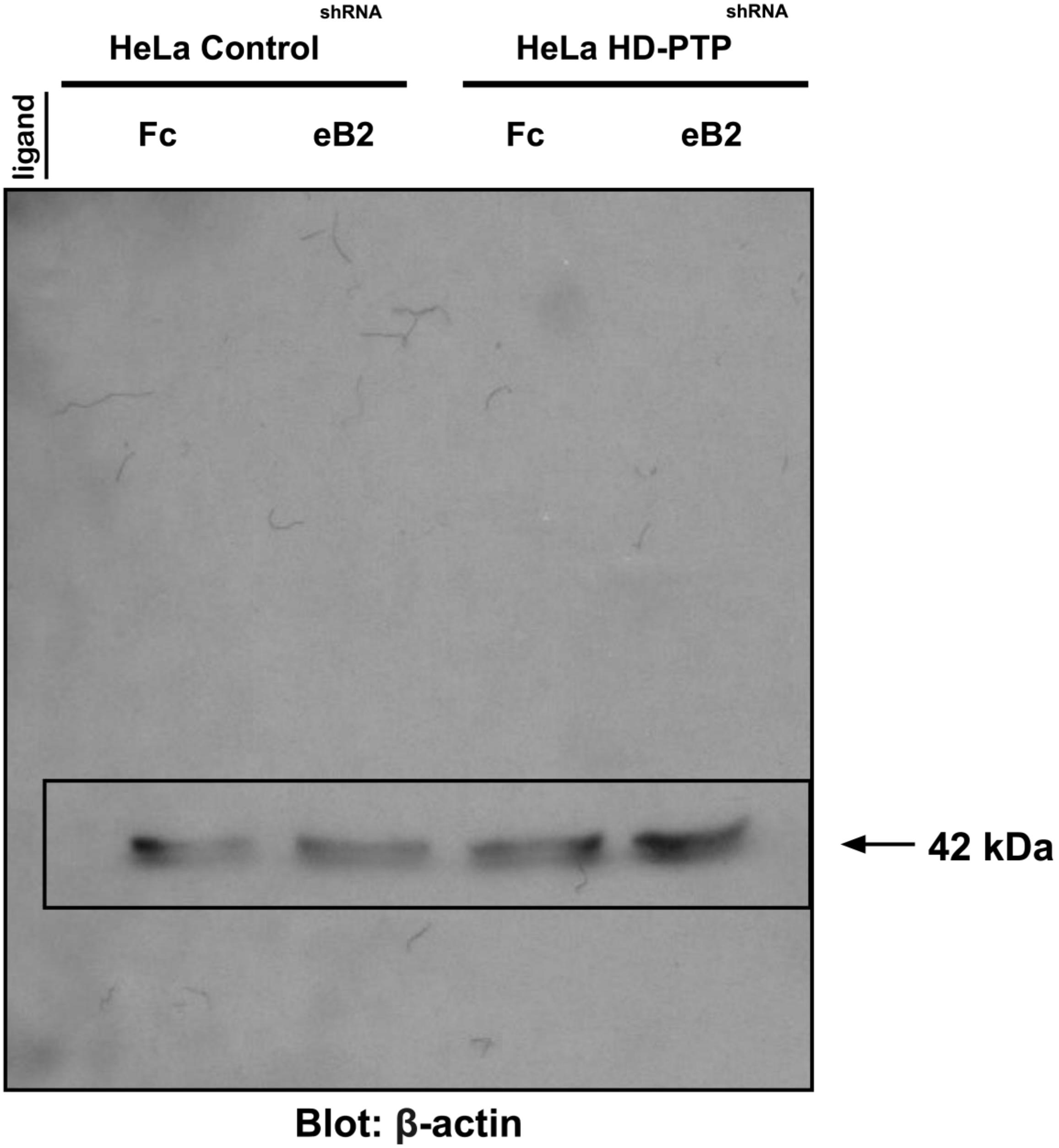

Supplementary Figure S19. Full Western blot in Supplementary Figure S4a Beta-actin corresponding to Src and GFP blot.

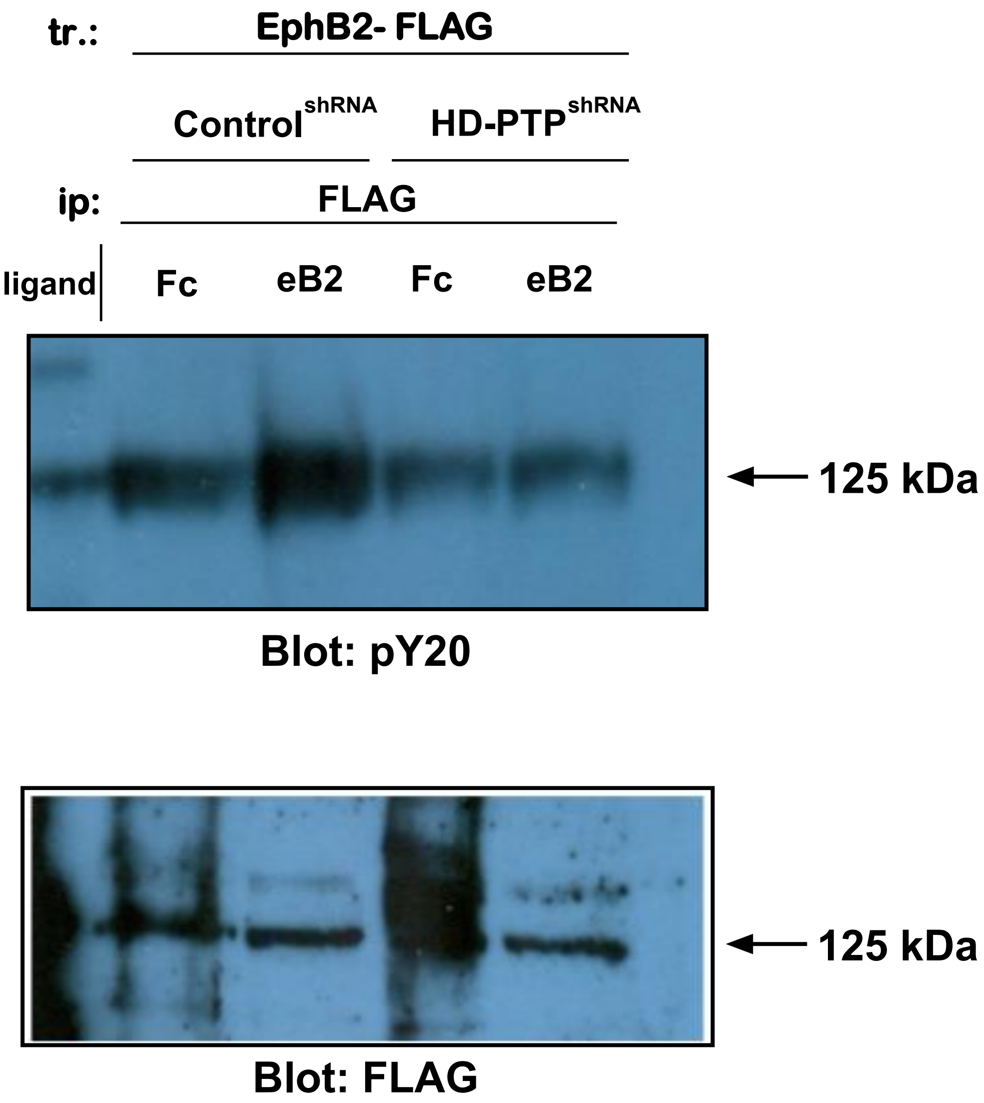

Supplementary Figure S20. Western blot in Figure 6g pY20 and FLAG. The membranes for this experiment were cut.

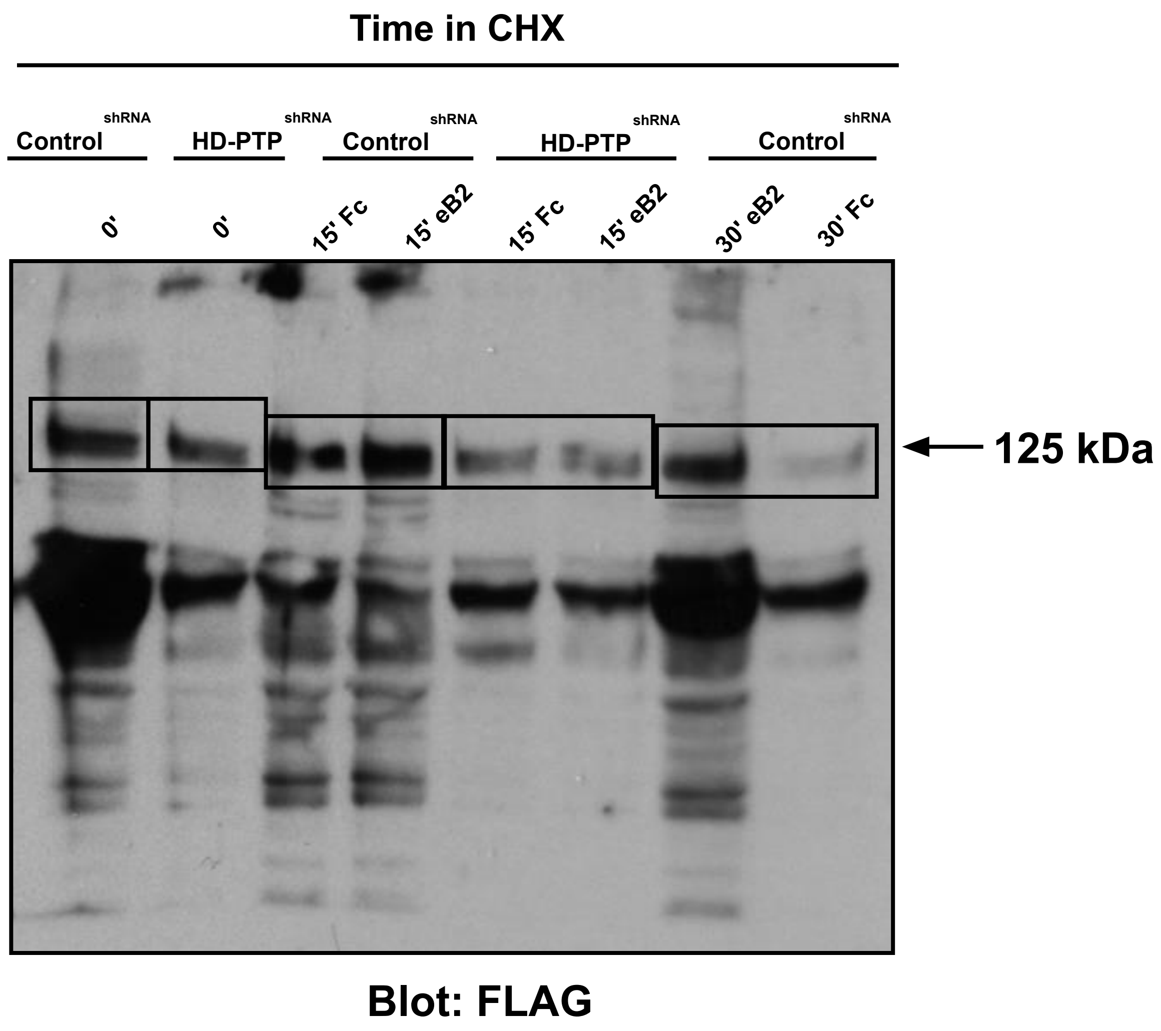

Supplementary Figure S21. Full Western blot in Figure 7e,g FLAG.

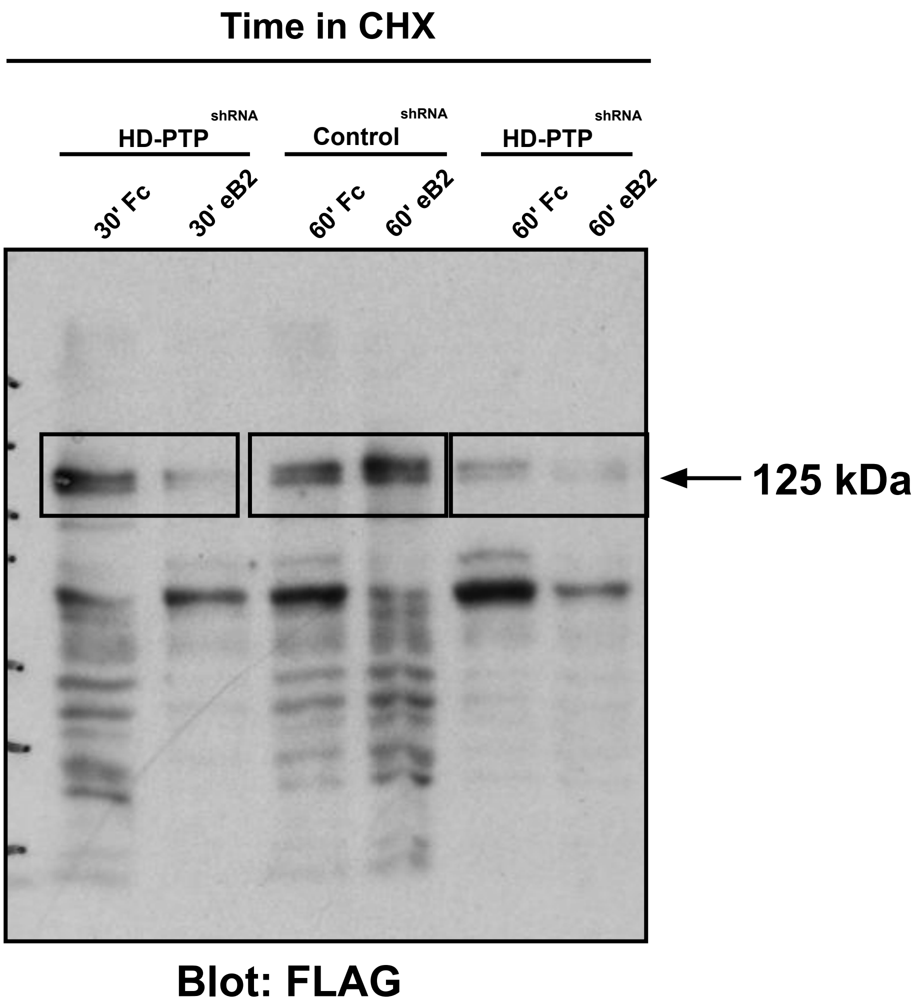

Supplementary Figure S22. Full Western blot in Figure 7e,g FLAG.

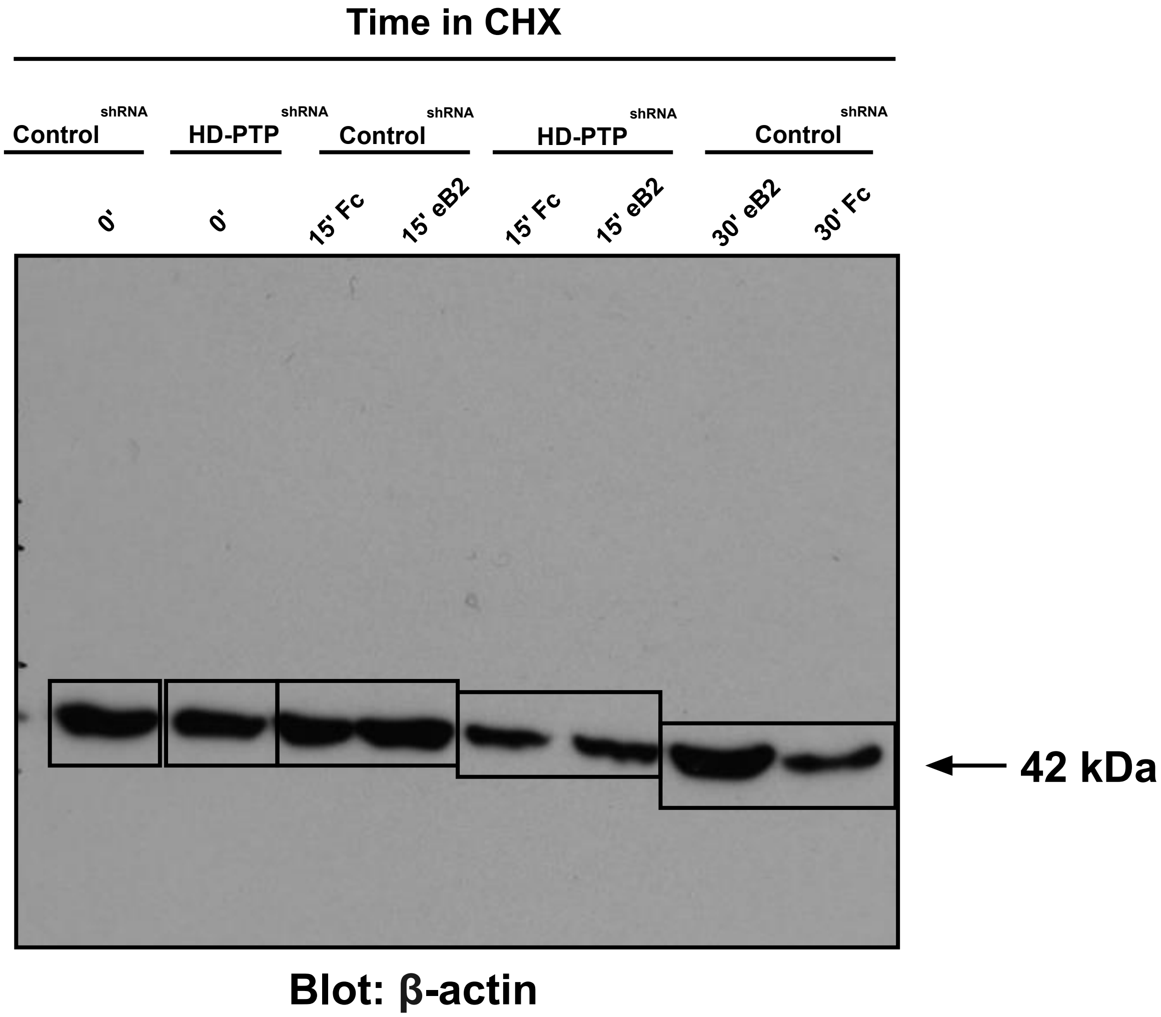

Supplementary Figure S23. Full Western blot in Figure 7e,g Beta-actin.

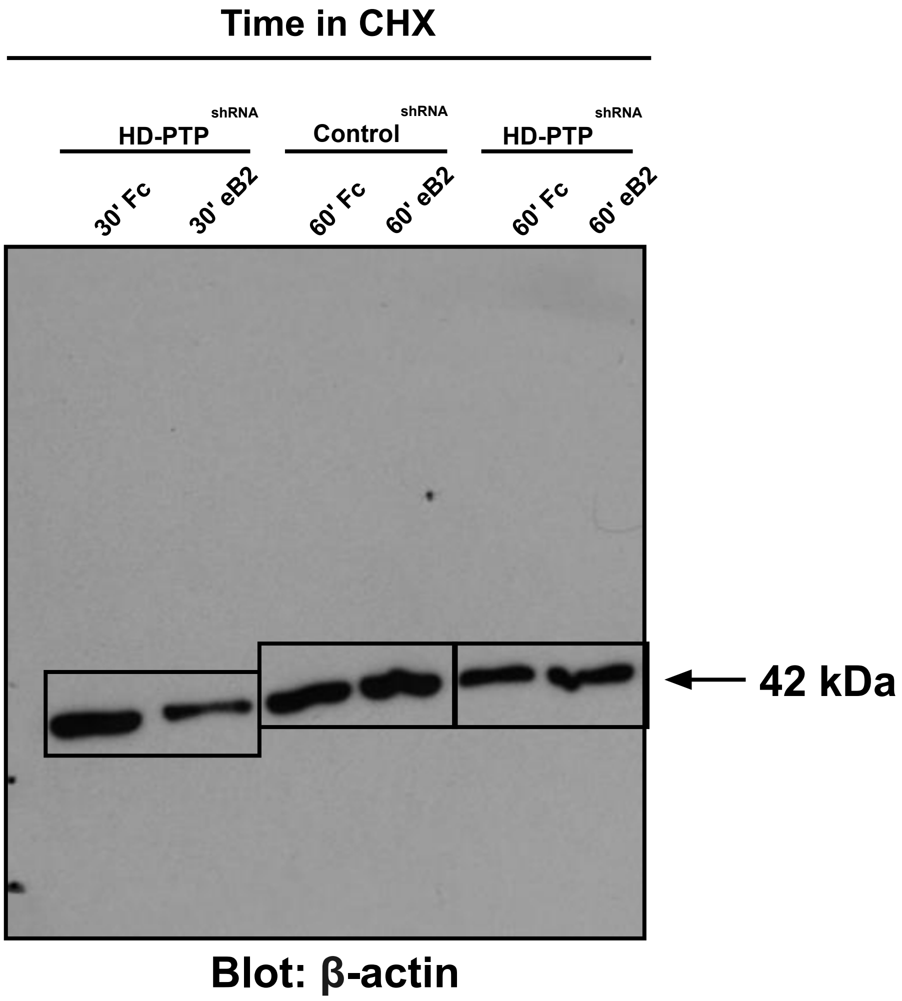

Supplementary Figure S24. Full Western blot in Figure 7e,g Beta-actin.

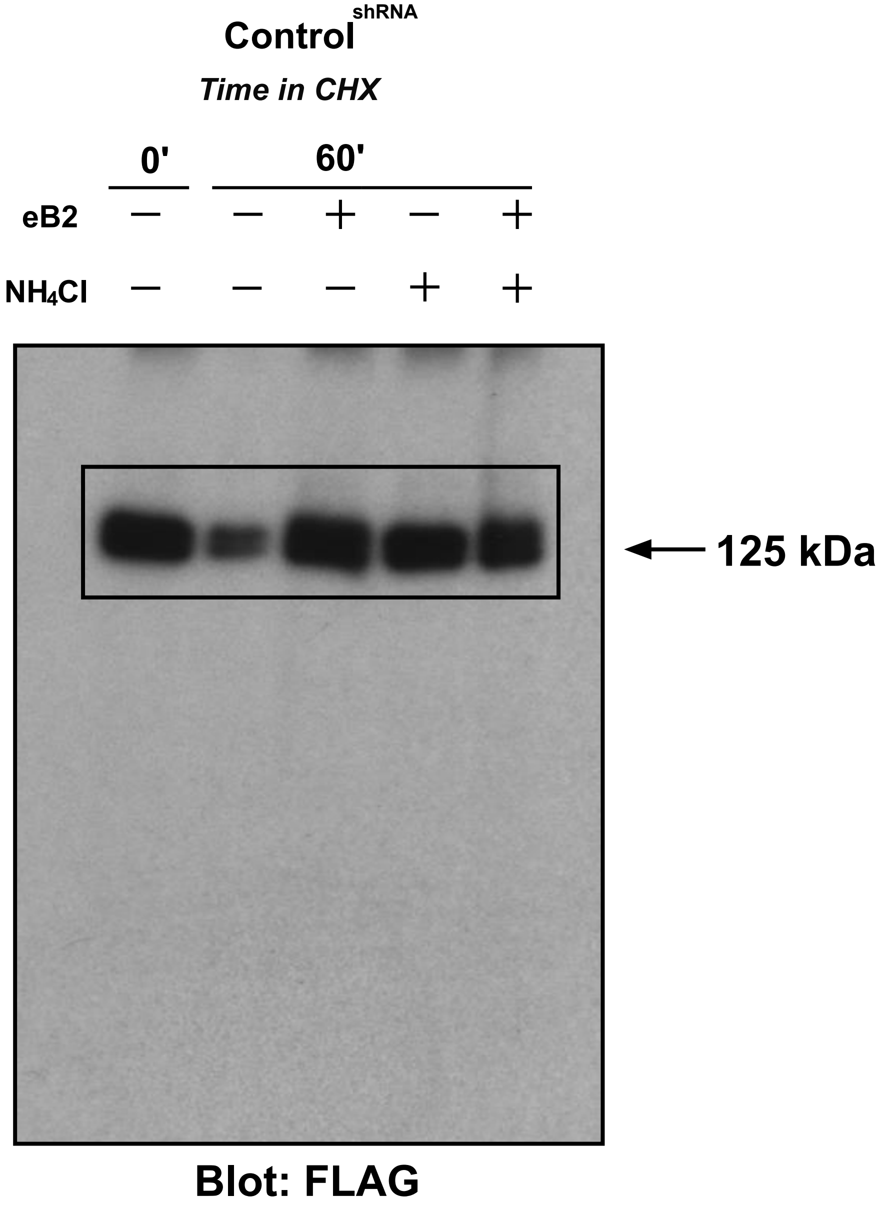

Supplementary Figure S25. Full Western blot in Figure 7i FLAG.

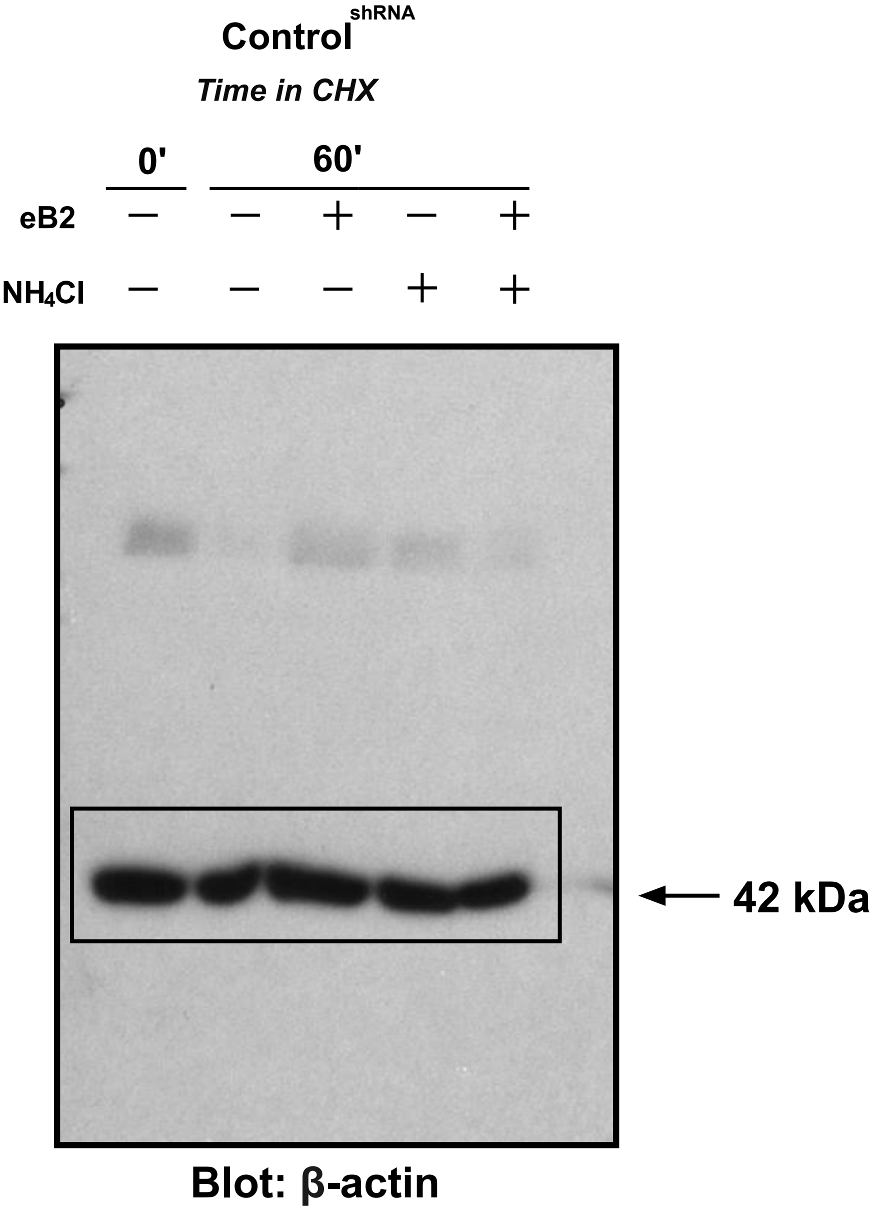

Supplementary Figure S26. Full Western blot in Figure 7i Beta-actin.

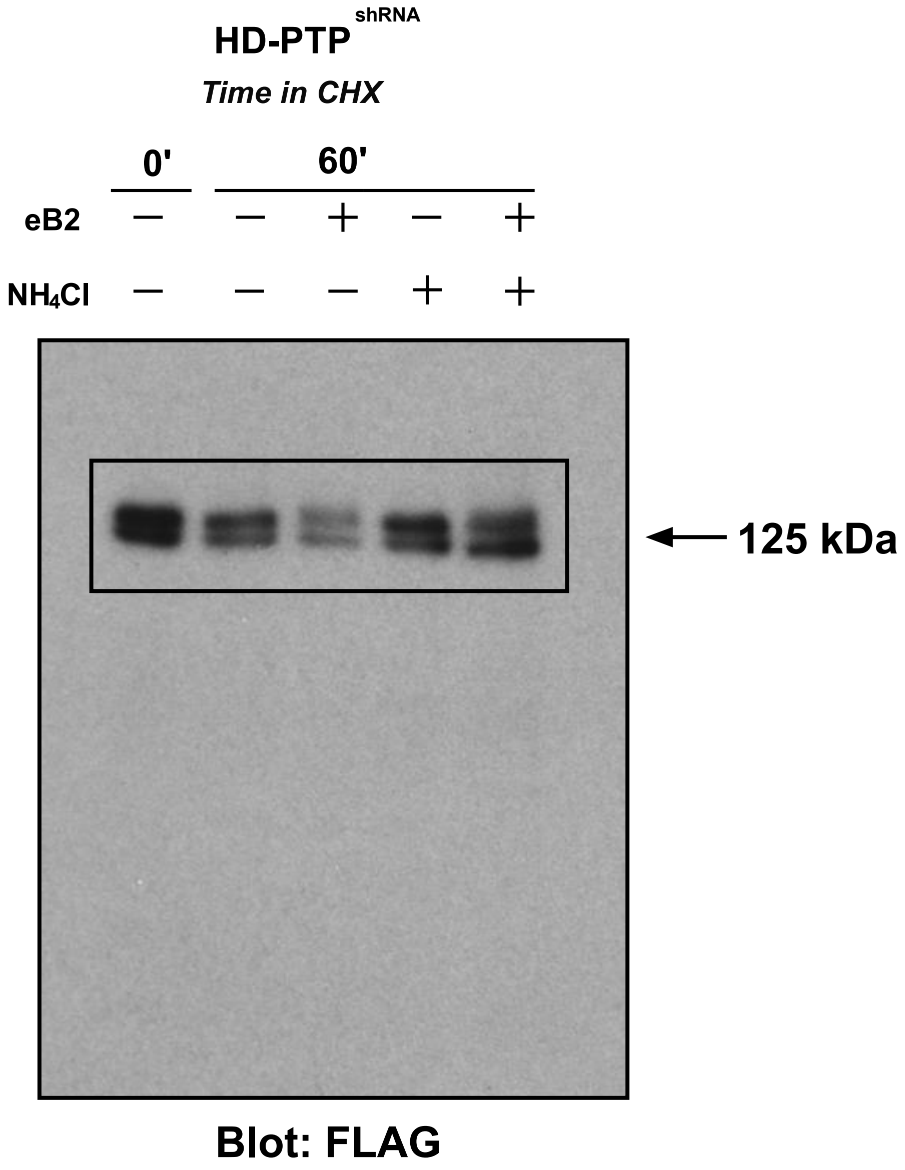

Supplementary Figure S27. Full Western blot in Figure 7k FLAG.

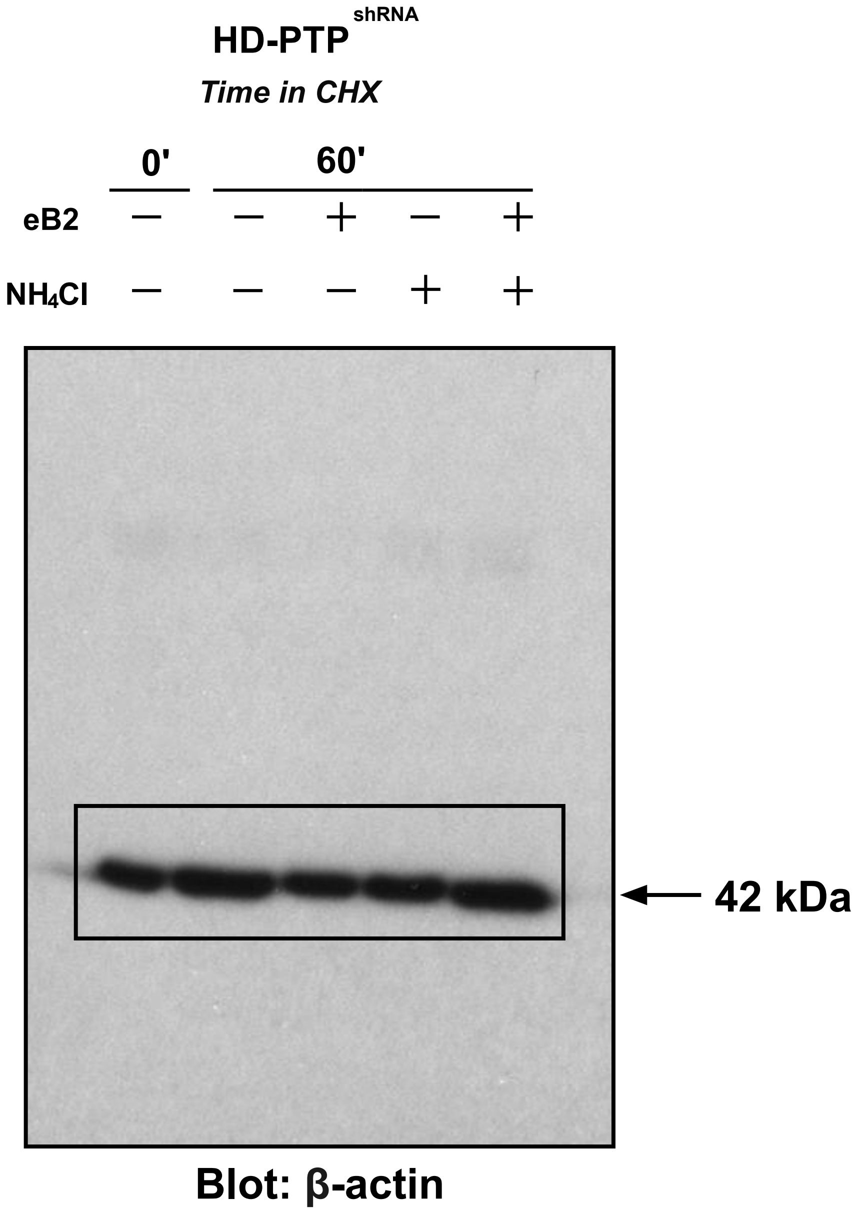

Supplementary Figure S28. Full Western blot in Figure 7k Beta-actin.

Supplementary Tables

Supplementary Table S1: Antibodies and reagents used.

**Antigen/ recombinant protein**	**Source Species**	**Dilution / concentration**	**Source/reference**
Foxp1	Rabbit	1:1000	Abcam
Isl1	Mouse	1:100	DSHB
GFP	Rabbit	1:1000	Invitrogen
Ephrin-B2-Fc	Mouse	CoIP = 1.5 µg/mL<br>HeLa = 1.0 µg/mL<br>Growth Cones = 10 µg/mL	R&D systems
Sema3A-Fc	Human	300 ng/mL	R&D Systems
Sema3F-Fc	Mouse	300 ng/mL	R&D Systems
Fc	Human	Matched with ephrin-B2 and Sema concentrations.	R&D Systems
EphB2	Goat	1:1000	R&D Systems
EEA1	Rabbit	1:1000	Abcam
Anti-Fc	Goat	1:4 mass ratio to ephrin-B2	Sigma Aldrich
Anti-Fc	Mouse	1:4 mass ratio to ephrin-B2	Sigma Aldrich
Tuj1	Mouse	1:1000	Covance
568-Phalloidin		1:500	Life Technologies
HA	Mouse	1:2000	Sigma Aldrich
Flag	Mouse	1:200	Sigma Aldrich
Flag-HRP	Mouse	1:8000	Sigma Aldrich
Beta-actin	Mouse	1:5000	Sigma Aldrich
HD-PTP	Rabbit	1:2000 (WB)<br>1:200 (IF)	[83]
Phosphotyrosine Y20	Mouse	1:2000	BD Biosciences
phospho-Y418-SFK	Rabbit	1:500	Life Technologies
Streptavidin-HRP	Mouse	1:25000	Sigma Aldrich
pSrc-Y416	Rabbit	1:1000	Cell Signaling
Src	Mouse	1:2000	Cell Signaling
GAPDH-HRP	Mouse	1:2000	Sigma Aldrich

Supplementary Table S2: Plasmids used.

**Plasmid**	**Species**	**Backbone**
EphA4 CRISPR	targeting Chick	pX3361
EphB2-GFP	Mouse	pN2-GFP
EphB2-FLAG	Mouse	pCMV
e[Isl1]::GFP	Chick	pBluescript
GFP	*Aequorea victoria*	pN2-GFP
HD-PTP CRISPR	targeting Chick (3 guides)	pX3361
HD-PTP-FLAG	Human	pcDNA3
HD-PTP(C/S)-FLAG	Human	pcDNA3
HD-PTP-HA	Human	pcDNA3

Supplementary Table S3: Cell lines used.

**Name**	**Parental Cell Type**	**Description**
Control HEK	Flp-In T-REx HEK293	Tetracycline inducible cell line expressing pcDNA5-pDEST-Empty Vector
EphB2-OE HEK	Flp-In T-REx HEK293	Tetracycline inducible cell line expressing pcDNA5-pDEST-EphB2-BirA*-FLAG
Control HeLa	Flp-In T-REx HeLa	Tetracycline inducible cell line expressing pcDNA5-pDEST-Empty Vector
EphB2-OE HeLa	Flp-In T-REx HeLa	Tetracycline inducible cell line expressing pcDNA5-pDEST-EphB2-BirA*-FLAG
Control^{shRNA} HeLa	HeLa	Lentiviral vector pLKO.1, selected with puromycin.
HD-PTP^{shRNA} HeLa	HeLa	Lentiviral vector shRNA targeting human HD-PTP pLKO.1, selected with puromycin.

Supplementary Table S4: Quantifications of Main & Supplementary figures. All values are expressed as mean±SD.

Figure 3b	Control^{shRNA} (Fc): 1648±314.0	Control^{shRNA} (eB2): 687.7±52.93
	HD-PTP^{shRNA} (Fc): 1573±36.94	HD-PTP^{shRNA} (eB2): 1200±80.93
Figure 3d	Control^{shRNA} (Fc): 1434±260.4	Control^{shRNA} (S3A): 505.9±87.06
	HD-PTP^{shRNA} (Fc): 1787±525.9	HD-PTP^{shRNA} (S3A): 603.0±58.15
Figure 4c	Control^{CRISPR}: 2393±398.5	HD-PTP^{CRISPR}: 790.4±51.20
Figure 4d	Control^{CRISPR}: 21889±1836	HD-PTP^{CRISPR}: 10931±1133
Figure 5a	Control^{CRISPR} (Fc): 17.75±4.856	Control^{CRISPR} (eB2): 85.00±2.582
	HD-PTP^{CRISPR} (Fc): 24.07±4.202	HD-PTP^{CRISPR} (eB2): 48.51±4.202
	Control^{CRISPR} (Fc): 17.13 ±1.887	Control^{CRISPR} (S3F): 91.38±2.250
	HD-PTP^{CRISPR} (Fc): 15.00±4.397	HD-PTP^{CRISPR} (S3F): 92.88 ±1.250
	HD-PTP^{CRISPR} + hHD-PTP (Fc): 18.00±2.309	
	HD-PTP^{CRISPR} + hHD-PTP (eB2): 83.50±4.123	
	HD-PTP^{CRISPR} + hHD-PTP C/S (Fc): 18.75±2.217	
	HD-PTP^{CRISPR} + hHD-PTP C/S (eB2): 82.50±2.082	
Figure 6b	Control^{CRISPR} (Fc): 5.642±4.487	Control^{CRISPR} (eB2): 52.39±3.250
	HD-PTP^{CRISPR} (Fc): 5.735±3.033	HD-PTP^{CRISPR} (eB2): 5.867±5.644
Figure 6d	Control^{shRNA} (Fc): 1.501±0.6270	Control^{shRNA} (eB2): 3.140±0.5684
	HD-PTP^{shRNA} (Fc): 1.119±0.2813	HD-PTP^{shRNA} (eB2): 0.9570±0.0663
Figure 6f	Control^{CRISPR} (Fc): 5.172±1.208	Control^{CRISPR} (eB2): 29.08±7.678
	HD-PTP^{CRISPR} (Fc): 4.673±2.886	HD-PTP^{CRISPR} (eB2): 25.76±5.827
Figure 6h	Control^{CRISPR} (Fc): 4.831±5.123	Control^{CRISPR} (eB2): 21.37±5.291
	HD-PTP^{CRISPR} (Fc): 4.069±1.409	HD-PTP^{CRISPR} (eB2): 5.673±4.208
	Control^{CRISPR} (Perm): 38.92±2.550	HD-PTP^{CRISPR} (Perm): 39.90±6.063
Figure 7b	Control^{CRISPR} (Fc): 20.79±1.850	Control^{CRISPR} (eB2): 29.85±3.073
	HD-PTP^{CRISPR} (Fc): 20.28±2.251	HD-PTP^{CRISPR} (eB2): 17.89±0.8117
Figure 7d	Control^{shRNA} (Fc): 11.03±1.630	Control^{shRNA} (eB2): 19.39±2.941
	HD-PTP^{shRNA} (Fc): 9.694±2.865	HD-PTP^{shRNA} (eB2): 11.67±3.955
Figure 7f	Control^{shRNA} (0’): 1.789±0.2663	
	Control^{shRNA} (Fc, 15’): 1.625±0.34	Control^{shRNA} (eB2, 15’): 1.524±0.281
	Control^{shRNA} (Fc, 30’): 1.066±0.045	Control^{shRNA} (eB2, 30’): 1.448±0.032
	Control^{shRNA} (Fc, 60’): 0.7943±0.038	Control^{shRNA} (eB2, 60’): 1.098±0.165

Figure 7h	HD-PTP^{shRNA} (0’): 1.753± 0.241	
	HD-PTP^{shRNA} (Fc, 15’): 1.108±0.078	HD-PTP^{shRNA} (eB2, 15’): 1.150±0.057
	HD-PTP^{shRNA} (Fc, 30’): 0.8521±0.024	HD-PTP^{shRNA} (eB2, 30’): 0.5884±0.087
	HD-PTP^{shRNA} (Fc, 60’): 0.5659±0.228	HD-PTP^{shRNA} (eB2, 60’): 0.08415±0.02
Figure 7j	Control^{shRNA} (0’): 1.574±0.07331	
	Control^{shRNA} (Fc CHX): 0.8841±0.1126	Control^{shRNA} (eB2 CHX): 1.100±0.08125
	Control^{shRNA} (Fc NH_4Cl): 1.295±0.2980	Control^{shRNA} (eB2 NH_4Cl): 1.313±0.2985
Figure 7l	HD-PTP^{shRNA} (0’): 1.537±0.1981	
	HD-PTP^{shRNA} (Fc CHX): 0.5636±0.1304	HD-PTP^{shRNA} (eB2 CHX): 0.2807±0.04096
	HD-PTP^{shRNA} (Fc NH_4Cl): 1.276±0.3053	HD-PTP^{shRNA} (eB2 NH_4Cl): 1.408±0.3349
Figure 8b	Control^{CRISPR}: 50.07±1.436	HD-PTP^{CRISPR}: 49.57±2.701
Figure 8c	Control^{CRISPR}: 90.75±5.497	HD-PTP^{CRISPR}: 90.98±5.235
Figure 8e	Control^{CRISPR} (dorsal %GFP): 7.00±4.06	
	Control^{CRISPR} (ventral %GFP): 93.00±4.06	
	HD-PTP^{CRISPR} (dorsal %GFP): 25.80±13.48	
	HD-PTP^{CRISPR} (ventral %GFP): 74.20±13.48	
Figure S1b	Empty Vector: 0.0458±0.0387	No Ligand: 1.893±0.3299
	Fc: 1.505±0.3059	eB2: 1.707±0.3652
Figure S1d	Empty Vector: 2.300±0.6790	No Ligand: 2.531±0.4903
	Fc : 2.258±0.6001	eB2 : 2.462±0.6999
Figure S1g	Control^{shRNA}: 1.260±0.2576	HD-PTP^{shRNA}: 0.6895±0.1811
Figure S1i	Control^{shRNA}: 11025±1484	HD-PTP^{shRNA}: 14315±2411
Figure S1j	Control^{shRNA}: 23811±1849	HD-PTP^{shRNA}: 24681±4852
Figure S3a	Control^{CRISPR}: 71.48±5.161	HD-PTP^{CRISPR}: 65.36±7.941
Figure S3c	Control^{CRISPR}: 12084±1380	HD-PTP^{CRISPR}: 11615±1264
	HD-PTP-OE: 13173±1269	
Figure S3h	Control^{CRISPR}: 11100±452.5	
	HD-PTP^{CRISPR} + hHD-PTP: 12244±1456	
	HD-PTP^{CRISPR} + hHD-PTP C/S: 12192±1407	
Figure S4b	Control^{shRNA} (Fc): 3.473 ±0.7610	Control^{shRNA} (eB2): 3.185 ±1.224
	HD-PTP^{shRNA} (Fc): 3.398 ±0.3151	HD-PTP^{shRNA} (eB2): 3.529 ±0.8591

Figure S4d	Control^{shRNA} (Fc): 11.52±1.270<br>HD-PTP^{shRNA} (Fc): 8.124±3.192	Control^{shRNA} (eB2): 26.44±4.077<br>HD-PTP^{shRNA} (eB2): 7.133±2.886
Figure S4f	Control^{shRNA} (Fc): 0.5007 ±0.1484<br>HD-PTP^{shRNA} (Fc): 0.3752 ±0.0385	Control^{shRNA} (eB2): 1.470 ±0.5673<br>HD-PTP^{shRNA} (eB2): 0.2512 ±0.1637
Figure S4g	Control^{shRNA} (Fc): 3.094 ±0.3629<br>HD-PTP^{shRNA} (Fc): 2.065 ±0.2201	Control^{shRNA} (eB2): 2.139 ±0.2712<br>HD-PTP^{shRNA} (eB2): 2.386 ±0.2592
Figure S4i	Control^{shRNA} (Fc): 0.3488±0.3369<br>HD-PTP^{shRNA} (Fc): 1.151±1.253	Control^{shRNA} (eB2): 43.96±4.716<br>HD-PTP^{shRNA} (eB2): 40.55±5.623
Figure S4k	Control^{shRNA} (Fc): 2.903±0.9730<br>HD-PTP^{shRNA} (Fc): 3.192±0.2961<br>Control^{shRNA} (Perm): 60.27±6.625	Control^{shRNA} (eB2): 36.55±4.791<br>HD-PTP^{shRNA} (eB2): 3.108 ±0.7202<br>HD-PTP^{shRNA} (Perm): 59.26±10.24
Figure S4m	Control^{shRNA} (Fc): 0.00±0.00<br>HD-PTP^{shRNA} (Fc): 0.00±0.00<br>Control^{shRNA} (Perm): 81.72±8.938	Control^{shRNA} (eB2): 0.00±0.00<br>HD-PTP^{shRNA} (eB2): 0.00±0.00<br>HD-PTP^{shRNA} (Perm): 85.14±3.633
Figure S4o	Control^{CRISPR} (Fc): 0.00±0.00<br>HD-PTP^{CRISPR} (Fc): 0.00±0.00<br>Control^{CRISPR} (Perm): 79.75±10.23	Control^{CRISPR} (eB2): 0.00±0.00<br>HD-PTP^{CRISPR} (eB2): 0.00±0.00<br>HD-PTP^{CRISPR} (Perm): 85.09±3.004
